# A Partial Correlation Screening Approach for Controlling the False Positive Rate in Sparse Gaussian Graphical Models

**DOI:** 10.1038/s41598-019-53795-x

**Published:** 2019-11-28

**Authors:** Ginette Lafit, Francis Tuerlinckx, Inez Myin-Germeys, Eva Ceulemans

**Affiliations:** 1 Research Group on Quantitative Psychology and Individual Differences, KU Leuven–University of Leuven, Leuven, 3000 Belgium; 2 Center for Contextual Psychiatry, KU Leuven–University of Leuven, Leuven, 3000 Belgium

**Keywords:** Human behaviour, Breast cancer, Post-traumatic stress disorder, Psychosis, Comorbidities

## Abstract

Gaussian Graphical Models (GGMs) are extensively used in many research areas, such as genomics, proteomics, neuroimaging, and psychology, to study the partial correlation structure of a set of variables. This structure is visualized by drawing an undirected network, in which the variables constitute the nodes and the partial correlations the edges. In many applications, it makes sense to impose sparsity (i.e., some of the partial correlations are forced to zero) as sparsity is theoretically meaningful and/or because it improves the predictive accuracy of the fitted model. However, as we will show by means of extensive simulations, state-of-the-art estimation approaches for imposing sparsity on GGMs, such as the Graphical lasso, ℓ_1_ regularized nodewise regression, and joint sparse regression, fall short because they often yield too many false positives (i.e., partial correlations that are not properly set to zero). In this paper we present a new estimation approach that allows to control the false positive rate better. Our approach consists of two steps: First, we estimate an undirected network using one of the three state-of-the-art estimation approaches. Second, we try to detect the false positives, by flagging the partial correlations that are smaller in absolute value than a given threshold, which is determined through cross-validation; the flagged correlations are set to zero. Applying this new approach to the same simulated data, shows that it indeed performs better. We also illustrate our approach by using it to estimate (1) a gene regulatory network for breast cancer data, (2) a symptom network of patients with a diagnosis within the nonaffective psychotic spectrum and (3) a symptom network of patients with PTSD.

## Introduction

**Figure 1:**
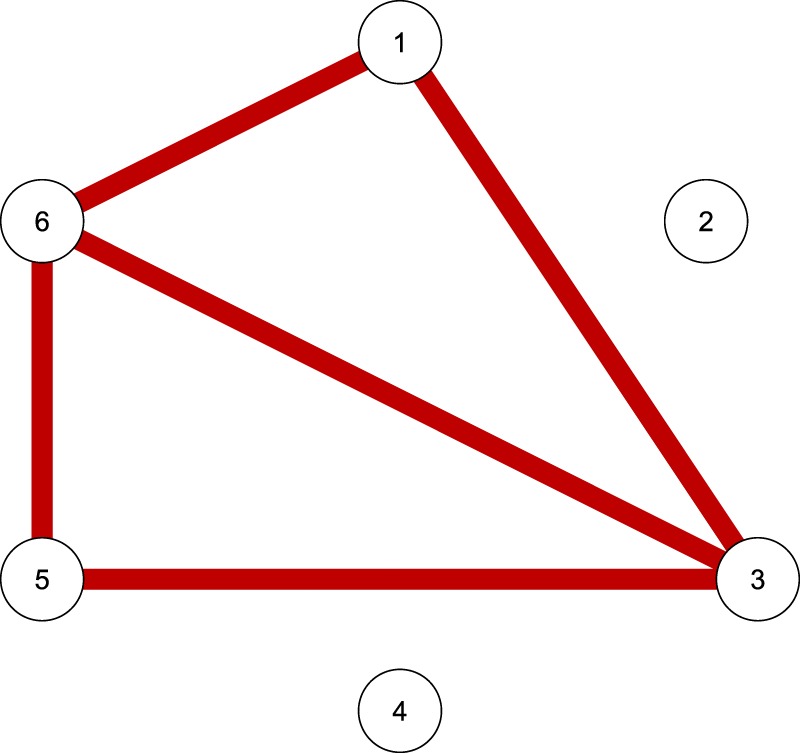
The undirected network implied by the toy example.

In many scientific disciplines, researchers are interested in the linear dependencies and unique relations between larger sets of variables, such as genes^1^, proteins^2^, symptoms of a disease^3^, functional brain connectivity^4^, etc. There is consensus that computing all pairwise correlations between these variables is misleading, because such correlations do not correct for linear relations that might be due to other variables. Therefore, many researchers recur to calculating partial correlation coefficients, which express the remaining linear dependency between two variables, after the effect of the rest of the variables under study is removed. More specifically, Gaussian Graphical Models (GGMs) have become increasingly popular^5,6^. These models yield an undirected network (i.e., undirected graph) in which the variables are depicted as nodes and the partial correlations among the variables are visualized as the edges among the nodes. The width of an edge reflects the size of the corresponding partial correlation (see Fig. 1).

Often, a sparse GGM is fitted, which implies that many of the partial correlations are forced to zero and thus that the corresponding edges in the network can be dropped. In some applications, the assumption of sparsity is intrinsic to the phenomenon under study. For instance, it has been shown that most genetic networks are sparse^7,8^. In other applications, the assumption of sparsity is motivated through improved interpretability. Indeed, even if the true model is not sparse the sparsity assumption allows to more accurately estimate the remaining parameters when the amount of information per parameter (*n*/*p*) is relatively small^9^, and prevents overfitting^10^.

Popular methods to estimate sparse GGMs are the regularized nodewise regression approach of Meinshausen and Bühlmann^11^, the joint sparse regression (SPACE) approach by Peng, *et al*.^12^ and the Graphical lasso (Glasso) proposed by Friedman, Hastie and Tibshirani^13^. These three approaches optimize different objective functions (see Methods section) but all set some of the estimated parameters, and thus some of the network edges, to zero through ${\ell }_{1}$ penalization. This penalization boils down to summing the absolute values of the estimated parameters and adding this sum to the objective function, after multiplying it by a regularization parameter. This parameter determines the impact of the penalty and has to be tuned by the user. Different tuning approaches have been proposed, based on cross-validation, information criteria, or finite sample derivations. Yet, ${\ell }_{1}$ penalization often does not work well. Indeed, recent studies on the use of ${\ell }_{1}$ penalization in standard regression analysis have shown that it tends to yield too many non-zero regression weights^14–16^. Translating these results to the estimation of sparse GGMs, we expect regularized nodewise regression, SPACE and the Glasso to often yield false positives, implying that some of the drawn edges should have been dropped. We will test this hypothesis in extensive simulations, in which we will also evaluate the effect of the tuning approach (i.e., information criteria, k-fold cross validation or finite sample derivations).

To overcome the problem of incorrectly included edges, we will present a novel approach, that we call Partial Correlation Screening (PCS). Our PCS approach consists of two steps. In the first step, we estimate a sparse partial correlation network using one of the state-of-the art methods mentioned above. In the second step, we try to filter out the false positives that will probably be present in the estimated network. To this end, we screen the resulting partial correlation matrix for values that are smaller in absolute value than a cross-validation based threshold and set these to zero. This novel approach is based on earlier work on thresholding after regularization. Specifically, Saligrama *et al*.^17^ and Descloux and Sardy^18^ proposed the idea of thresholding after applying an ${\ell }_{1}$ regularized procedure in the context of regression analysis. Ha and Sun^19^ presented a related idea for GGMs that consists of estimating the partial correlation matrix using a ridge penalty and then determining the non-zero entries of the matrix by hypothesis testing. Therefore, we will also evaluate what happens if we replace the ${\ell }_{1}$ penalty by a ridge penalty. We will apply the Partial Correlation Screening approach to the same simulated data to show that it indeed performs better. Finally, we will show how the PCS approach can be used to estimate networks based on real datasets: (1) a gene regulatory network of patients with breast cancer, (2) a symptom network of patients with a diagnosis within the nonaffective psychotic spectrum and (3) a symptom network of patients with Post-Traumatic Stress Disorder (PTSD).

The rest of the article is organized as follows. In the next section, we first present a toy example to introduce some notation and concepts and to illustrate that state-of-the-art estimation approaches yield networks that differ from the population model. Then, using this toy example we show how our PCS procedure works. Next, we discuss the results of two simulation studies, one based on settings that have been used in other papers on this topic and one based on the estimated network for a real data set. We present applications to real datasets. Next, we discuss our findings and formulate conclusions. Finally, the Methods section presents a detailed description of the evaluated tuning approaches for each of the state-of-the-art estimation approaches and of the PCS procedure.

## Results

### Toy example

The toy data consists of *n* = 100 observations that are sampled from a *p* = 6-dimensional multivariate Gaussian distribution. We set the covariance matrix of the distribution Σ to:

1 $$\Sigma =\left[\begin{array}{cccccc}1.63 & 0.00 & -0.70 & 0.00 & 0.63 & -0.70\\ 0.00 & 1.00 & 0.00 & 0.00 & 0.00 & 0.00\\ -0.70 & 0.00 & 1.68 & 0.00 & -0.70 & -0.13\\ 0.00 & 0.00 & 0.00 & 1.00 & 0.00 & 0.00\\ 0.63 & 0.00 & -0.70 & 0.00 & 1.63 & -0.70\\ -0.70 & 0.00 & -0.13 & 0.00 & -0.70 & 1.68\end{array}\right]$$

The conditional independence structure of this distribution can be represented by a GGM. The corresponding undirected network is shown in Fig. 1. The six variables *X*_1_ to *X*_6_ form the set of nodes *V* = {1, 2, 3, 4, 5, 6}. The set of edges *E* contains all node pairs (*i*, *j*) that are connected in the network, implying that *X*_*i*_ is conditionally dependent on *X*_*j*_, given all the remaining variables. Thus, variable pairs that do not belong to the edge set are conditionally independent, given all remaining variables. For instance, in this illustration, the network shows an edge between variables *X*_3_ and *X*_6_. Therefore, these variables are conditionally dependent. However, there is no edge between variables *X*_1_ and *X*_2_, implying that *X*_1_ and *X*_2_ are conditionally independent.

Because the variables are Gaussian distributed, a variable pair (*i*, *j*) is conditionally independent if and only if their partial correlation given the rest of the variables is zero^5^. Let’s denote by Γ the partial correlation matrix. The entries *ρ*_*ij*|*V*\{*i*, *j*}_ of this matrix are the partial correlations between variables *X*_*i*_ and *X*_*j*_, conditioned on the rest of variables. For the toy example the matrix Γ equals:

2 $$\Gamma =\left[\begin{array}{cccccc}1.00 & 0.00 & -0.45 & 0.00 & 0.00 & -0.45\\ 0.00 & 1.00 & 0.00 & 0.00 & 0.00 & 0.00\\ -0.45 & 0.00 & 1.00 & 0.00 & -0.45 & -0.45\\ 0.00 & 0.00 & 0.00 & 1.00 & 0.00 & 0.00\\ 0.00 & 0.00 & -0.45 & 0.00 & 1.00 & -0.45\\ -0.45 & 0.00 & -0.45 & 0.00 & -0.45 & 1.00\end{array}\right]$$

We can now define the neighborhood of each node. The neighborhood of node *i* consists of all the nodes *j* that form an edge with node *i*, implying that the partial correlation of *X*_*i*_ and *X*_*j*_ differs from zero. In the toy example the neighborhood of node 1 is formed by nodes 3 and 6, while the neighborhood of node 2 is empty.

Since the true edge set of the toy example is sparse, we can estimate it by means of the Glasso, SPACE, ${\ell }_{1}$ regularized nodewise regression (NR) and ridge nodewise regression (Ridge). Unlike Glasso and SPACE which directly estimate the edge structure, NR computes a regression model per node and thus yields two regression weights for each edge. To combine the information in these two weights into one edge, we can consider two variants, NR-AND and NR-OR. The AND rule means that an edge is only included in the model if both regression weights differ from zero, whereas the OR rule is more liberal and selects all edges for which at least one of the regression weights is not set to zero. Ridge estimates the partial correlations by fitting a regression model for each node using an ${\ell }_{2}$ penalty, which shrinks the regression weights towards zero.

For each of the estimation methods, a number of approaches have been put forward to tune the regularization parameter, the details of which are provided in the Methods section. For Glasso we will use 10-fold cross validation using two different loss functions: the first approach aims to minimize the negative log-likelihood function (CV1) and the second approach focuses on the sum of the prediction errors of each node (CV2). Moreover, we will apply two selection rules when using cross-validation: selecting the model that yields the lowest value and applying the one-standard-error-rule (1se)^20^. Additionally, we will consider the Bayesian Information Criterion (BIC) and the Extended Bayesian Information Criterion (EBIC)^21^. To tune the weight of the ${\ell }_{1}$ penalty term in SPACE and NR, we will apply 10-fold CV, its one-standard-error-rule variant, BIC and the finite sample result (FSR) proposed by Meinshausen and Bühlmann^11^. Note that in NR the tuning is performed for each separate regression. To optimize the weight of the ${\ell }_{2}$ penalty term in Ridge we will apply 10-fold CV for each separate regression. We note that this considered set of procedures is not intended to be exhaustive. Yet, the set is sufficient to illustrate the problem of efficiently tuning the penalty weight when there is limited information.

**Figure 2:**
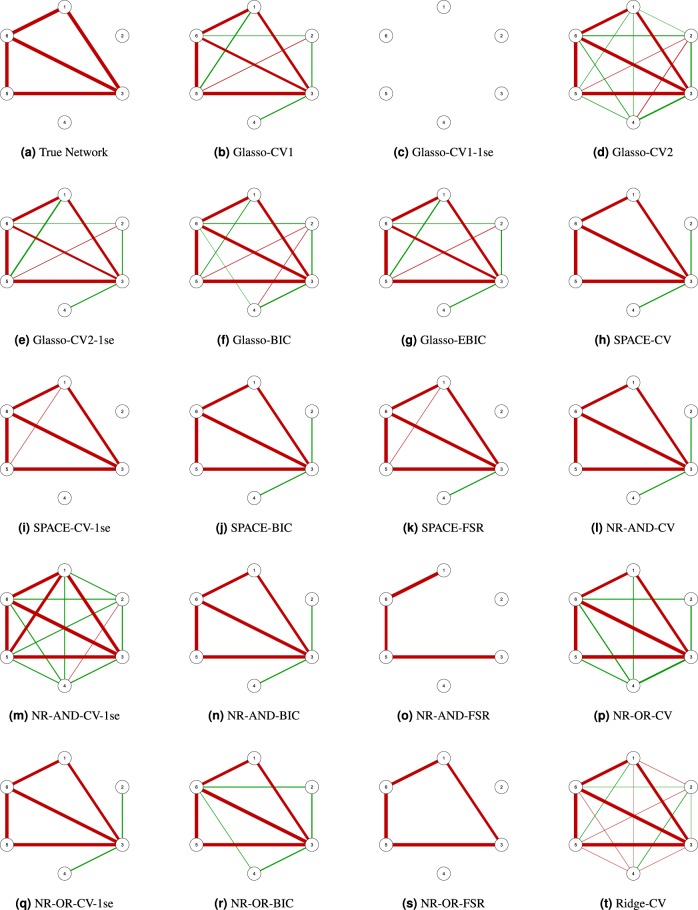
Estimated undirected networks for the toy example, before applying PCS.

Figure 2 shows the GGMs obtained with the nineteen considered approaches (i.e., nineteen combinations of estimation method (Glasso, SPACE, NR-AND, NR-OR and Ridge) and tuning options (CV, CV-1se, BIC, EBIC, FSR). We observe that Glasso-CV1-1se (panel c), NR-AND-FSR (panel o) and NR-OR-FSR (panel s) yield a network that is more sparse than the true network. Applying Glasso-CV1-1se all edges are set to zero. Whereas with NR-AND-FSR the edges (1, 3) and (3, 6) are set to zero, with NR-OR-FSR only the edge (3, 6) is set to zero. The other estimation methods yield networks that contain the true set of edges as well as false positives, with the number of false positives varying from ten (Glasso-CV2, panel d; NR-AND-CV-1se, panel m; Ridge-CV, panel t) to one (SPACE-CV-1se, panel i).

**Figure 3:**
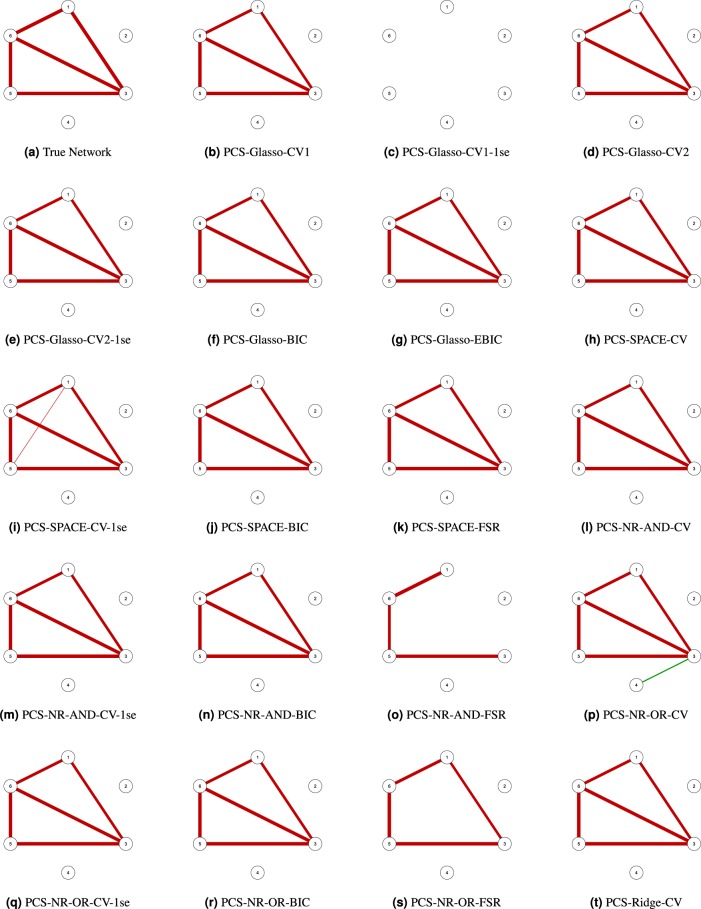
Estimated undirected networks for the toy example, after applying PCS.

Our PCS procedure aims to remove these false positive edges. The first step of the procedure is to apply one of the nineteen considered approaches. In the second step, we try to single out the false positives by thresholding the entries of the estimated partial correlation matrix. Specifically, only the partial correlations that are larger in absolute value than a given threshold are retained, whereas the others are set to zero and thus removed from the network. The threshold is calibrated by means of 10-fold cross-validation (see Methods section for more information). For the toy example the nineteen computed thresholds range from 0.0001 to 0.283. Figure 3 presents the networks that we obtain by applying these thresholds to the networks in Fig. 2. We observe that PCS-Glasso-CV1 (panel b), PCS-Glasso-CV2 (panel d), PCS-Glasso-CV2-1se (panel e), PCS-Glasso-BIC (panel f), PCS-Glasso-EBIC (panel g), PCS-SPACE-CV (panel h), PCS-SPACE-BIC (panel j), PCS-SPACE-FSR (panel k), PCS-NR-AND-CV (panel l), PCS-NR-AND-CV-1se (panel m), PCS-NR-AND-BIC (panel n), PCS-NR-OR-CV-1se (panel q), PCS-NR-AND-BIC (panel r) and Ridge-CV (panel t) remove the false positives and yield the true network. For, PCS-SPACE-CV-1se (panel i) none of the false positives are removed. PCS-NR-OR-CV (panel p) discards all but one false positive edge. Obviously, the networks with false negatives (panels c, o and s) cannot be improved by PCS.

### Simulation study with synthetic data

In this section we perform an extensive simulation study to evaluate and compare the performance of the different procedures. We will inspect the results obtained with the nineteen combinations used above and study whether they improve when adding PCS. To this end, we replicated the settings used by Liu *et al*.^22^, Ravikumar *et al*.^23^, Rothman *et al*.^24^ and Yuan and Lin^25^.

#### Design

**Figure 4:**
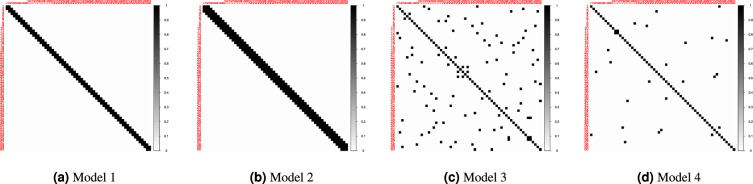
Heatmaps of the true simulated networks when *p* = 60. White represents partial correlations equal to zero, and black represents partial correlations different from zero.

Each simulated data set is generated by drawing *n* independent observations from a *p*-variate Gaussian distribution with mean zero and partial correlation matrix Γ. We considered two possible sample sizes *n* = {100, 500} and three different values of *p* = {20, 60, 200}. We inspected four different specifications of the population partial correlation matrix Γ. To illustrate these specifications for *p* = 60, we visualized them in Fig. 4.

1. Model 1: 2 neighbor Chain Graph, in which *ρ*_*ii*|*V*\{*i*}_ = 1 and *ρ*_*i*, *i*+1|*V*\{*i*, *i*+1}_ = *ρ*_*i*−1, *i*|*V*\{*i*, *i*−1}_ = −0.4, and all other edges are set to 0.
2. Model 2: 3 neighbor Chain Graph, in which *ρ*_*ii*|*V*\{*i*}_ = 1, *ρ*_*i*, *i*+1|*V*\{*i*, *i*+1}_ = *ρ*_*i*−1, *i*|*V*\{*i*, *i*−1}_ = −0.4, *ρ*_*i*, *i*+2|*V*\{*i*, *i*+2}_ = *ρ*_*i*−2, *i*|*V*\{*i*, *i*−2}_ = −0.2, and all other edges are set to 0.
3. Model 3: 2 nearest-neighbor graph. We first specify the inverse of the covariance matrix Σ as follows: we randomly select *p* points from a unit square and we compute all pairwise distances between the *p* points. Then, for each node the neighborhood set is found by including the two nodes with the smallest distance. Next, the OR-rule is applied to these neighborhood sets to derive the associated undirected network. The off-diagonal elements of the corresponding Σ^−1^ are randomly chosen from the interval $[\,-\,1,-\,0.5]\cup [0.5,1]$. To ensure that Σ^−1^ is positive definite, the matrix is transformed as: Σ^−1^ + (|*λ*(Σ^−1^)_min_| + 0.1)*I*_*p*_ where *λ*(Σ^−1^)_min_ refers to the smallest eigenvalue and *I*_*p*_ is an identity matrix of dimension *p*. To compute Γ we normalize Σ^−1^ and we multiply the off-diagonal elements by (−1).
4. Model 4: Random graph. We first specify Σ^−1^ as follows: each upper triangular element of Σ^−1^ is set equal to 0.3 with probability *ρ* and to zero otherwise. We set the probability *ρ* = {0.1, 0.01, 0.001} when *p* = {20, 60, 200}, respectively. Next, we set the lower triangular elements equal to the corresponding upper triangular elements. To ensure that Σ^−1^ is positive definite the matrix is transformed as in model 3. Finally, to compute Γ we normalize Σ^−1^ and we multiply the off-diagonal elements by (−1).

We generated 100 replicates for each cell of the design. An R script to conduct the simulation experiment is provided in the Supplementary Information.

#### Performance measures

To evaluate how well the different methods perform in distinguishing between true non-zero partial correlations and true zero ones, we compute the True Positive Rate (TPR) and False Positive Rate (FPR):

3 $${\rm{TPR}}=\frac{{\rm{TP}}}{{\rm{TP}}+{\rm{FN}}}$$

4 $${\rm{FPR}}=\frac{{\rm{FP}}}{{\rm{TN}}+{\rm{FP}}}$$

where TP is the number of true positives (true non-zero edges that are estimated as such), TN is the number of true negatives (true zero edges that are recognized as such), FP is the number of false positives (true zero edges that are estimated as non-zero) and FN is the number of false negatives (true non-zero edges that are estimated as zero). The TPR and FPR coefficients take values in the range [0, 1]. For the TPR a value of 0 indicates that the labeling of edges as non-zero is completely wrong, a value of 0.5 indicates that the procedure cannot do better than random prediction and a value of 1 indicates a perfect recovery of the non-zero edges. Similarly, a FPR value of 0 indicates a perfect recovery of the zero edges, a value of 0.5 indicates that the procedure cannot do better than random prediction and a value of 1 indicates that the labeling of edges as zero is completely wrong. We also report the average number of TP and FP values across the 100 replicates.

#### Results

Tables 1 to 3 show the average TPR and FPR scores for the different methods under consideration for the different choices of *p*. We also report the average number of TP and FP for the different methods in Tables 4 to 6. First, we compare the performance of the methods without conducting PCS. The TPR and FPR scores depend strongly on the model used to generate the data and the values of *n* and *p* (i.e., amount of available information). In general, when comparing the performance of the different methods in controlling the amount of false non-zero partial correlations, we observe that for every combination of *p* and *n*, SPACE and NR perform better than Glasso. The results for Glasso are affected by the penalty tuning approach: whereas using cross-validation tends to introduce a large number of false positives across all different conditions, applying EBIC yields many false negatives. For NR and SPACE, the results depend on *n* and *p* and the data generating model. For *n* = 100, the best overall though still rather bad performance for Models 1 and 4 is obtained with some of the NR-AND variants and for Models 2 and 3 with some of the SPACE variants. We also note that for Model 2 none of the state-of-the-art methods (excluding Ridge) is able to efficiently estimate the true number of positive edges. Furthermore, in the high-dimensional case (i.e., *p* > *n*) all approaches perform badly in controlling the amount of false positive edges. When *n* = 500, the TPR and FPR values are clearly better than for the low-sample size setting and indicate good overall performance.

**Table 1:** Average true positive rate (TPR) and false positive rate (FPR) over 100 replications when *p* = 20.

*n* = 100	Model 1				Model 2				Model 3				Model 4			
	TPR		FPR		TPR		FPR		TPR		FPR		TPR		FPR	
	no-PCS	PCS	no-PCS	PCS	no-PCS	PCS	no-PCS	PCS	no-PCS	PCS	no-PCS	PCS	no-PCS	PCS	no-PCS	PCS
Glasso-CV	0.999	0.989	0.160	0.008	0.781	0.647	0.240	0.100	0.963	0.901	0.083	0.009	0.989	0.860	0.174	0.014
Glasso-CV-1se	0.992	0.985	0.042	0.019	0.315	0.309	0.019	0.018	0.866	0.849	0.013	0.007	0.853	0.825	0.022	0.013
Glasso-CV2	1.000	0.988	0.316	0.007	0.932	0.653	0.547	0.047	0.993	0.923	0.275	0.003	0.990	0.857	0.200	0.014
Glasso-CV2-1se	0.999	0.988	0.138	0.011	0.794	0.650	0.261	0.095	0.971	0.908	0.107	0.008	0.924	0.858	0.051	0.017
Glasso-BIC	1.000	0.989	0.213	0.008	0.066	0.059	0.014	0.009	0.988	0.927	0.206	0.006	0.659	0.621	0.051	0.011
Glasso-EBIC	0.300	0.299	0.021	0.003	0.000	0.000	0.000	0.000	0.947	0.899	0.054	0.009	0.000	0.000	0.000	0.000
SPACE-CV	0.993	0.991	0.015	0.010	0.667	0.632	0.072	0.054	0.939	0.919	0.016	0.008	0.910	0.876	0.024	0.016
SPACE-CV-1se	0.991	0.989	0.007	0.005	0.550	0.541	0.034	0.032	0.913	0.887	0.005	0.003	0.875	0.833	0.011	0.009
SPACE-BIC	0.994	0.991	0.014	0.007	0.624	0.588	0.044	0.036	0.956	0.907	0.013	0.004	0.938	0.873	0.031	0.015
SPACE-FSR	0.997	0.992	0.026	0.009	0.599	0.571	0.045	0.036	0.964	0.924	0.025	0.007	0.961	0.869	0.044	0.016
NR-AND-CV	0.997	0.979	0.116	0.015	0.819	0.697	0.215	0.047	0.978	0.944	0.084	0.007	0.945	0.838	0.081	0.033
NR-AND-CV-1se	0.942	0.940	0.007	0.004	0.425	0.417	0.022	0.017	0.834	0.817	0.003	0.001	0.623	0.588	0.004	0.003
NR-AND-BIC	0.992	0.987	0.024	0.006	0.522	0.504	0.039	0.022	0.915	0.884	0.012	0.002	0.847	0.793	0.014	0.007
NR-AND-FSR	0.793	0.793	0.000	0.000	0.122	0.113	0.000	0.000	0.730	0.725	0.000	0.000	0.411	0.403	0.000	0.000
NR-OR-CV	1.000	0.967	0.296	0.023	0.906	0.682	0.445	0.041	0.991	0.941	0.267	0.014	0.982	0.749	0.238	0.025
NR-OR-CV-1se	0.988	0.987	0.027	0.013	0.617	0.595	0.078	0.045	0.917	0.887	0.021	0.003	0.838	0.733	0.027	0.008
NR-OR-BIC	0.996	0.979	0.078	0.009	0.709	0.633	0.122	0.033	0.962	0.921	0.061	0.004	0.920	0.782	0.054	0.014
NR-OR-FSR	0.867	0.865	0.001	0.001	0.124	0.116	0.000	0.000	0.767	0.759	0.000	0.000	0.527	0.524	0.001	0.001
Ridge-CV	1.000	0.867	1.000	0.143	1.000	0.572	1.000	0.242	1.000	0.828	1.000	0.046	1.000	0.222	1.000	0.047
***n*** = **500**	**no-PCS**	**PCS**	**no-PCS**	**PCS**	**no-PCS**	**PCS**	**no-PCS**	**PCS**	**no-PCS**	**PCS**	**no-PCS**	**PCS**	**no-PCS**	**PCS**	**no-PCS**	**PCS**
Glasso-CV	1.000	1.000	0.076	0.001	0.999	0.981	0.277	0.036	0.988	0.984	0.023	0.014	1.000	1.000	0.071	0.003
Glasso-CV-1se	1.000	1.000	0.034	0.002	0.947	0.942	0.090	0.080	0.943	0.943	0.017	0.012	1.000	1.000	0.016	0.003
Glasso-CV2	1.000	1.000	0.314	0.002	1.000	0.988	0.639	0.011	1.000	1.000	0.264	0.002	1.000	1.000	0.169	0.002
Glasso-CV2-1se	1.000	1.000	0.120	0.002	1.000	0.985	0.393	0.024	1.000	0.999	0.092	0.004	1.000	1.000	0.036	0.004
Glasso-BIC	1.000	1.000	0.233	0.002	0.999	0.983	0.318	0.030	1.000	1.000	0.261	0.001	1.000	1.000	0.113	0.002
Glasso-EBIC	1.000	1.000	0.149	0.002	0.993	0.974	0.170	0.062	1.000	0.999	0.096	0.003	1.000	1.000	0.060	0.002
SPACE-CV	1.000	1.000	0.008	0.002	0.989	0.985	0.041	0.029	1.000	1.000	0.008	0.002	1.000	1.000	0.012	0.001
SPACE-CV-1se	1.000	1.000	0.005	0.001	0.957	0.956	0.018	0.018	1.000	0.999	0.005	0.001	1.000	1.000	0.009	0.001
SPACE-BIC	1.000	1.000	0.007	0.002	0.983	0.978	0.021	0.019	1.000	0.999	0.009	0.001	1.000	1.000	0.026	0.001
SPACE-FSR	1.000	1.000	0.020	0.001	0.993	0.985	0.040	0.029	1.000	0.999	0.023	0.002	1.000	1.000	0.044	0.002
NR-AND-CV	1.000	1.000	0.111	0.003	1.000	0.991	0.273	0.012	1.000	1.000	0.081	0.002	1.000	1.000	0.079	0.001
NR-AND-CV-1se	1.000	1.000	0.001	0.000	0.931	0.930	0.018	0.004	0.998	0.998	0.000	0.000	0.995	0.994	0.001	0.000
NR-AND-BIC	1.000	1.000	0.009	0.001	0.992	0.989	0.055	0.007	0.999	0.999	0.007	0.001	1.000	1.000	0.008	0.001
NR-AND-FSR	1.000	1.000	0.000	0.000	0.612	0.612	0.002	0.002	0.988	0.988	0.000	0.000	1.000	1.000	0.000	0.000
NR-OR-CV	1.000	1.000	0.305	0.002	1.000	0.987	0.510	0.012	1.000	1.000	0.274	0.002	1.000	1.000	0.239	0.001
NR-OR-CV-1se	1.000	1.000	0.003	0.001	0.981	0.979	0.050	0.006	0.999	0.999	0.006	0.000	0.999	0.999	0.011	0.000
NR-OR-BIC	1.000	1.000	0.046	0.001	0.998	0.992	0.148	0.008	1.000	1.000	0.038	0.001	1.000	1.000	0.034	0.001
NR-OR-FSR	1.000	1.000	0.000	0.000	0.662	0.662	0.004	0.002	0.994	0.994	0.000	0.000	1.000	1.000	0.001	0.000
Ridge-CV	1.000	1.000	1.000	0.002	1.000	0.977	1.000	0.021	1.000	0.999	1.000	0.003	1.000	0.996	1.000	0.010

Note: Standard errors for TPR range from 0.002 to 0.046 and standard errors for FPR range from 0.000 to 0.019.

**Table 2:** Average true positive rate (TPR) and false positive rate (FPR) over 100 replications when *p* = 60.

*n* = 100	Model 1				Model 2				Model 3				Model 4			
	TPR		FPR		TPR		FPR		TPR		FPR		TPR		FPR	
	no-PCS	PCS	no-PCS	PCS	no-PCS	PCS	no-PCS	PCS	no-PCS	PCS	no-PCS	PCS	no-PCS	PCS	no-PCS	PCS
Glasso-CV	1.000	0.981	0.086	0.002	0.655	0.467	0.103	0.018	0.935	0.750	0.079	0.003	1.000	0.915	0.079	0.001
Glasso-CV-1se	0.998	0.977	0.028	0.003	0.369	0.351	0.010	0.008	0.817	0.714	0.014	0.003	0.982	0.908	0.008	0.001
Glasso-CV2	1.000	0.980	0.161	0.002	0.822	0.496	0.268	0.011	0.957	0.761	0.132	0.003	0.997	0.905	0.055	0.001
Glasso-CV2-1se	1.000	0.979	0.084	0.002	0.681	0.475	0.123	0.018	0.923	0.740	0.064	0.003	0.978	0.898	0.007	0.001
Glasso-BIC	0.999	0.980	0.051	0.003	0.000	0.000	0.000	0.000	0.722	0.621	0.015	0.003	0.029	0.028	0.000	0.000
Glasso-EBIC	0.000	0.000	0.000	0.000	0.000	0.000	0.000	0.000	0.000	0.000	0.000	0.000	0.000	0.000	0.000	0.000
SPACE-CV	0.993	0.989	0.004	0.003	0.569	0.531	0.029	0.019	0.834	0.795	0.009	0.005	0.982	0.943	0.004	0.001
SPACE-CV-1se	0.986	0.984	0.002	0.002	0.474	0.466	0.013	0.012	0.766	0.755	0.003	0.003	0.980	0.941	0.004	0.001
SPACE-BIC	0.997	0.989	0.010	0.003	0.573	0.520	0.025	0.016	0.885	0.809	0.014	0.005	0.997	0.939	0.022	0.001
SPACE-FSR	0.998	0.988	0.009	0.003	0.529	0.507	0.018	0.015	0.889	0.810	0.016	0.005	0.997	0.937	0.022	0.001
NR-AND-CV	0.998	0.972	0.048	0.006	0.687	0.563	0.088	0.013	0.893	0.793	0.047	0.004	0.988	0.882	0.027	0.001
NR-AND-CV-1se	0.940	0.938	0.003	0.002	0.314	0.311	0.007	0.007	0.566	0.537	0.002	0.001	0.733	0.689	0.001	0.000
NR-AND-BIC	0.986	0.974	0.008	0.002	0.348	0.332	0.007	0.004	0.728	0.685	0.005	0.001	0.949	0.902	0.003	0.000
NR-AND-FSR	0.670	0.670	0.000	0.000	0.044	0.038	0.000	0.000	0.308	0.307	0.000	0.000	0.589	0.586	0.000	0.000
NR-OR-CV	1.000	0.943	0.168	0.010	0.822	0.556	0.242	0.014	0.955	0.767	0.159	0.006	0.997	0.836	0.105	0.001
NR-OR-CV-1se	0.982	0.973	0.017	0.004	0.528	0.494	0.041	0.018	0.767	0.697	0.016	0.002	0.895	0.822	0.012	0.000
NR-OR-BIC	0.996	0.977	0.030	0.003	0.535	0.472	0.032	0.008	0.846	0.769	0.022	0.002	0.977	0.916	0.014	0.001
NR-OR-FSR	0.756	0.756	0.000	0.000	0.044	0.040	0.000	0.000	0.331	0.330	0.000	0.000	0.621	0.618	0.000	0.000
Ridge-CV	1.000	0.457	1.000	0.027	1.000	0.350	1.000	0.010	1.000	0.497	1.000	0.012	1.000	0.242	1.000	0.001
***n*** **= = 500**	**no-PCS**	**PCS**	**no-PCS**	**PCS**	**no-PCS**	**PCS**	**no-PCS**	**PCS**	**no-PCS**	**PCS**	**no-PCS**	**PCS**	**no-PCS**	**PCS**	**no-PCS**	**PCS**
Glasso-CV	1.000	1.000	0.029	0.000	0.996	0.949	0.136	0.013	0.999	0.992	0.024	0.000	1.000	1.000	0.043	0.000
Glasso-CV-1se	1.000	1.000	0.015	0.000	0.970	0.932	0.061	0.023	0.989	0.986	0.004	0.001	1.000	1.000	0.001	0.000
Glasso-CV2	1.000	1.000	0.151	0.000	1.000	0.974	0.383	0.004	1.000	0.997	0.206	0.000	1.000	1.000	0.059	0.000
Glasso-CV2-1se	1.000	1.000	0.076	0.000	1.000	0.964	0.274	0.007	1.000	0.996	0.071	0.000	1.000	1.000	0.004	0.000
Glasso-BIC	1.000	1.000	0.079	0.000	0.985	0.940	0.081	0.021	1.000	0.994	0.060	0.000	1.000	1.000	0.007	0.000
Glasso-EBIC	1.000	1.000	0.038	0.000	0.871	0.865	0.025	0.022	0.998	0.990	0.010	0.001	1.000	1.000	0.003	0.000
SPACE-CV	1.000	1.000	0.001	0.000	0.985	0.979	0.017	0.012	1.000	0.998	0.002	0.000	1.000	1.000	0.004	0.000
SPACE-CV-1se	1.000	1.000	0.001	0.000	0.947	0.947	0.007	0.007	1.000	0.998	0.002	0.000	1.000	1.000	0.004	0.000
SPACE-BIC	1.000	1.000	0.003	0.000	0.982	0.977	0.011	0.010	1.000	0.999	0.005	0.000	1.000	1.000	0.010	0.000
SPACE-FSR	1.000	1.000	0.006	0.000	0.983	0.978	0.015	0.012	1.000	0.999	0.014	0.000	1.000	1.000	0.023	0.000
NR-AND-CV	1.000	1.000	0.041	0.000	0.999	0.985	0.127	0.002	1.000	0.999	0.046	0.000	1.000	1.000	0.022	0.000
NR-AND-CV-1se	1.000	1.000	0.000	0.000	0.926	0.925	0.008	0.001	0.972	0.972	0.000	0.000	1.000	1.000	0.000	0.000
NR-AND-BIC	1.000	1.000	0.003	0.000	0.975	0.974	0.016	0.001	0.999	0.998	0.003	0.000	1.000	1.000	0.001	0.000
NR-AND-FSR	1.000	1.000	0.000	0.000	0.537	0.537	0.000	0.000	0.965	0.964	0.000	0.000	1.000	1.000	0.000	0.000
NR-OR-CV	1.000	1.000	0.162	0.000	1.000	0.979	0.297	0.003	1.000	1.000	0.157	0.000	1.000	1.000	0.094	0.000
NR-OR-CV-1se	1.000	1.000	0.001	0.000	0.974	0.970	0.026	0.001	0.995	0.995	0.002	0.000	1.000	1.000	0.005	0.000
NR-OR-BIC	1.000	1.000	0.016	0.000	0.991	0.986	0.053	0.001	1.000	0.999	0.015	0.000	1.000	1.000	0.008	0.000
NR-OR-FSR	1.000	1.000	0.000	0.000	0.563	0.563	0.001	0.000	0.985	0.985	0.000	0.000	1.000	1.000	0.000	0.000
Ridge-CV	1.000	1.000	1.000	0.002	1.000	0.890	1.000	0.024	1.000	0.866	1.000	0.015	1.000	0.761	1.000	0.011

Note: Standard errors for TPR range from 0.000 to 0.029 and standard errors for FPR range from 0.000 to 0.010.

**Table 3:** Average true positive rate (TPR) and false positive rate (FPR) over 100 replications when *p* = 200.

*n* = 100	Model 1				Model 2				Model 3				Model 4			
	TPR		FPR		TPR		FPR		TPR		FPR		TPR		FPR	
	no-PCS	PCS	no-PCS	PCS	no-PCS	PCS	no-PCS	PCS	no-PCS	PCS	no-PCS	PCS	no-PCS	PCS	no-PCS	PCS
Glasso-CV	0.999	0.968	0.039	0.001	0.515	0.273	0.033	0.002	0.920	0.723	0.033	0.000	0.990	0.670	0.033	0.000
Glasso-CV-1se	0.999	0.965	0.018	0.001	0.321	0.259	0.005	0.002	0.873	0.720	0.011	0.001	0.956	0.647	0.006	0.000
Glasso-CV2	1.000	0.969	0.065	0.000	0.611	0.283	0.071	0.002	0.932	0.733	0.044	0.000	0.974	0.664	0.011	0.000
Glasso-CV2-1se	0.999	0.967	0.041	0.001	0.517	0.272	0.034	0.002	0.912	0.725	0.028	0.000	0.801	0.646	0.001	0.000
Glasso-BIC	0.996	0.963	0.010	0.001	0.000	0.000	0.000	0.000	0.604	0.520	0.003	0.000	0.000	0.000	0.000	0.000
Glasso-EBIC	0.000	0.000	0.000	0.000	0.000	0.000	0.000	0.000	0.000	0.000	0.000	0.000	0.000	0.000	0.000	0.000
SPACE-CV	0.987	0.984	0.001	0.001	0.405	0.365	0.006	0.004	0.809	0.785	0.002	0.001	0.930	0.751	0.002	0.000
SPACE-CV-1se	0.978	0.977	0.001	0.001	0.332	0.327	0.003	0.003	0.801	0.780	0.001	0.001	0.930	0.759	0.002	0.000
SPACE-BIC	0.997	0.981	0.006	0.001	0.497	0.365	0.014	0.003	0.908	0.787	0.010	0.001	0.985	0.718	0.016	0.000
SPACE-FSR	0.994	0.983	0.003	0.001	0.443	0.365	0.009	0.003	0.887	0.786	0.007	0.001	0.978	0.733	0.011	0.000
NR-AND-CV	0.995	0.952	0.018	0.002	0.511	0.358	0.029	0.004	0.874	0.749	0.016	0.001	0.944	0.542	0.010	0.000
NR-AND-CV-1se	0.910	0.908	0.001	0.000	0.187	0.170	0.002	0.002	0.615	0.585	0.001	0.000	0.524	0.390	0.001	0.000
NR-AND-BIC	0.973	0.965	0.002	0.001	0.238	0.217	0.002	0.001	0.750	0.706	0.001	0.000	0.839	0.637	0.001	0.000
NR-AND-FSR	0.475	0.475	0.000	0.000	0.010	0.008	0.000	0.000	0.344	0.343	0.000	0.000	0.239	0.237	0.000	0.000
NR-OR-CV	0.999	0.844	0.083	0.009	0.691	0.215	0.107	0.003	0.940	0.622	0.074	0.003	0.979	0.297	0.051	0.000
NR-OR-CV-1se	0.970	0.954	0.008	0.002	0.386	0.338	0.016	0.007	0.787	0.677	0.007	0.000	0.814	0.518	0.006	0.000
NR-OR-BIC	0.990	0.973	0.010	0.002	0.380	0.289	0.009	0.002	0.836	0.762	0.007	0.001	0.913	0.637	0.008	0.000
NR-OR-FSR	0.551	0.551	0.000	0.000	0.010	0.008	0.000	0.000	0.389	0.389	0.000	0.000	0.252	0.249	0.000	0.000
Ridge-CV	1.000	0.961	1.000	0.002	1.000	0.273	1.000	0.002	1.000	0.718	1.000	0.001	1.000	0.629	1.000	0.000
***n*** = **500**	**no-PCS**	**PCS**	**no-PCS**	**PCS**	**no-PCS**	**PCS**	**no-PCS**	**PCS**	**no-PCS**	**PCS**	**no-PCS**	**PCS**	**no-PCS**	**PCS**	**no-PCS**	**PCS**
Glasso-CV	1.000	1.000	0.012	0.000	0.986	0.886	0.064	0.004	0.989	0.975	0.006	0.001	1.000	1.000	0.023	0.000
Glasso-CV-1se	1.000	1.000	0.006	0.000	0.962	0.863	0.036	0.005	0.986	0.974	0.004	0.001	1.000	1.000	0.002	0.000
Glasso-CV2	1.000	1.000	0.080	0.000	0.999	0.928	0.218	0.002	0.999	0.985	0.041	0.000	1.000	1.000	0.011	0.000
Glasso-CV2-1se	1.000	1.000	0.042	0.000	0.996	0.908	0.125	0.003	0.999	0.986	0.041	0.000	1.000	1.000	0.001	0.000
Glasso-BIC	1.000	1.000	0.022	0.000	0.906	0.847	0.017	0.007	0.992	0.977	0.008	0.000	1.000	1.000	0.001	0.000
Glasso-EBIC	1.000	1.000	0.008	0.000	0.665	0.664	0.003	0.003	0.987	0.974	0.005	0.001	1.000	1.000	0.000	0.000
SPACE-CV	1.000	1.000	0.000	0.000	0.966	0.957	0.006	0.005	0.999	0.995	0.001	0.000	1.000	1.000	0.002	0.000
SPACE-CV-1se	1.000	1.000	0.000	0.000	0.931	0.931	0.003	0.003	0.999	0.994	0.001	0.000	1.000	1.000	0.002	0.000
SPACE-BIC	1.000	1.000	0.003	0.002	0.973	0.957	0.007	0.004	1.000	0.996	0.004	0.000	1.000	1.000	0.010	0.000
SPACE-FSR	1.000	1.000	0.002	0.001	0.963	0.958	0.006	0.005	1.000	0.996	0.006	0.000	1.000	1.000	0.013	0.000
NR-AND-CV	1.000	1.000	0.013	0.000	0.995	0.977	0.049	0.001	1.000	1.000	0.014	0.000	1.000	1.000	0.008	0.000
NR-AND-CV-1se	1.000	1.000	0.000	0.000	0.873	0.872	0.002	0.000	0.968	0.968	0.000	0.000	0.999	0.999	0.000	0.000
NR-AND-BIC	1.000	1.000	0.001	0.000	0.934	0.932	0.004	0.000	0.998	0.997	0.001	0.000	1.000	1.000	0.001	0.000
NR-AND-FSR	1.000	1.000	0.000	0.000	0.506	0.506	0.000	0.000	0.942	0.942	0.000	0.000	1.000	1.000	0.000	0.000
NR-OR-CV	1.000	1.000	0.073	0.000	0.999	0.962	0.143	0.001	1.000	0.999	0.067	0.000	1.000	1.000	0.037	0.000
NR-OR-CV-1se	1.000	1.000	0.001	0.000	0.944	0.939	0.010	0.000	0.991	0.990	0.001	0.000	1.000	1.000	0.003	0.000
NR-OR-BIC	1.000	1.000	0.005	0.000	0.972	0.968	0.015	0.000	0.999	0.999	0.004	0.000	1.000	1.000	0.004	0.000
NR-OR-FSR	1.000	1.000	0.000	0.000	0.516	0.516	0.000	0.000	0.960	0.960	0.000	0.000	1.000	1.000	0.000	0.000
Ridge-CV	1.000	1.000	1.000	0.000	1.000	0.742	1.000	0.008	1.000	0.965	1.000	0.003	1.000	1.000	1.000	0.000

Note: Standard errors for TPR range from 0.000 to 0.037 and standard errors for FPR range from 0.000 to 0.002.

**Table 4:** Average number of true positive edges (TP) and false positive edges (FP) over 100 replications when *p* = 20. For each model the number of non-zero partial correlations are: 19 for Model 1, 37 for Model 2, 16 for Model 3 and 12 for Model 4.

*n* = 100	Model 1				Model 2				Model 3				Model 4			
	TP		FP		TP		FP		TP		FP		TP		FP	
	no-PCS	PCS	no-PCS	PCS	no-PCS	PCS	no-PCS	PCS	no-PCS	PCS	no-PCS	PCS	no-PCS	PCS	no-PCS	PCS
Glasso-CV	18.99	18.80	27.28	1.45	28.91	23.93	36.65	15.30	15.41	14.41	14.38	1.49	11.87	10.32	30.96	2.49
Glasso-CV-1se	18.85	18.71	7.13	3.21	11.64	11.42	2.87	2.70	13.85	13.58	2.22	1.29	10.24	9.90	3.98	2.36
Glasso-CV2	19.00	18.78	53.98	1.24	34.49	24.15	83.68	7.22	15.89	14.77	47.83	0.53	11.88	10.28	35.57	2.53
Glasso-CV2-1se	18.98	18.78	23.59	1.89	29.39	24.04	39.98	14.49	15.53	14.53	18.61	1.38	11.09	10.29	9.06	3.05
Glasso-BIC	19.00	18.79	36.45	1.31	2.44	2.19	2.09	1.33	15.81	14.83	35.93	0.97	7.91	7.45	9.14	1.97
Glasso-EBIC	5.70	5.68	3.62	0.56	0.00	0.00	0.00	0.00	15.15	14.39	9.48	1.60	0.00	0.00	0.00	0.00
SPACE-CV	18.86	18.83	2.62	1.77	24.68	23.39	10.96	8.22	15.02	14.71	2.81	1.34	10.92	10.51	4.20	2.79
SPACE-CV-1se	18.83	18.79	1.18	0.93	20.34	20.00	5.22	4.85	14.61	14.19	0.86	0.56	10.50	10.00	2.01	1.54
SPACE-BIC	18.88	18.82	2.36	1.22	23.07	21.75	6.72	5.49	15.29	14.51	2.21	0.72	11.25	10.48	5.50	2.65
SPACE-FSR	18.94	18.84	4.39	1.49	22.18	21.11	6.93	5.54	15.43	14.78	4.32	1.27	11.53	10.43	7.85	2.93
NR-AND-CV	18.94	18.61	19.87	2.63	30.31	25.80	32.95	7.21	15.65	15.11	14.59	1.26	11.34	10.05	14.41	5.90
NR-AND-CV-1se	17.89	17.86	1.21	0.71	15.73	15.43	3.29	2.54	13.35	13.07	0.54	0.26	7.47	7.05	0.75	0.50
NR-AND-BIC	18.84	18.75	4.09	1.03	19.32	18.66	5.92	3.32	14.64	14.14	2.15	0.27	10.16	9.51	2.44	1.30
NR-AND-FSR	15.06	15.06	0.06	0.04	4.52	4.17	0.02	0.02	11.68	11.60	0.03	0.03	4.93	4.84	0.05	0.05
NR-OR-CV	19.00	18.37	50.63	3.85	33.54	25.22	68.12	6.27	15.86	15.06	46.42	2.42	11.78	8.99	42.36	4.45
NR-OR-CV-1se	18.77	18.75	4.55	2.22	22.83	22.00	11.87	6.90	14.67	14.19	3.61	0.54	10.05	8.80	4.84	1.34
NR-OR-BIC	18.92	18.60	13.38	1.57	26.24	23.43	18.69	5.05	15.39	14.73	10.57	0.66	11.04	9.38	9.70	2.50
NR-OR-FSR	16.48	16.44	0.13	0.09	4.59	4.31	0.03	0.02	12.27	12.14	0.05	0.05	6.32	6.29	0.10	0.10
Ridge-CV	19.00	16.48	171.00	24.42	37.00	21.16	153.00	36.96	16.00	13.24	174.00	8.02	12.00	2.66	178.00	8.40
***n*** = **500**	**no-PCS**	**PCS**	**no-PCS**	**PCS**	**no-PCS**	**PCS**	**no-PCS**	**PCS**	**no-PCS**	**PCS**	**no-PCS**	**PCS**	**no-PCS**	**PCS**	**no-PCS**	**PCS**
Glasso-CV	19.00	19.00	12.99	0.16	36.97	36.29	42.31	5.46	15.81	15.74	4.03	2.36	12.00	12.00	12.64	0.47
Glasso-CV-1se	19.00	19.00	5.82	0.34	35.04	34.85	13.74	12.29	15.08	15.08	2.90	2.12	12.00	12.00	2.79	0.56
Glasso-CV2	19.00	19.00	53.73	0.26	37.00	36.55	97.70	1.72	16.00	16.00	45.86	0.39	12.00	12.00	30.01	0.43
Glasso-CV2-1se	19.00	19.00	20.54	0.34	36.99	36.46	60.17	3.69	16.00	15.99	16.05	0.61	12.00	12.00	6.39	0.63
Glasso-BIC	19.00	19.00	39.90	0.35	36.98	36.36	48.67	4.63	16.00	16.00	45.40	0.22	12.00	12.00	20.10	0.41
Glasso-EBIC	19.00	19.00	25.45	0.37	36.74	36.02	26.08	9.54	16.00	15.99	16.65	0.50	12.00	12.00	10.61	0.40
SPACE-CV	19.00	19.00	1.35	0.31	36.60	36.43	6.25	4.38	16.00	16.00	1.44	0.35	12.00	12.00	2.16	0.23
SPACE-CV-1se	19.00	19.00	0.78	0.18	35.41	35.37	2.70	2.69	16.00	15.99	0.84	0.23	12.00	12.00	1.66	0.17
SPACE-BIC	19.00	19.00	1.26	0.26	36.36	36.20	3.24	2.98	16.00	15.99	1.49	0.23	12.00	12.00	4.62	0.17
SPACE-FSR	19.00	19.00	3.42	0.25	36.73	36.46	6.09	4.39	16.00	15.99	3.93	0.41	12.00	12.00	7.83	0.39
NR-AND-CV	19.00	19.00	19.00	0.46	36.99	36.68	41.78	1.76	16.00	16.00	14.18	0.41	12.00	12.00	13.99	0.26
NR-AND-CV-1se	19.00	19.00	0.10	0.05	34.43	34.40	2.69	0.62	15.96	15.96	0.05	0.02	11.94	11.93	0.10	0.01
NR-AND-BIC	19.00	19.00	1.62	0.21	36.70	36.61	8.40	1.12	15.99	15.99	1.27	0.16	12.00	12.00	1.49	0.12
NR-AND-FSR	19.00	19.00	0.02	0.01	22.65	22.65	0.30	0.28	15.81	15.81	0.01	0.01	12.00	12.00	0.05	0.03
NR-OR-CV	19.00	19.00	52.20	0.26	36.99	36.51	78.02	1.89	16.00	16.00	47.73	0.36	12.00	12.00	42.47	0.09
NR-OR-CV-1se	19.00	19.00	0.54	0.16	36.29	36.23	7.68	0.96	15.98	15.98	0.96	0.07	11.99	11.99	1.96	0.06
NR-OR-BIC	19.00	19.00	7.88	0.19	36.94	36.72	22.68	1.18	16.00	16.00	6.59	0.16	12.00	12.00	6.09	0.17
NR-OR-FSR	19.00	19.00	0.07	0.03	24.48	24.48	0.56	0.36	15.90	15.90	0.08	0.05	12.00	12.00	0.09	0.07
Ridge-CV	19.00	19.00	171.00	0.27	37.00	36.15	153.00	3.19	16.00	15.99	174.00	0.57	12.00	11.95	178.00	1.75

For each model the number of non-zero partial correlations are: 19 for Model 1, 37 for Model 2, 16 for Model 3 and 12 for Model 4.

Note: Standard errors for TP range from 0.060 to 0.875 and standard errors for FP range from 0.024 to 2.913.

**Table 5:** Average number of true positive edges (TP) and false positive edges (FP) over 100 replications when *p* = 60. For each model the number of non-zero partial correlations are: 59 for Model 1, 117 for Model 2, 48 for Model 3 and 13 for Model 4.

*n* = 100	Model 1				Model 2				Model 3				Model 4			
	TP		FP		TP		FP		TP		FP		TP		FP	
	no-PCS	PCS	no-PCS	PCS	no-PCS	PCS	no-PCS	PCS	no-PCS	PCS	no-PCS	PCS	no-PCS	PCS	no-PCS	PCS
Glasso-CV	58.99	57.86	146.82	3.62	76.66	54.68	170.65	29.75	44.87	36.01	136.18	5.62	13.00	11.90	139.32	1.40
Glasso-CV-1se	58.90	57.65	47.81	5.26	43.23	41.01	17.20	13.26	39.20	34.25	24.58	5.00	12.77	11.81	14.39	1.05
Glasso-CV2	59.00	57.81	275.50	3.05	96.22	58.05	443.62	18.63	45.95	36.53	227.62	5.43	12.96	11.77	96.47	0.98
Glasso-CV2-1se	58.99	57.77	143.88	3.58	79.65	55.61	203.21	29.94	44.30	35.54	111.01	5.61	12.72	11.67	12.69	1.22
Glasso-BIC	58.97	57.82	87.72	4.57	0.00	0.00	0.00	0.00	34.67	29.82	25.59	4.33	0.38	0.36	0.30	0.01
Glasso-EBIC	0.00	0.00	0.00	0.00	0.00	0.00	0.00	0.00	0.00	0.00	0.00	0.00	0.00	0.00	0.00	0.00
SPACE-CV	58.57	58.33	7.62	5.16	66.57	62.07	47.32	30.92	40.04	38.15	14.94	8.19	12.77	12.26	7.08	2.01
SPACE-CV-1se	58.16	58.04	3.39	3.11	55.44	54.48	22.12	19.92	36.75	36.25	5.52	4.73	12.74	12.23	6.26	2.16
SPACE-BIC	58.84	58.34	17.20	5.05	67.09	60.84	41.09	26.46	42.46	38.83	24.87	8.61	12.96	12.21	39.09	2.01
SPACE-FSR	58.89	58.28	15.29	4.79	61.88	59.36	30.51	24.70	42.69	38.90	28.35	8.59	12.96	12.18	38.13	2.15
NR-AND-CV	58.90	57.36	82.44	9.60	80.37	65.83	146.24	20.96	42.85	38.08	80.62	7.65	12.85	11.46	46.63	1.66
NR-AND-CV-1se	55.44	55.35	4.94	2.73	36.76	36.34	12.38	10.80	27.19	25.76	3.75	1.41	9.53	8.96	2.63	0.14
NR-AND-BIC	58.16	57.49	12.96	2.98	40.67	38.79	11.08	6.13	34.95	32.89	8.18	2.00	12.34	11.72	5.83	0.87
NR-AND-FSR	39.53	39.51	0.04	0.04	5.09	4.48	0.05	0.03	14.79	14.73	0.02	0.02	7.66	7.62	0.01	0.01
NR-OR-CV	59.00	55.62	287.18	16.60	96.20	65.04	399.67	22.58	45.83	36.83	274.38	10.12	12.96	10.87	184.09	2.44
NR-OR-CV-1se	57.95	57.38	29.18	6.82	61.76	57.75	67.10	30.30	36.81	33.47	27.38	3.39	11.63	10.68	20.23	0.53
NR-OR-BIC	58.76	57.63	51.54	5.84	62.59	55.23	52.46	12.70	40.63	36.92	37.44	4.05	12.70	11.91	25.02	1.53
NR-OR-FSR	44.60	44.59	0.11	0.11	5.12	4.63	0.05	0.04	15.91	15.84	0.05	0.05	8.07	8.04	0.01	0.01
Ridge-CV	59.00	26.94	1711.00	45.40	117.00	41.00	1653.00	17.02	48.00	23.85	1722.00	20.62	13.00	3.14	1757.00	1.60
***n*** = **500**	**no-PCS**	**PCS**	**no-PCS**	**PCS**	**no-PCS**	**PCS**	**no-PCS**	**PCS**	**no-PCS**	**PCS**	**no-PCS**	**PCS**	**no-PCS**	**PCS**	**no-PCS**	**PCS**
Glasso-CV	59.00	59.00	2.55	0.13	115.23	114.57	28.81	20.54	47.99	47.91	4.01	0.57	13.00	13.00	6.95	0.13
Glasso-CV-1se	59.00	59.00	2.14	0.19	110.79	110.77	11.24	11.18	47.99	47.92	3.41	0.73	13.00	13.00	6.51	0.10
Glasso-CV2	59.00	59.00	9.88	0.16	115.05	114.48	24.63	20.13	47.99	47.93	23.79	0.35	13.00	13.00	40.12	0.16
Glasso-CV2-1se	59.00	59.00	5.41	0.15	114.92	114.34	18.53	16.74	47.99	47.93	8.93	0.66	13.00	13.00	17.85	0.15
Glasso-BIC	59.00	59.00	48.92	0.24	116.56	111.02	225.04	22.06	47.94	47.63	40.98	0.78	13.00	13.00	76.38	0.08
Glasso-EBIC	59.00	59.00	25.07	0.39	113.45	109.01	101.00	38.68	47.46	47.31	6.31	2.08	13.00	13.00	2.62	0.19
SPACE-CV	59.00	59.00	135.88	0.19	115.28	110.00	133.71	34.43	47.99	47.73	102.61	0.44	13.00	13.00	11.46	0.13
SPACE-CV-1se	59.00	59.00	64.47	0.17	101.85	101.19	41.04	35.93	47.91	47.51	16.72	1.12	13.00	13.00	5.45	0.13
SPACE-BIC	59.00	59.00	258.24	0.12	116.99	113.90	633.67	6.79	48.00	47.84	353.98	0.26	13.00	13.00	104.50	0.09
SPACE-FSR	59.00	59.00	130.22	0.15	116.96	112.82	453.52	11.60	47.99	47.79	122.34	0.34	13.00	13.00	7.68	0.10
NR-AND-CV	59.00	59.00	69.95	0.11	116.91	115.28	209.91	3.73	47.99	47.97	79.27	0.23	13.00	13.00	39.34	0.23
NR-AND-CV-1se	59.00	59.00	0.19	0.11	108.39	108.27	12.52	1.27	46.67	46.65	0.25	0.05	13.00	13.00	0.53	0.01
NR-AND-BIC	59.00	59.00	0.02	0.01	62.85	62.85	0.35	0.35	46.30	46.29	0.02	0.02	13.00	13.00	0.01	0.00
NR-AND-FSR	59.00	59.00	4.30	0.05	114.07	113.92	27.23	1.66	47.93	47.91	4.69	0.12	13.00	13.00	1.98	0.10
NR-OR-CV	59.00	59.00	277.54	0.08	116.98	114.55	491.73	4.79	48.00	47.98	270.86	0.32	13.00	13.00	164.58	0.18
NR-OR-CV-1se	59.00	59.00	2.38	0.31	113.92	113.50	43.28	1.58	47.78	47.75	4.18	0.17	13.00	13.00	9.11	0.01
NR-OR-BIC	59.00	59.00	0.05	0.03	65.82	65.82	0.88	0.79	47.27	47.27	0.07	0.06	13.00	13.00	0.03	0.01
NR-OR-FSR	59.00	59.00	26.60	0.09	115.96	115.33	87.34	2.24	47.99	47.97	25.56	0.15	13.00	13.00	14.55	0.08
Ridge-CV	59.00	59.00	1711.00	3.83	117.00	104.13	1653.00	39.56	48.00	41.55	1722.00	25.85	13.00	9.89	1757.00	19.18

Note: Standard errors for TP range from 0.000 to 1.385 and standard errors for FP range from 0.000 to 17.702.

**Table 6:** Average number of true positive edges (TP) and false positive edges (FP) over 100 replications when *p* = 200. For each model the number of non-zero partial correlations are: 199 for Model 1, 397 for Model 2, 142 for Model 3 and 23 for Model 4.

*n* = 100	Model 1				Model 2				Model 3				Model 4			
	TP		FP		TP		FP		TP		FP		TP		FP	
	no-PCS	PCS	no-PCS	PCS	no-PCS	PCS	no-PCS	PCS	no-PCS	PCS	no-PCS	PCS	no-PCS	PCS	no-PCS	PCS
Glasso-CV	198.86	192.63	758.85	11.33	204.29	108.27	637.13	38.70	130.62	102.64	654.20	7.87	22.78	15.40	664.69	1.81
Glasso-CV-1se	198.72	192.02	351.17	13.31	127.57	102.89	91.35	34.79	123.99	102.23	217.95	10.21	21.98	14.87	121.33	1.36
Glasso-CV2	198.94	192.82	1283.12	9.31	242.43	112.48	1385.28	38.17	132.29	104.02	870.41	8.65	22.41	15.27	220.99	1.44
Glasso-CV2-1se	198.85	192.49	804.26	10.45	205.13	108.18	660.23	39.02	129.54	102.98	544.21	8.53	18.43	14.85	13.05	1.54
Glasso-BIC	198.30	191.62	198.60	14.85	0.00	0.00	0.00	0.00	85.78	73.89	58.30	7.97	0.00	0.00	0.00	0.00
Glasso-EBIC	0.00	0.00	0.00	0.00	0.00	0.00	0.00	0.00	0.00	0.00	0.00	0.00	0.00	0.00	0.00	0.00
SPACE-CV	196.49	195.78	27.26	19.45	160.83	144.91	126.05	73.34	114.93	111.52	29.78	19.71	21.40	17.27	41.19	4.33
SPACE-CV-1se	194.60	194.52	13.02	12.77	131.71	129.85	54.54	51.91	113.74	110.70	24.63	17.89	21.38	17.45	40.76	4.81
SPACE-BIC	198.44	195.26	120.78	14.80	197.37	144.83	265.74	58.57	128.87	111.73	203.32	16.20	22.65	16.52	323.32	3.62
SPACE-FSR	197.85	195.65	59.92	16.99	175.84	145.03	168.31	68.03	125.91	111.68	131.09	16.30	22.49	16.85	222.86	3.64
NR-AND-CV	198.08	189.54	360.86	42.59	202.81	142.22	571.98	80.00	124.14	106.40	306.85	20.49	21.72	12.46	192.20	1.70
NR-AND-CV-1se	181.12	180.78	17.53	8.63	74.24	67.35	37.06	29.53	87.31	83.08	11.94	3.82	12.06	8.98	12.03	0.14
NR-AND-BIC	193.65	192.08	40.27	10.54	94.41	86.01	30.52	17.18	106.49	100.25	24.06	6.20	19.29	14.64	19.94	1.70
NR-AND-FSR	94.51	94.45	0.04	0.04	4.09	3.35	0.00	0.00	48.82	48.77	0.03	0.03	5.50	5.46	0.00	0.00
NR-OR-CV	198.73	167.88	1634.23	176.68	274.34	85.16	2094.31	56.90	133.41	88.36	1459.24	53.96	22.51	6.84	1012.65	1.67
NR-OR-CV-1se	193.09	189.88	167.15	29.94	153.05	134.04	303.67	137.51	111.80	96.10	146.74	8.11	18.72	11.91	112.39	1.12
NR-OR-BIC	196.96	193.57	190.08	33.21	150.86	114.86	178.67	35.82	118.67	108.24	131.56	18.48	20.99	14.65	162.86	2.96
NR-OR-FSR	109.58	109.55	0.14	0.13	4.09	3.37	0.00	0.00	55.29	55.18	0.07	0.07	5.80	5.72	0.00	0.00
Ridge-CV	199.00	191.26	19701.00	30.04	397.00	108.21	19503.00	29.29	142.00	102.00	19758.00	18.46	23.00	14.47	19877.00	1.31
***n*** = **500**	**no-PCS**	**PCS**	**no-PCS**	**PCS**	**no-PCS**	**PCS**	**no-PCS**	**PCS**	**no-PCS**	**PCS**	**no-PCS**	**PCS**	**no-PCS**	**PCS**	**no-PCS**	**PCS**
Glasso-CV	199.00	199.00	235.07	0.15	391.50	351.69	1245.58	85.60	140.48	138.47	110.08	10.39	23.00	23.00	453.33	0.05
Glasso-CV-1se	199.00	199.00	116.99	0.25	381.73	342.47	696.92	101.95	140.06	138.34	87.60	12.44	23.00	23.00	39.53	0.06
Glasso-CV2	199.00	199.00	1582.27	0.20	396.71	368.39	4256.19	33.63	141.92	139.90	811.90	2.83	23.00	23.00	223.51	0.05
Glasso-CV2-1se	199.00	199.00	836.46	0.12	395.60	360.63	2440.77	60.64	141.92	139.95	811.90	3.10	23.00	23.00	10.84	0.11
Glasso-BIC	199.00	199.00	435.99	0.21	359.84	336.22	332.29	129.97	140.88	138.74	159.47	8.22	23.00	23.00	10.62	0.09
Glasso-EBIC	199.00	199.00	163.43	0.28	263.95	263.73	52.35	52.17	140.12	138.30	91.93	12.09	23.00	23.00	5.01	0.10
SPACE-CV	199.00	199.00	6.18	0.18	383.35	379.74	120.06	88.04	141.79	141.24	18.01	2.79	23.00	23.00	45.25	0.06
SPACE-CV-1se	199.00	199.00	5.67	0.17	369.73	369.66	59.44	59.05	141.79	141.18	17.56	2.72	23.00	23.00	45.11	0.05
SPACE-BIC	199.00	199.00	58.63	41.28	386.42	379.77	133.83	76.95	141.97	141.42	86.76	1.73	23.00	23.00	201.15	0.12
SPACE-FSR	199.00	199.00	31.80	24.48	382.41	380.19	108.06	93.11	141.98	141.47	110.12	1.32	23.00	23.00	251.95	0.05
NR-AND-CV	199.00	199.00	265.57	0.14	395.08	388.00	952.77	14.26	141.99	141.93	274.47	0.30	23.00	23.00	161.21	0.08
NR-AND-CV-1se	199.00	199.00	0.70	0.25	346.71	346.30	46.21	3.14	137.52	137.47	0.93	0.07	22.97	22.97	6.53	0.00
NR-AND-BIC	199.00	199.00	11.90	0.05	370.60	369.99	81.03	4.87	141.65	141.61	12.19	0.27	23.00	23.00	11.03	0.05
NR-AND-FSR	199.00	199.00	0.01	0.01	200.90	200.90	0.45	0.45	133.82	133.82	0.02	0.02	23.00	23.00	0.01	0.01
NR-OR-CV	199.00	199.00	1441.19	0.05	396.47	381.86	2795.27	23.46	142.00	141.81	1319.48	0.50	23.00	23.00	729.26	0.06
NR-OR-CV-1se	199.00	199.00	12.61	0.38	374.86	372.88	197.90	4.76	140.73	140.65	21.39	0.09	22.99	22.99	60.72	0.00
NR-OR-BIC	199.00	199.00	91.27	0.06	385.83	384.12	299.27	9.28	141.88	141.82	81.60	0.19	23.00	23.00	71.15	0.10
NR-OR-FSR	199.00	199.00	0.07	0.03	205.02	205.02	0.99	0.97	136.27	136.27	0.16	0.04	23.00	23.00	0.01	0.01
Ridge-CV	199.00	199.00	19701.00	0.69	397.00	294.45	19503.00	146.53	142.00	136.96	19758.00	54.15	23.00	23.00	19877.00	0.82

Note: Standard errors for TP range from 0.000 to 5.256 and standard errors for FP range from 0.000 to 41.561.

Turning to the results after applying PCS, we observe in Tables 1 to 6 that PCS-SPACE and PCS-NR estimate networks that contain a smaller number of false positive edges than the state-of-the-art methods without PCS. This improvement is larger for Models 1, 3 and 4 and when *n* = 100 and *n* < *p*, in that PCS is able to control the number of false positive edges without compromising the number of correctly estimated true edges. Furthermore, the performance differences between the different SPACE and NR variants have diminished. PCS-SPACE-BIC has the best overall performance across all the *n* = 100 conditions. For PCS-Glasso-EBIC the results cannot be improved by PCS, because Glasso-EBIC yields networks with a large number of false negatives. When *n* = 500, PCS performs almost perfectly in finding the non-zero edges in Models 1, 3 and 4, while for Model 2, the best overall performance is obtained with PCS-NR-OR-BIC when *p* = 20, 60 and with PCS-NR-OR-FSR when *p* = 200.

Finally, we study how the sample size and the non-sparsity level influence the height of the estimated threshold in the PCS procedure. For Glasso, SPACE, NR using the AND rule and NR using the OR rule, we estimate a linear mixed effect model with a random intercept in which observations are clustered according to the tuning procedure (i.e., different CV variants, information criteria or finite sample results). The model includes the estimated thresholds as the dependent variable and the sample size and the non-sparsity level as predictors. The non-sparsity level is computed as the number of true non-zero partial correlations divided by the total number of edges in the network. For Ridge we estimate the same model using OLS regression. Table 7 shows the obtained regression coefficients for each estimation procedure. We observe that across the different estimation procedures there is a significant negative relation between the sample size and the estimated threshold value. Also, we found a significant negative relation between the non-sparsity level and the threshold value parameter for all methods except Glasso.

**Table 7:** Regression coefficients, standard errors (SE), associated Wald’s *t*-scores and p-values for all predictors in the analysis.

	Predictors	Estimate	SE	*t*	p-value
Glasso	Intercept	0.06495	0.010	6.464	0.001
	Non-sparsity	0.00006	0.000	1.130	0.259
	*n*	−0.00002	0.000	-13.556	0.000
SPACE	Intercept	0.04268	0.006	6.961	0.006
	Non-sparsity	−0.00202	0.000	−39.938	0.000
	*n*	−0.00001	0.000	−4.455	0.000
NR-AND	Intercept	0.22450	0.021	10.460	0.002
	Non-sparsity	−0.00237	0.000	−12.580	0.000
	*n*	−0.00023	0.000	−43.530	0.000
NR-OR	Intercept	0.21100	0.018	12.035	0.001
	Non-sparsity	−0.00125	0.000	−7.235	0.000
	*n*	−0.00019	0.000	−38.923	0.000
Ridge	Intercept	0.21950	0.003	63.860	0.000
	Non-sparsity	−0.00744	0.000	−24.470	0.000
	*n*	−0.00015	0.000	−18.460	0.000

### Simulation study based on real data

**Figure 5:**
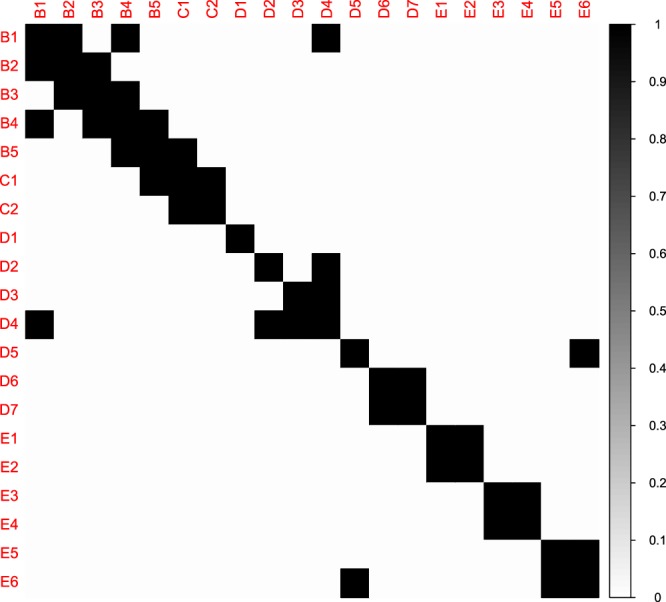
Heatmap of the true network based on the data on 20 PTSD symptoms. White represents partial correlations equal to zero, and black represents partial correlations different from zero.

In this section we simulate data based on the sparse GGM results obtained by Armour, *et al*.^3^ for 20 Post-Traumatic Stress Disorder (PTSD) symptoms of 221 U.S. military veterans. The 20 PTSD symptoms are assumed to form four symptoms clusters: intrusions (B1-B5), avoidance (C1-C2), negative alterations in cognitions and mood (D1-D7), and alterations in arousal and reactivity (E1-E6). Armour, *et al*.^3^ applied the Glasso-EBIC approach and used bootstrapping techniques to estimate the parameter accuracy and stability of the partial correlation matrix Γ^26^. The associated network, shown in Fig. 5, reveals strong positive within-cluster connections between nightmares (B2) and flashbacks (B3), blame of self or others (D3) and negative trauma related emotions (D4), detachment (D6) and restricted affect (D7), and hypervigilance (E3) and exaggerated startle response (E4). On top of that, they also find many moderately positive connections within the symptom clusters: for instance, intrusive thoughts (B1) and nightmares (B2), avoidance thoughts (C1) and avoidance remainders (C2), irritability/anger (E1) and self-destructive behaviour (E2), but also between symptom clusters, for instance between loss of interest (D5) and difficulty in concentrating (E5).

To compare the performance before and after using PCS, we drew *n* observations from a 20-variate Gaussian distribution with mean zero and partial correlation matrix Γ. We used two sample sizes *n* = {100, 500} and replicated the simulation 100 times.

**Figure 6:**
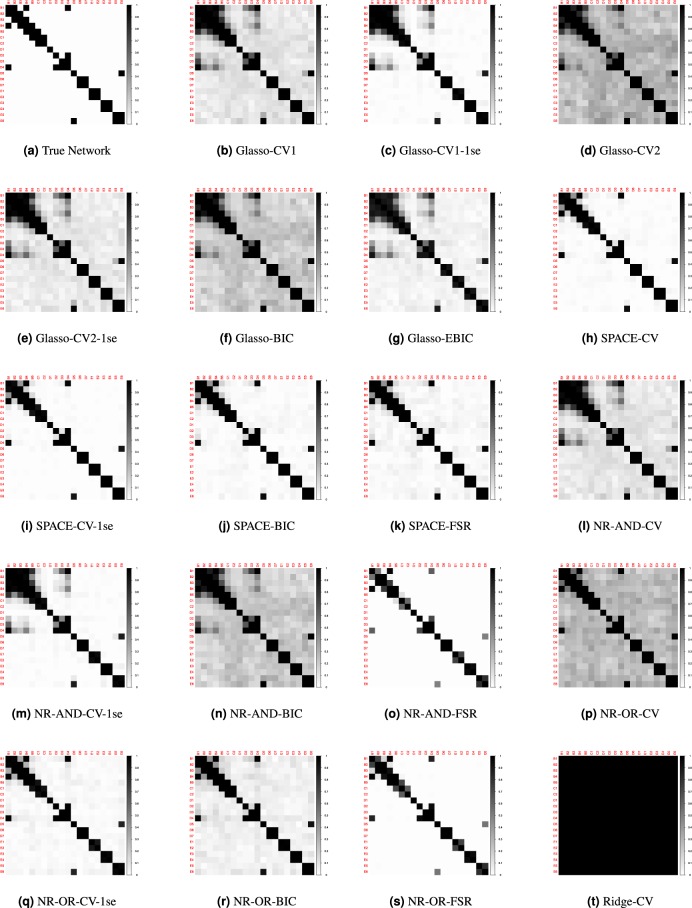
Heatmaps of the frequency with which the edges for the PTSD data based simulations (*n* = 100) are set to zero by the different methods before applying PCS. White indicates that an edge was excluded from the network in all replications, whereas black reflects that the edge was always retained in the network.

**Figure 7:**
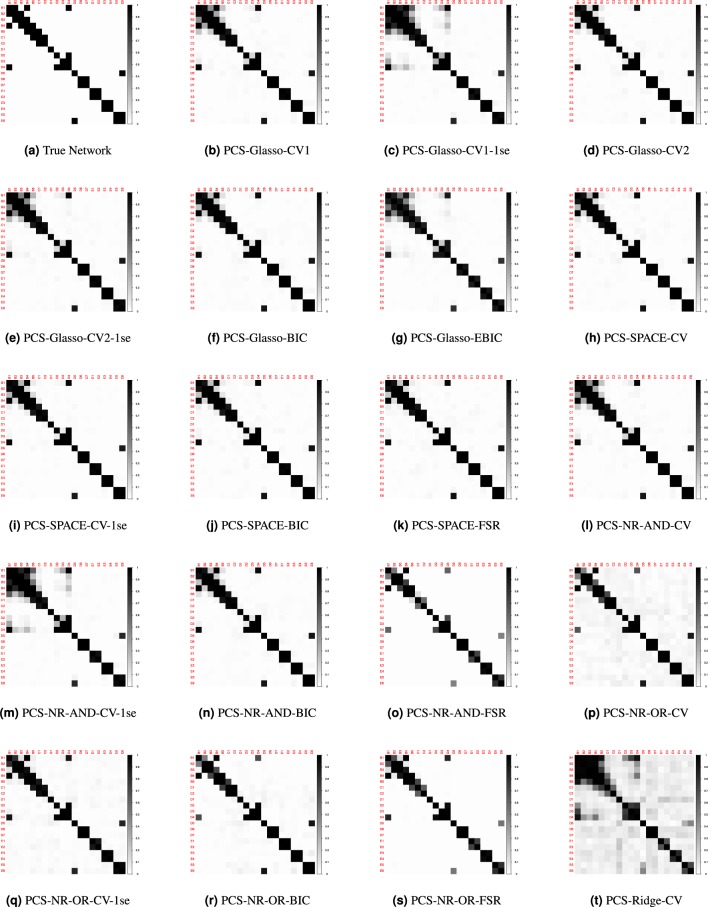
Heatmaps of the frequency with which the edges for the PTSD data based simulations (*n* = 100) are set to zero by the different methods after applying PCS. White indicates that an edge was excluded from the network in all replications, whereas black reflects that the edge was always retained in the network.

Table 8 shows the average TPR and FPR scores and Figs. 6 and 7 present heatmaps of the frequency with which the entries of the partial correlation matrix are detected as non-zero. We observe that Partial Correlation Screening (PCS) significantly outperforms Glasso, NR and SPACE. When *n* = 100, PCS-SPACE-BIC has the best performance in terms of the false positive rate, which is in line with the simulation results on synthetic data. For *n* = 500, all the estimation procedures using PCS show an average TPR higher than 0.999 and an average FPR below 0.020 (see Table 8).

**Table 8:** Average true positive rate (TPR) and false positive rate (FPR) over 100 simulations based on the PTSD data.

	*n* = 100				*n* = 500			
	TPR		FPR		TPR		FPR	
	no-PCS	PCS	no-PCS	PCS	no-PCS	PCS	no-PCS	PCS
Glasso-CV	0.999	0.974	0.145	0.013	1.000	1.000	0.070	0.007
Glasso-CV-1se	0.979	0.968	0.052	0.031	1.000	1.000	0.055	0.011
Glasso-CV2	1.000	0.968	0.295	0.007	1.000	1.000	0.274	0.002
Glasso-CV2-1se	0.999	0.972	0.125	0.015	1.000	1.000	0.104	0.008
Glasso-BIC	1.000	0.973	0.229	0.009	1.000	1.000	0.240	0.004
Glasso-EBIC	0.908	0.889	0.088	0.017	1.000	1.000	0.141	0.006
SPACE-CV	0.983	0.983	0.015	0.014	1.000	1.000	0.013	0.009
SPACE-CV-1se	0.978	0.978	0.010	0.009	1.000	1.000	0.009	0.007
SPACE-BIC	0.989	0.988	0.022	0.020	1.000	1.000	0.015	0.010
SPACE-FSR	0.995	0.993	0.032	0.029	1.000	1.000	0.026	0.018
NR-AND-CV	0.995	0.908	0.092	0.017	1.000	1.000	0.077	0.002
NR-AND-CV-1se	0.887	0.879	0.005	0.003	1.000	1.000	0.001	0.001
NR-AND-BIC	0.976	0.931	0.017	0.006	1.000	1.000	0.009	0.001
NR-AND-FSR	0.754	0.751	0.000	0.000	0.999	0.999	0.001	0.000
NR-OR-CV	0.999	0.883	0.270	0.024	1.000	1.000	0.254	0.002
NR-OR-CV-1se	0.965	0.943	0.025	0.011	1.000	1.000	0.007	0.001
NR-OR-BIC	0.991	0.897	0.063	0.007	1.000	0.999	0.038	0.001
NR-OR-FSR	0.855	0.850	0.002	0.002	1.000	1.000	0.002	0.001
Ridge-CV	1.000	0.845	1.000	0.116	1.000	1.000	1.000	0.005

Note: Standard errors for TPR range from 0.000 to 0.029 and standard errors for FPR range from 0.000 to 0.028.

### Breast cancer data

GGMs have been widely applied to analyze gene expression data, since many authors hypothesize that the complex interactions between genes take the form of sparse pathways or networks^27–29^. More specifically, given mRNA levels of different patients, researchers have studied the conditional dependencies of genes for a variety of diseases^1^.

We estimate a sparse partial correlation network for gene expression data from a breast cancer study by West *et al*.^30^. The dataset contains 7, 129 genes sampled from 49 breast tumor tissues samples: 25 samples from patients diagnosed as estrogen receptor positive and 24 samples from patients diagnosed as estrogen receptor negative. In line with Sheridan *et al*.^31^, we focus on a subset of *p* = 150 genes related to the estrogen receptor gene ERS1. This gene acts as an estrogen-activated transcription factor and has a key role in the proliferation of cancerous cells^32^.

Table 9 shows how many edges are obtained with the Glasso, NR, SPACE and Ridge techniques under consideration and how much these numbers of edges decrease by applying PCS. It can be concluded that the sparsity level varies considerably depending on the approach used. We observe that when the procedures yield dense networks (i.e. Ridge-CV, Glasso-CV1, Glasso-CV1-1se, Glasso-CV2, Glasso-CV2-1se and NR-OR-CV), applying PCS produces a larger reduction in the number of edges.

**Table 9:** Estimated number of edges of the gene regulatory network for the breast cancer data, the symptom network of patients with a diagnosis within the nonaffective psychotic spectrum using the BPRS scale and the symptom network of patients with PTSD.

	Breast Cancer		BPRS		PTSD	
	no-PCS	PCS	no-PCS	PCS	no-PCS	PCS
Glasso-CV	2,318	461	172	37	126	67
Glasso-CV-1se	1,799	544	116	77	100	91
Glasso-CV2	1,799	544	124	78	109	68
Glasso-CV2-1se	1,712	640	94	84	95	56
Glasso-BIC	0	0	132	81	114	67
Glasso-EBIC	0	0	0	0	104	100
SPACE-CV	577	576	82	82	70	66
SPACE-CV-1se	433	431	62	62	57	57
SPACE-BIC	437	437	72	72	57	57
SPACE-FSR	562	561	74	74	69	61
NR-AND-CV	608	608	85	85	80	75
NR-AND-CV-1se	287	287	29	29	45	44
NR-AND-BIC	417	417	69	69	58	56
NR-AND-FSR	38	38	26	25	28	28
NR-OR-CV	1,435	820	122	122	113	88
NR-OR-CV-1se	644	644	50	46	56	54
NR-OR-BIC	943	572	87	87	77	77
NR-OR-FSR	105	102	41	39	44	43
Ridge-CV	11,175	631	276	115	190	109

Given that the results vary considerably across the methods, the next question is how we should deal with this uncertainty when interpreting the networks. We opt to combine the results of the different estimation methods^33,34^, by computing a network that includes all edges that occur in at least two of the nineteen obtained PCS networks. Note that if we apply this combination approach to the estimated PCS networks for the toy example (see Fig. 3), we would recover the true network.

**Figure 8:**
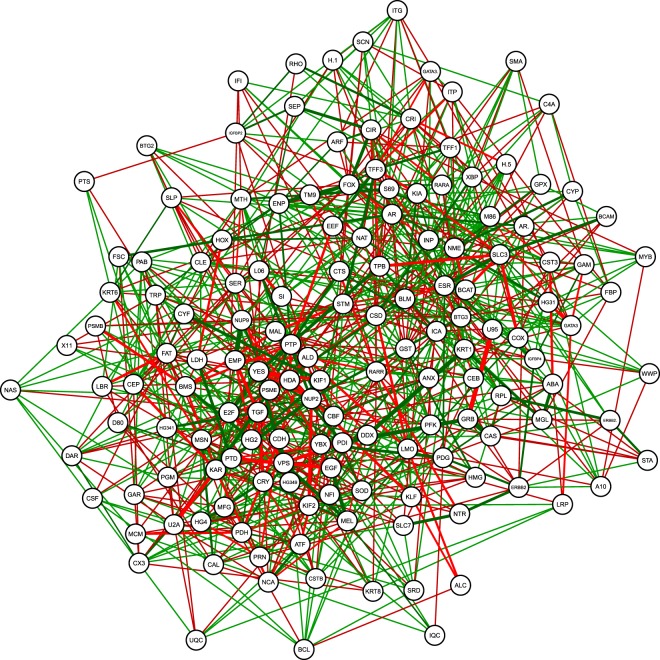
Estimated gene regulatory network for the breast cancer data.

**Figure 9:**
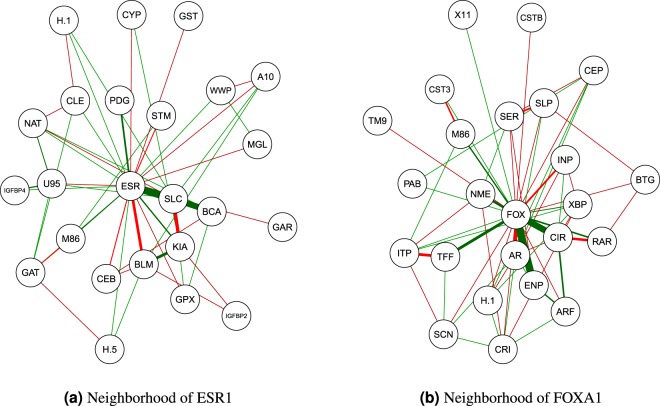
Estimated sub-network of genes in the neighborhood of ESR1 and FOXA1 for the breast cancer data.

Figure 8 shows the resulting combined network for the breast cancer data. Figure 9 focuses on the sub-network of the genes that are related with the estrogen receptor gene ERS1 (Panel a) and the gene FOXA1 (Panel b). We can identify some important regularity interactions in the estimated GGM. As a first example, the ESR1 (ESR) gene is partially correlated with SLC39A6 (SLC). This gene functions as a zinc transporter and has been shown to be highly expressed in ESR1-positive tumours and is highly significantly associated with the spread of breast cancer to the lymph nodes^32^. As a second example, we can inspect the genes that belong to the neighborhood of FOXA1 (FOX). FOXA1 has been found to be predominantly expressed in luminal type A carcinomas^35^ and may prevent metastatic progression of this type of breast cancer^36^. We observe an edge between FOXA1 (FOX) and AR (AR) (androgen receptor), which is in line with findings that indicate that AR regulates estrogen receptor expression^37^.

### Psychopathological symptoms data

For a long time, modeling approaches to psychopathological data started from the assumption that psychopathological symptoms reflect an underlying mental disorder and thus are caused by this disorder^38^. This assumption has recently been challenged and an alternative hypothesis has been put forward stating that symptoms are causally active components of a mental disorder^39,40^. Within this framework, network analysis is then used to study the conditional dependencies between a set of symptoms^41,42^.

We studied the conditional dependencies of a set of 24 psychopathological symptoms in a sample of 184 patients (189 before patients with missing data were discarded) within the nonaffective psychotic spectrum, that participated in the second wave of the multicenter Genetic Risk and Outcome of Psychosis (GROUP) cohort study^43^. The symptoms are measured using the Brief Psychiatric Rating Scale (BPRS)^44^, which captures the following symptoms: Somatic Concern (SmC), Anxiety (Anx), Depression (Dpr), Guilt (Glt), Hostility (Hst), Suspiciousness (Ssp), Unusual Thought (UnT), Grandiosity (Grn), Hallucinations (Hll), Disorientation (Dsr), Conceptual Disorganization (CnD), Excitement (Exc), Elevated mood (ElM), Tension (Tns), Mannerisms (Mnn), Uncooperativeness (Unc), Motor Retardation (MtR), Suicidality (Scd), Self Neglect (SlN), Bizarre Behaviour (BzB), Motor Hyperactivity (MtH), Distractibility (Dst), Emotional Withdrawal (EmW) and Blunted Affect (BlA). Each symptom is rated on a 7-point Likert scale. Because the data is measured on a Likert scale rather than on a continuous one, we apply the nonparanormal transformation proposed by Liu *et al*.^45^ that uses the Gaussian copula to transform the data into normal scores.

Table 9 shows the number of edges that result from applying the different methods under consideration. We observe that Ridge-CV, Glasso-CV1, NR-OR-CV and Glasso-BIC estimate the most dense networks and that applying PCS drastically reduces the amount of edges when the original network was not so sparse.

**Figure 10:**
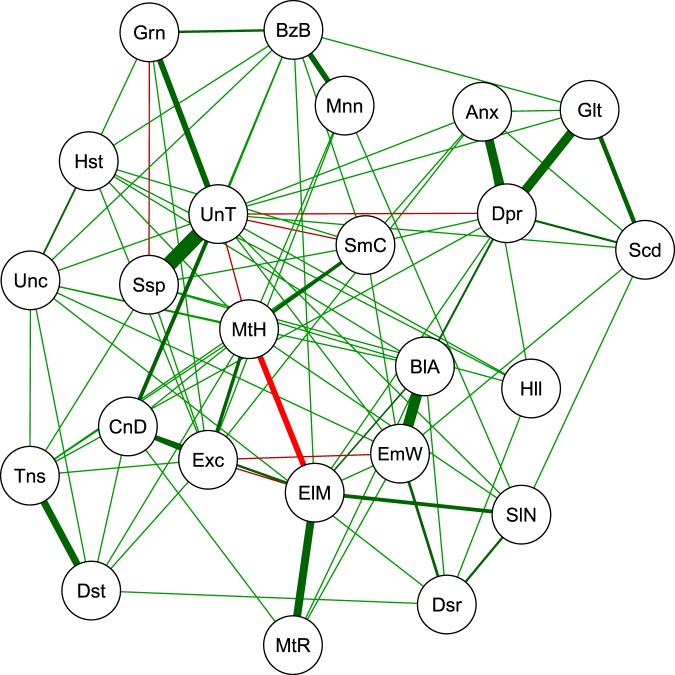
Estimated symptoms network of patients with a diagnosis within the nonaffective psychotic spectrum using the BPRS data.

Figure 10 shows the network computed by combining the different PCS networks and discarding edges all that occur only once. Cognitive models that study psychosis have postulated that some of the most prominent symptoms are delusional beliefs (grandiosity, suspiciousness, unusual thoughts)^46^. We indeed observe that there is strong positive relation between Unusual Thoughts (UnT) and Suspiciousness (Ssp) and between Emotional Withdrawal (EmW) and Blunted Affect (BlA). Also, there is a strong positive relation between Unusual Thoughts (UnT) and Grandiosity (Grn), Motor Retardation (MtR) and Elevated mood (ElM), Anxiety (Anx) and Depression (Dpr), Depression (Dpr) and Guilt (Glt), and Tension (Tns) and Distractibility (Dst).

### Post-traumatic stress disorder symptoms data

**Figure 11:**
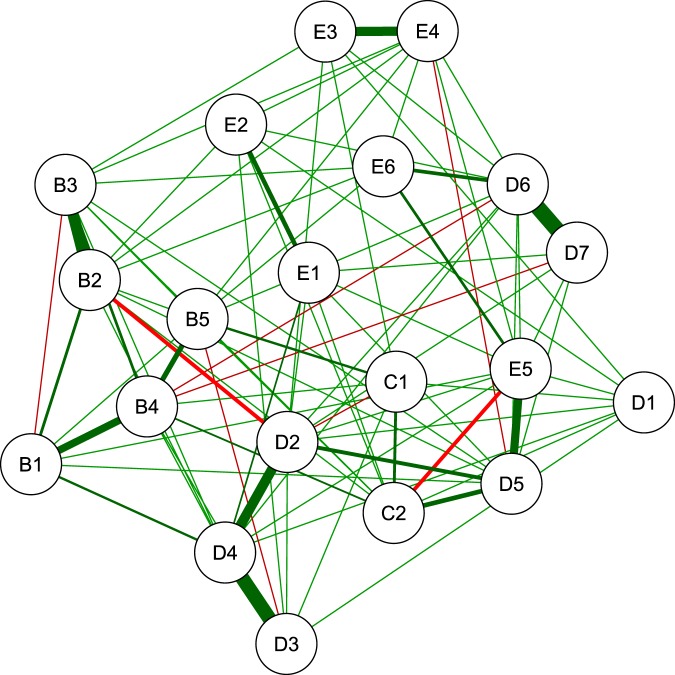
Estimated symptoms network for the PTSD data.

Finally, we return to the PTSD data that we studied in Subsection: Simulation Study Based on Real Data. Table 9 shows the number of edges for each of the procedures. We observe a similar pattern as in the previous applications. Figure 11 displays the network that results from applying our combination approach to the PCS networks. This combined network recovers the conditional dependencies that Armour *et al*.^3^ found to be strongly positive: nightmares (B2) and flashbacks (B3), blame of self or others (D3) and negative trauma related emotions (D4), detachment (D6) and restricted affect (D7), and hypervigilance (E3) and exaggerated startle response (E4).

## Discussion

In this article, we have demonstrated through an extensive simulation study that the most popular procedures to estimate partial correlation networks, Glasso, SPACE, NR and Ridge, often do not yield the true underlying network, no matter which procedure is applied to select the regularization parameter. Results are heavily influenced by sample size and the number of variables (i.e., the lower the sample size and the higher the number of variables, the worse), with high-dimensional problems being especially difficult. We also note that the Glasso results heavily depend on which approach is used to tune the regularization parameter. Specifically, we found that in the high-dimensional setting, using the BIC or EBIC yields many false negatives and thus an overly sparse network.

Given that the state-of-the-art methods frequently cannot satisfactorily recover the true set of edges, we have presented a novel approach that allows to better control the false positive rate. This procedure boils down to performing an additional second step, after applying one or more state-of-the-art methods of choice. In this second step, we discard the partial correlation coefficients in the estimated network that are smaller in absolute value than a given threshold, which is obtained through cross-validation. Our novel procedure clearly improved the performance of the estimation methods and tuning approaches considered, especially in the settings where the state-of-the-art methods yielded bad results. Whereas PCS-SPACE-BIC seems to be the best choice for small sample size, which method is applied in the first step hardly matters when sample size increases.

We also applied all approaches to three real data sets. The results again show that our PCS approaches yield more sparse networks than the state-of-the art methods. To deal with the multitude of obtained networks, we proposed to compute a network that combines the different PCS estimates, but discard the edges that occurred in only one network. Although results seemed interpretable, future research should investigate further how to efficiently combine the different estimators or how to optimally select among the nineteen obtained networks.

In this paper we used standard simulation settings from the literature to demonstrate the problematic behaviour of existing approaches. It is important to mention that except for Glasso, none of the state-of-the-art procedures studied in this paper estimates a covariance matrix that is positive definite. Also, it is not guaranteed that this property still holds after applying the PCS to Glasso. In future research, it would be useful to investigate the behavior of the different approaches under more difficult settings as well as the theoretical properties of the PCS. This would lead to several possible extensions of our method. One extension targets data in which the assumption of multivariate normality is violated. Here, our approach can be easily extended to make use of techniques to estimate semiparametric undirected graphs^45,47,48^. We also note that in some applications, such as in psychology data or in the high dimensional setting, some variables might be highly linearly correlated. In this setting, the assumption regarding the regularity of the covariance matrix might not hold. A possible solution is to first cluster the strongly correlated variables and then take this cluster structure into account when estimating the GGM using the PCS approach^49,50^.

Finally, it is important to note that imposing sparsity might be too stringent in some applications. For instance, in some cases researchers are also interested in detecting partial correlations that are very close to zero. Moreover, it can also happen that the true network is not so sparse to begin with. In such cases, using approaches based on ${\ell }_{1}$ regularization may affect the validity of the results^51^. Therefore, we believe that future research should also focus on exploring how the methods proposed in this paper behave when the true underlying network is less sparse or includes some very weak edges.

## Methods

### Partial correlation estimation procedures

In this subsection we present the technical details of the state-of-the-arts methods to estimate sparse partial correlation networks and the associated tuning methods for the regularization parameter.

#### The graphical lasso

Yuan and Lin^25^ and Rothman *et al*.^24^ proposed a penalized maximum likelihood approach to estimate the inverse of the covariance matrix Σ, denoted by Ω = [*ω*_*ij*_]. If *S* denotes the sample covariance matrix, the problem is to minimize the following penalized log-likelihood function:

5 $$\hat{{\boldsymbol{\Omega }}}({\lambda }_{1})=\mathop{{\rm{a}}{\rm{r}}{\rm{g}}{\rm{m}}{\rm{a}}{\rm{x}}}\limits_{{\boldsymbol{\Omega }}\succ 0}\{{\rm{t}}{\rm{r}}({\bf{S}}{\boldsymbol{\Omega }})-\,\log \,det({\boldsymbol{\Omega }})+{\lambda }_{1}\sum _{i\ne j}\,|{\omega }_{ij}|\},$$

where tr(⋅) denotes the trace of a matrix and *λ*_1_ > 0 controls the size of the penalty. The penalty term is a proxy of the number of zeros in the precision matrix. The smaller the value of *λ*_1_, the more non-zero elements the model includes. Friedman, Hastie and Tibshirani^13^ proposed an efficient algorithm to implement this method, which is called the Graphical lasso (Glasso). Afterwards, the partial correlation matrix can be computed using the known relation between the entries of the inverse of the covariance matrix and the partial correlation coefficients (see Lemma 1 in Peng *et al*.^12^).

For the different applications we select the regularization parameter as follows. We generate a grid of 100 equidistant possible values for *λ*_1_ ranging from 0.001 to |max(**S**)| when *p* < 100. When *p* ≥ 100 the sequence limits are (0.05,|max(**S**)|). We propose six approaches to select the optimal value from this grid. The first one is to implement *K*-fold cross-validation using the log-likelihood as performance measure (see Section 4.2 in Huang *et al*.^52^ and Section 2.3 in Price *et al*.^53^). We denote this procedure Glasso-CV1. We split the sample in *K* subsets. Using all but the *k*-th subset, we estimate the precision matrix using Glasso and denote this matrix $\hat{\Omega }$, for different values of *λ*_1_. On the basis of the discarded *k*-th subset we estimate the sample covariance matrix, **S**^*k*^. Next, for each value of *λ*_1_ we compute the following loss function:

6 $${\rm{C}}{\rm{V}}1({\lambda }_{1})=\mathop{\sum }\limits_{k=1}^{K}\,\{{\rm{t}}{\rm{r}}({{\bf{S}}}^{k}\hat{{\boldsymbol{\Omega }}}({\lambda }_{1}))-\,\log \,det(\hat{{\boldsymbol{\Omega }}}({\lambda }_{1}))\}.$$

We plot CV1(*λ*_1_) versus *λ*_1_ and we select the tuning parameter that minimizes the loss function CV1(*λ*_1_).

The second approach uses the one-standard-error-rule^20^. We denote this procedure Glasso-CV1-1se. Using the loss function in Eq. (6), we first compute the standard deviation of CV1_1_(*λ*_1_), …, CV1_*K*_(*λ*_1_):

7 $${\rm{sd}}({\lambda }_{1})={\rm{sd}}({\rm{CV}}{1}_{1}({\lambda }_{1}),\ldots ,{\rm{CV}}{1}_{K}({\lambda }_{1})).$$

Next, we compute the standard error of CV1(*λ*_1_):

8 $${\rm{se}}({\lambda }_{1})={\rm{sd}}({\lambda }_{1})/\sqrt{K}.$$

Finally, given the tuning weight that minimizes the cross-validation error in Eq. (6), denoted by ${\hat{\lambda }}_{1}$, we choose the tuning weight that verifies the following rule:

9 $${\rm{CV}}1({\lambda }_{1})\le {\rm{CV}}1({\hat{\lambda }}_{1})+{\rm{se}}({\hat{\lambda }}_{1})$$

The third approach implements *K*-fold cross-validation using the prediction errors of each node as performance measure. We denote this procedure Glasso-CV2. We split the sample in *K* subsets. Using all but the *k*-th subset, we estimate the precision matrix using Glasso and denote this matrix $\hat{\Omega }$, for different values of *λ*_1_. Next, for each value of *λ*_1_ we compute the following loss function:

10 $${\rm{CV}}2({\lambda }_{1})=\mathop{\sum }\limits_{k=1}^{K}\,\mathop{\sum }\limits_{i=1}^{p}\,{\left\Vert {X}_{i}^{k}-\sum _{j\ne i}\left(-\frac{{\hat{\omega }}_{ij}}{{\hat{\omega }}_{ii}}\right){X}_{j}^{k}\right \Vert}^{2}.$$

We plot CV2(*λ*_1_) versus *λ*_1_ and we select the tuning parameter that minimizes the loss function CV2(*λ*_1_).

The fourth procedure selects the tuning weight by applying the one-standard-error-rule on the cross-validation procedure CV2. We denote this procedure Glasso-CV2-1se.

The fifth and sixth procedures to select the optimal regularization parameter from the 100 considered *λ*_1_ values are based on the Bayesian Information Criterion (BIC) or the Extended Bayesian Information Criterion (EBIC). We refer to these procedures as Glasso-BIC and Glasso-EBIC, respectively. We select the value of *λ*_1_ that minimizes the following loss function:

11 $${\rm{EBIC}}({\lambda }_{1})=-\,2 {\mathcal L} (\hat{{\boldsymbol{\Omega }}}({\lambda }_{1}))+\kappa \,\log (n)+4\kappa \gamma \,\log (p)$$

where ${\mathcal L} (\cdot )$ is the value of the log-likelihood function that corresponds to the estimated matrix $\hat{\Omega }$, *κ* is the number of edges in the estimated network and *γ* ∈ [0, 1] is a parameter that controls the penalization of the network. If *γ* = 0, the Eq. (11) corresponds to the classical BIC. Positive values of *γ* lead to stronger penalization. To compute EBIC, we follow the recommendation of Chen and Chen^54^ and Foygel and Drton^21^ and set *γ* to 0.5^55,56^.

#### Nodewise regression

Meinshausen and Bühlmann^11^ proposed to estimate the set of network edges by performing *p* separate lasso regressions:

12 $${\hat{{\boldsymbol{\beta }}}}_{i}({\lambda }_{2})=\mathop{{\rm{a}}{\rm{r}}{\rm{g}}{\rm{m}}{\rm{i}}{\rm{n}}}\limits_{{\beta }_{ij}}\left\{\frac{1}{2}{\left\Vert {X}_{i}-\sum _{j\ne i}{\beta }_{ij}{X}_{j}\right\Vert }^{2}+{\lambda }_{2}\sum _{j\ne i}\,|{\beta }_{ij}|\right\},$$

where ${\hat{\beta }}_{i}$ is a vector that contains the *p* − 1 estimated regression weights of node *i* and *λ*_2_ > 0 is the regularization parameter that controls the number of non-zero elements in the neighborhood of node *i*. The set of edges can be computed with the AND-rule:

estimate an edge between nodes *i* and *j* ⇔ ${\hat{\beta }}_{ij}$ ≠ 0 and ${\hat{\beta }}_{ji}$ ≠ 0

yielding the NR-AND procedure.

Alternatively, we can use the NR-OR method and compute the edge set with the OR-rule:

estimate an edge between nodes *i* and *j* ⇔ ${\hat{\beta }}_{ij}$ ≠ 0 or ${\hat{\beta }}_{ji}$ ≠ 0.

Next, the partial correlation matrix can be computed using the relation between the prediction errors of the best linear predictor of each node and the partial correlation coefficients (see Lemma 1 in Peng *et al*.^12^).

To select the tuning parameter *λ*_2_ for each regression separately we generate a grid of 100 possible values using the sequence generated with the function glmnet of the R package glmnet^57^. We consider four different tuning procedures. First, we can perform *K*-fold cross-validation. Discarding the *k*-th subset we estimate the vector of regression weights ${\hat{\beta }}_{i}$ using a lasso regression. We select the value of *λ*_2_ that minimizes the following loss function:

13 $${\rm{CV}}({\lambda }_{2})=\mathop{\sum }\limits_{k=1}^{K}\,{\left\Vert {X}_{i}^{k}-\sum _{j\ne i}{\hat{\beta }}_{ij}{X}_{j}^{k}\right\Vert }^{2},$$

where *X*_*i*_^*k*^ are the observations in the discarded subset *k*.

The second approach adapts this cross-validation approach by using the one-standard-error-rule. We denote this procedure NR-CV-1se.

The third procedure to select the regularization parameter, NR-BIC, involves computing the Bayesian Information Criterion (BIC) for different values of *λ*_2_. For each node, we select the value of *λ*_2_ that minimizes the following loss function:

14 $${{\rm{BIC}}}_{i}({\lambda }_{2})=n{\rm{RSS}}({\hat{{\boldsymbol{\beta }}}}_{i})+{\kappa }_{i}\,\log (n)$$

where RSS(⋅) is the value of the residual sum of squares for the *i*-th regression and *κ*_*i*_ is the number of elements in the estimated neighborhood of node *i*.

The fourth procedure is NR-FSR and uses a Finite Sample Result. Meinshausen and Bühlmann^11^ show that under certain assumptions regarding the sparsity and regularity conditions of the covariance matrix and the regression weights, the neighborhood of a node *i* will contain at most *α* ∈ (0, 1) false positive edges if the ${\ell }_{1}$ penalty parameter is set as: ${\lambda }_{2}(\alpha )=\frac{2}{\sqrt{n}}{\Phi }^{-1}(1-\frac{\alpha }{2{p}^{2}})$, where Φ^−1^ is the inverse of the c.d.f. of *N*(0, 1). We set the bound to the proportion of the false positive edges to *α* = 0.05.

#### Joint sparse linear regression

Peng *et al*.^12^ proposed to estimate the partial correlation matrix by minimizing the following joint sparse regression (SPACE):

15 $$\hat{\Gamma }({\lambda }_{3})=\mathop{{\rm{a}}{\rm{r}}{\rm{g}}{\rm{m}}{\rm{i}}{\rm{n}}}\limits_{{\rho }_{ij},{\omega }_{ii}}\left \{\frac{1}{2}\left(\mathop{\sum }\limits_{i=1}^{p}\,{\left\Vert {X}_{i}-\sum _{j\ne i}{\rho }_{ij| V{\rm{\setminus }}\{i,j\}}\sqrt{\frac{{\omega }_{jj}}{{\omega }_{ii}}}{X}_{j}\right\Vert }^{2}\right)+{\lambda }_{3}\sum _{1\le i < j\le p}\,|{\rho }_{ij}|\right\},$$

where *ω*_*ii*_ is the residual variance of the optimal prediction of *X*_*i*_ given all remaining variables, which is equivalent to the the i-th diagonal element of the matrix Ω and *λ*_3_ > 0 is the regularization parameter that controls the number of non-zero elements in the partial correlation matrix Γ.

Given a grid of 100 equidistant values for *λ*_3_ ranging from $\sqrt{n}{\Phi }^{-1}(1-\frac{0.9}{2{p}^{2}})$ to $\sqrt{n}{\Phi }^{-1}(1-\frac{1e-4}{2{p}^{2}})$, there are four different procedures to calibrate the tuning parameter *λ*_3_. We first propose to perform K-fold cross-validation, yielding SPACE-CV. We first split the sample into *K* subsets and select the parameter value that minimizes the following loss function:

16 $${\rm{C}}{\rm{V}}({\lambda }_{3})=\mathop{\sum }\limits_{k=1}^{K}\,\mathop{\sum }\limits_{i=1}^{p}\,{\left\Vert {X}_{i}^{k}-\sum _{j\ne i}{\hat{\rho }}_{ij| V{\rm{\setminus }}\{i,j\}}\sqrt{\frac{{\hat{\omega }}_{jj}}{{\hat{\omega }}_{ii}}}{X}_{j}^{k}\right\Vert }^{2}.$$

The second procedure again adapts this cross-validation approach by using the one-standard-error-rule. We denote this procedure SPACE-CV-1se.

The third procedure to select the regularization parameter involves computing the Bayesian Information Criterion (BIC) for the 100 values of *λ*_3_. First, we compute for each node the residual sum of squares:

$${{\rm{R}}{\rm{S}}{\rm{S}}}_{i}({\hat{\rho }}_{ij| V{\rm{\setminus }}\{i,j\}},{\hat{\omega }}_{ii})={\left\Vert {X}_{i}-\sum _{j\ne i}{\hat{\rho }}_{ij| V{\rm{\setminus }}\{i,j\}}\sqrt{\frac{{\hat{\omega }}_{jj}}{{\hat{\omega }}_{ii}}}{X}_{j}\right\Vert }^{2},$$

Next, we select the value of *λ*_3_ by minimizing:

17 $${\rm{B}}{\rm{I}}{\rm{C}}({\lambda }_{3})=\mathop{\sum }\limits_{i=1}^{p}\,\left(n{{\rm{R}}{\rm{S}}{\rm{S}}}_{i}({\hat{\rho }}_{ij| V{\rm{\setminus }}\{i,j\}},{\hat{\omega }}_{ii})+{\kappa }_{i}\,\log (n)\right)$$

where *κ*_*i*_ is the number of elements in the estimated neighborhood of node *i*.

The fourth procedure SPACE-FSR is based on the Finite Sample Result by Peng *et al*.^12^. These authors show that under certain assumptions regarding the sparsity and regularity conditions of the covariance matrix and the regression weights, the neighborhood of a node *i* will contain at most *α* ∈ (0, 1) false positive edges if the penalty parameter is set as: ${\lambda }_{3}(\alpha )=\sqrt{n}{\Phi }^{-1}(1-\frac{\alpha }{2{p}^{2}})$, where Φ^−1^ is the inverse of the c.d.f. of *N*(0, 1). We again set the bound to the proportion of the false positive edges to *α* = 0.05.

#### Partial correlation estimation using ridge regression

Ha and Sun^19^ proposed to estimate a penalized partial correlations matrix using a ridge penalty. We apply a simpler version of their method by performing *p* separate ridge regressions:

18 $${\hat{{\boldsymbol{\delta }}}}_{i}({\lambda }_{4})=\mathop{{\rm{a}}{\rm{r}}{\rm{g}}{\rm{m}}{\rm{i}}{\rm{n}}}\limits_{{\delta }_{ij}}\left\{\frac{1}{2}{\left\Vert {X}_{i}-\sum _{j\ne i}{\delta }_{ij}{X}_{j}\right\Vert }^{2}+{\lambda }_{4}\sum _{j\ne i}\,{\delta }_{ij}^{2}\right\},$$

where ${\hat{\delta }}_{i}$ is a vector that contains the *p* − 1 estimated regression weights for node *i* and *λ*_4_ > 0 is the regularization parameter that controls the amount of shrinkage of the regression weights toward zero in the neighborhood of node *i*. The part*i*al correlation matrix is computed using the relation between the prediction errors of the best linear predictor of each node and the partial correlation coefficients (see Lemma 1 in Peng *et al*.^12^).

To select the tuning parameter *λ*_4_ for each regression separately we generate a grid of 100 possible values using the sequence generated with the function glmnet of the R package glmnet^57^. We select the regularization parameter by performing *K*-fold cross-validation. Discarding the *k*-th subset we estimate the vector of regression weights ${\hat{\delta }}_{i}$ using ridge regression. We select the value of *λ*_4_ that minimizes the following loss function:

19 $${\rm{CV}}({\lambda }_{4})=\mathop{\sum }\limits_{k=1}^{K}\,{\left\Vert {X}_{i}^{k}-\sum _{j\ne i}{\hat{\delta }}_{ij}{X}_{j}^{k}\right\Vert }^{2},$$

where *X*_*i*_^*k*^ are the observations in the discarded subset *k*. We denote this procedure Ridge-CV.

### Partial correlation screening procedure

In this subsection we present the technical details of the Partial Correlation Screening (PCS) algorithm. The procedure estimates the set of edges in two steps. In the first step, we determine a sparse partial correlation network, denoted by $\hat{\Gamma }=[{\hat{\rho }}_{ij|V\backslash \{i,j\}}]$, using one of the methods that we discussed in the previous subsection.

In the second step of the algorithm, we detect unimportant pairs of variables by thresholding the partial correlations estimated in the first step. For *i* ∈ *V* and a threshold parameter *τ* ∈ (0, 1), we estimate the neighborhood of node *i* as follows

20 $${\hat{\mathscr A}}_{i,\tau }=\{j\in V\backslash \{i\}:|{\hat{\rho }}_{ij|V\backslash \{i,j\}}| > \tau \}.$$

The algorithm outputs the estimated set of edges for a given threshold *τ*:

21 $${\hat{E}}_{\tau }=\{(i,j)\in V:|{\hat{\rho }}_{ij|V\backslash \{i,j\}}| > \tau \}.$$

Finally, the prediction error of the regression of each node *i* conditioned on the variables that belong to the estimated neighborhood set ${\hat{\mathscr A}}_{i,\tau }$ is given by

$${\hat{\varepsilon }}_{i,\tau }={X}_{i}-\sum _{j\in {\hat{\mathscr A}}_{i,\tau }}\,{\hat{\theta }}_{ij,\tau }{X}_{j},$$

where ${\hat{\theta }}_{i,\tau }$ is the vector of estimated regression coefficients of node *i* ∈ *V* given the variables in the estimated neighborhood set ${\hat{\mathscr A}}_{i,\tau }$.

#### Choice of the tuning parameter

To select the threshold parameter *τ*, we perform *K*-fold cross validation. We generate a sequence of 100 equidistant values for the threshold *τ* ranging from 0.0001 to 1. The procedure to select the threshold uses a double-loop. First, for each of the estimation procedures proposed in the previous subsection, we select the regularization parameter *λ*. Second, we split the sample in *K* subsets. Using all but the *k*-the subset, we estimate a sparse partial correlation network using the selected regularization parameter *λ*. Next, for each value of *τ* in the grid, we estimate the neighborhood of each node (see Eq. (20)) and the regression weights vector ${\hat{\theta }}_{i,\tau }$. For each value of *τ* we compute the following loss function:

22 $$CV(\tau )=\mathop{\sum }\limits_{k=1}^{K}\,\mathop{\sum }\limits_{i=1}^{p}\,{\left\Vert {X}_{i}^{k}-\sum _{j\in {\hat{\mathscr A}}_{i,\tau }}{\hat{\theta }}_{ij,\tau }{X}_{j}^{k}\right\Vert }^{2}.$$

We plot *CV*(*τ*) versus *τ* and we select the threshold parameter that minimizes the loss function *CV*(*τ*).

## Supplementary information

Covariance matrix for Psychopathological Symptoms Data

R Code Simulation Synthetic Data
