# Supplementary material for: A Partial Correlation Screening Approach for Controlling the False Positive Rate in Sparse Gaussian Graphical Models
Source: Sci Rep. 2019 Nov 28;9:17759. doi: 10.1038/s41598-019-53795-x (PMC6882820; doi:10.1038/s41598-019-53795-x)
Supplement: Supplementary file 2 — R Code Simulation Synthetic Data [file 41598_2019_53795_MOESM2_ESM.pdf]

```
############################################################
############################################################
#####         R Code by Ginette Lafit        ###############
#####         ginette.lafit@kuleuven.be      ###############
############################################################
############################################################


############################################################
############################################################
############################################################
############################################################
############################################################
############################################################
#####         Synthetic Data Simulation      ###############
############################################################
############################################################
############################################################
############################################################
############################################################
############################################################

ls()

rm(list=ls())

library(Rlab)

library(psych)

library(MASS)

library(Tlasso)

library(huge)

set.seed(123)

############################################################
############################################################
######               Data Simulation                 #######
############################################################
############################################################

p = 20 # Set dimenensionality
n = 100 # Set sample size

#########################################################################
##### Generate the covariance (Sigma) matrix & simulate data N(0,Sigma)
#########################################################################

# Model 1: : 2 neighbor Chain Graph

Omega = diag(p)

for (i in 1:p){
Omega[i,i-1] = 0.4
Omega[i-1,i] = 0.4
}

Sigma = solve(Omega)

R = -cov2cor(Omega)
diag(R) = 1
```

```
save(R, file = "pcor_1_p_20.RData")

# Simulate Data

x = mvrnorm(n, rep(0, p), Sigma)
x = scale(x, center = TRUE, scale = F)
save(x, file = "sample_1_p_20_n_100.RData")

############################################################

# Model 2: : 3 neighbor Chain Graph

Omega = diag(p)

for (i in 1:p){
Omega[i,i-1] = 0.4
Omega[i-1,i] = 0.4
}

for (i in 2:p){
Omega[i,i-2] = 0.2
Omega[i-2,i] = 0.2
}

Sigma = solve(Omega)

R = -cov2cor(Omega)
diag(R) = 1

save(R, file = "pcor_2_p_20.RData")

# Simulate Data

x = mvrnorm(n, rep(0, p), Sigma)
x = scale(x, center = TRUE, scale = F)
save(x, file = "sample_2_p_20_n_100.RData")

############################################################

# Model 3: : 2 nearest-neighbor graph

Omega = NeighborOmega(p, sd = 1, knn = 2, norm.type = 1)

Sigma = solve(Omega)

R = -cov2cor(Omega)
diag(R) = 1

save(R, file = "pcor_3_p_20.RData")

# Simulate Data

x = mvrnorm(n, rep(0, p), Sigma)
x = scale(x, center = TRUE, scale = F)
save(x, file = "sample_3_p_20_n_100.RData")

############################################################

# Model 4: Random graph

Omega = huge.generator(10000, p, graph = "random",prob=0.1)$omega # if p = 20
Omega = huge.generator(10000, p, graph = "random",prob=0.01)$omega # if p = 60
Omega = huge.generator(10000, p, graph = "random",prob=0.001)$omega # if p = 200
```

```
Omega = cov2cor(Omega)

for (i in 1:p){
Omega[i,] = ifelse(abs(Omega[i,])<=1e-6,0,Omega[i,])
}

Sigma = solve(Omega)

R = -cov2cor(Omega)
diag(R) = 1

save(R, file = "pcor_4_p_20.RData")

# Simulate Data

x = mvrnorm(n, rep(0, p), Sigma)
x = scale(x, center = TRUE, scale = F)
save(x, file = "sample_4_p_20_n_100.RData")


############################################################
############################################################
############################################################
############################################################
############################################################
############################################################
############################################################
#######     GGM Estimation p smaller than 100  #############
############################################################
############################################################
############################################################
############################################################
############################################################
############################################################

ls()

rm(list=ls())

library(huge)

library(glasso)

library(glmnet)

library(space)

set.seed(123)

############################################################
##### Functions
############################################################

###########################################################
###########################################################
###########################################################
##### GGM Estimation using PCS
###########################################################
###########################################################
###########################################################

# Function that estimates the Regression Weights and Omega using Partial
Correlation Screening (PCS)
```

```
# the input is the data matrix, the estimated partial correlation matrix and the threshold

PCS_GGM = function(x,R,tau){

x = scale(x)

n = dim(x)[1]

p = dim(x)[2]

Edges_I = combn(1:p,2) # Inactive set of ordered pair (i,j)

# Set of Edges

# Model 2 and 5

for (i in 1:p){R[i,] = ifelse(abs(R[i,])<=tau,0,R[i,])}

for (t in 1:ncol(Edges_I)){
i = Edges_I[1,t]
j = Edges_I[2,t]
if(R[i,j]==0){Edges_I[,t]=c(0,0)}
}

####

# Compute Prediction Errors and betas

beta = matrix(0,p,p)

for (i in 1:p){

n_i = c(Edges_I[1,which(Edges_I[2,]==i)],
Edges_I[2,which(Edges_I[1,]==i)])

if (length(n_i)>0){beta[n_i,i] = coef(lm(x[,i] ~ 0 + x[,n_i]))}
}

vareps = x - x%*%beta

# Compute the precision matrix

Omega = matrix(0,p,p)

diag(Omega) = apply(vareps,2,var)^(-1)

for (e in which(Edges_I[2,]>0)){
  i = Edges_I[1,e]
  j = Edges_I[2,e]

  Omega[i,j] = cov(vareps[,i],vareps[,j])*Omega[i,i]*Omega[j,j]
  Omega[j,i] = Omega[i,j]
}

# Make Omega Positive Definite

lambda_min = eigen(Omega)$values[p]
if (lambda_min < 1e-6){Omega = Omega+(0.1+abs(lambda_min))*diag(p)}

######

return(list(beta,Omega))}
```

```
###########################################################
###########################################################

# Function that estimates the Regression Weights

Psi_Screen_Beta = function(x,R,tau){

x = scale(x)

n = dim(x)[1]

p = dim(x)[2]

Edges_I = combn(1:p,2) # Inactive set of ordered pair (i,j)

# Set of Edges

# Model 2 and 5

for (i in 1:p){R[i,] = ifelse(abs(R[i,])<=tau,0,R[i,])}

for (t in 1:ncol(Edges_I)){
i = Edges_I[1,t]
j = Edges_I[2,t]
if(R[i,j]==0){Edges_I[,t]=c(0,0)}
}

# Compute Neighborhood

nei_i = function(i,R){
ne_i = which(abs(R[i,])>0)
ne_i = ne_i[-which(ne_i==i)]
return(ne_i)}

Nei = lapply(1:p, function(i) nei_i(i,R))

####

# Compute Prediction Errors and betas

beta_i = function(x,i,ne_i){
b_i = rep(0,ncol(x))
if (length(ne_i)>0){b_i[ne_i] = coef(lm(x[,i] ~ 0 + x[,ne_i]))}
return(b_i)}

beta_list = lapply(1:p, function(i) beta_i(x,i,Nei[[i]]))

beta = t(matrix(unlist(beta_list), ncol = p, byrow = TRUE))

######

return(list(beta))}

###########################################################
###########################################################
###########################################################
##### SPACE
###########################################################
###########################################################
###########################################################

# SPACE

# Function that estimates the Partial Correlation Matrix using SPACE
```

```
Psi.space = function(x,lambda.opt){

#Initialization

x = scale(x)

n = dim(x)[1]

p = dim(x)[2]

Edges_I = combn(1:p,2) # Inactive set of ordered pair (i,j)

vareps = x  # (n x p) matrix of regression residuals

fit = space.joint(x, lam1=lambda.opt, lam2=0, weight=NULL, iter=3)

R = fit[[1]]

return(R)}

###########################################################
###########################################################
###########################################################
###########################################################

# SPACE + CV

# Function that performs k-fold cross validation on SPACE

cv_space = function(x, fold){

x = scale(x)

  cv.part = function(n, k) {
    ntest = floor(n/k)
    ntrain = n - ntest
    ind = sample(n)
    trainMat = matrix(NA, nrow=ntrain, ncol=k)
    testMat = matrix(NA, nrow=ntest, ncol=k)
    nn = 1:n
    for (j in 1:k) {
      sel = ((j-1)*ntest+1):(j*ntest)
      testMat[,j] = ind[sel ]
      sel2 =nn[ !(nn %in% sel) ]
      trainMat[,j] = ind[sel2]
    }
    return(list(trainMat=trainMat,testMat=testMat))
  }

  loss.cv = function(x.train,x.test,lamlist){
    vareps = lapply(1:length(lamlist),
    function(i) space.joint(x.train, lam1=lamlist[[i]], lam2=0, weight=NULL,
iter=3)[[1]])

    eps = lapply(1:length(lamlist),function(i)
Psi_Screen_Beta(x.train,vareps[[i]],0)[[1]])

    loss.re = unlist(lapply(1:length(lamlist), function(i)
    sum(colSums((x.test - x.test%*%eps[[i]])^2))))
  return(loss.re)
  }

  n = nrow(x)
```

```
  p = ncol(x)
  part.list = cv.part(n, fold)

  l_max = sqrt(n)*qnorm(1-0.0001/(2*p^2))
  l_min = sqrt(n)*qnorm(1-0.9/(2*p^2))

  lamlist = seq(l_min,l_max,length=100)

  loss.list = lapply(1:fold, function(k)
  loss.cv(x[part.list$trainMat[, k], ],x[part.list$testMat[, k], ],lamlist))

  loss.re = matrix(unlist(loss.list), ncol = fold, byrow = FALSE)

  loss.mean = apply(loss.re, 1, mean)
  std.error = apply(loss.re, 1, sd)/sqrt(fold)
  ind = which.min(loss.mean)

  loss.mean.ls = loss.mean[ind:length(loss.mean)]
  alpha.list.ls = lamlist[ind:length(loss.mean)]

  loss.mean.ls.max = loss.mean.ls[loss.mean.ls <= loss.mean[ind] +
std.error[ind]]
  alpha.list.ls.max = alpha.list.ls[loss.mean.ls <= loss.mean[ind] +
std.error[ind]]
  ind.ls = length(loss.mean.ls.max)

  alpha_opt = lamlist[ind]
  alpha_ls1 = alpha.list.ls.max[ind.ls]

  res = list(alpha_opt=alpha_opt,alpha_ls1=alpha_ls1)
  return(res)
}

############################################################
############################################################

# SPACE + BIC

# Function that performs BIC on SPACE

BIC_space = function(x){

x = scale(x)

n = nrow(x)
p = ncol(x)

l_max = sqrt(n)*qnorm(1-0.0001/(2*p^2))

l_min = sqrt(n)*qnorm(1-0.9/(2*p^2))

lamlist = seq(l_min,l_max,length=100)

loss.re = rep(0,length(lamlist))

vareps = lapply(1:length(lamlist),function(i)
space.joint(x, lam1=lamlist[[i]], lam2=0, weight=NULL, iter=3)[[1]])

eps = lapply(1:length(lamlist),function(i) PCS_GGM(x,vareps[[i]],0)[[1]])

for (k in 1:length(lamlist)){
beta = eps[[k]]
BIC = rep(0,p)
for (i in 1:p){
```

```
BIC[i] = n*log(sum((x[,i] - x[,-i]%*%beta[i,-i])^2)) + log(n)*sum(abs(beta[i,-
i])>0)
}
loss.re[k] = sum(BIC)
}

ind = which.min(loss.re)
lamlist_opt = lamlist[ind]

res = lamlist_opt
return(res)
}


###########################################################
###########################################################
###########################################################
##### Glasso
###########################################################
###########################################################
###########################################################

# Glasso + k-fold Cross-validation
# Glasso + k-fold Cross-validation using equation (5)

cv_glasso = function(x,fold){

  cv.part = function(n, k) {
    ntest = floor(n/k)
    ntrain = n - ntest
    ind = sample(n)
    trainMat = matrix(NA, nrow=ntrain, ncol=k)
    testMat = matrix(NA, nrow=ntest, ncol=k)
    nn = 1:n
    for (j in 1:k) {
      sel = ((j-1)*ntest+1):(j*ntest)
      testMat[,j] = ind[sel ]
      sel2 = nn[ !(nn %in% sel) ]
      trainMat[,j] = ind[sel2]
    }
    return(list(trainMat=trainMat,testMat=testMat))
  }

  loss_likelihood = function(Sigma, Omega){
    tmp = (sum(diag(Sigma%*%Omega)) - log(det(Omega)) - dim(Omega)[1])
    if(is.finite(tmp)) return(tmp)
    else tmp = Inf
    return(tmp)
  }

  beta_mat = function(Omega){
    p = ncol(Omega)
    beta = matrix(0,p,p)
    for (i in 1:p){beta[,i] = -Omega[,i]/Omega[i,i]}
    diag(beta) = 0
    return(beta)
  }

  loss.cv = function(x.train,x.test,rholist){
    Omegalist = huge(x.train,lambda = rholist ,scr = F, method =
"glasso",nlambda=100)$icov
    loss.re = unlist(lapply(1:100, function(i)
loss_likelihood(cov(x.test),Omegalist[[i]])))
    loss2.re = unlist(lapply(1:100, function(i) sum(colSums((x.test - x.test%*
```

```
%beta_mat(Omegalist[[i]]))^2))))
  return(list(loss.re,loss2.re))
  }

  n = nrow(x)
  part.list = cv.part(n, fold)

  rholist = seq(0.001,max(abs(cov(x))),length=100)

  loss.list = lapply(1:fold, function(k)
  loss.cv(x[part.list$trainMat[, k], ],x[part.list$testMat[, k], ],rholist))

  loss.re = matrix(unlist(lapply(1:fold, function(k) loss.list[[k]][[1]])),ncol
= fold, byrow = FALSE)
  loss2.re = matrix(unlist(lapply(1:fold, function(k) loss.list[[k]][[2]])),ncol
= fold, byrow = FALSE)

  loss.mean = apply(loss.re, 1, mean)
  std.error = apply(loss.re, 1, sd)/sqrt(fold)
  ind = which.min(loss.mean)

  loss.mean.ls = loss.mean[ind:length(loss.mean)]
  rho.list.ls = rholist[ind:length(loss.mean)]

  loss.mean.ls.max = loss.mean.ls[loss.mean.ls <= loss.mean[ind] +
std.error[ind]]
  rho.list.ls.max = rho.list.ls[loss.mean.ls <= loss.mean[ind] + std.error[ind]]
  ind.ls = length(loss.mean.ls.max)

  rho_opt = rholist[ind]
  rho_ls1 = rho.list.ls.max[ind.ls]

  ###

  loss2.mean = apply(loss2.re, 1, mean)
  std2.error = apply(loss2.re, 1, sd)/sqrt(fold)
  ind2 = which.min(loss2.mean)

  loss2.mean.ls = loss2.mean[ind2:length(loss2.mean)]
  rho2.list.ls = rholist[ind2:length(loss2.mean)]

  loss2.mean.ls.max = loss2.mean.ls[loss2.mean.ls <= loss2.mean[ind2] +
std2.error[ind2]]
  rho2.list.ls.max = rho2.list.ls[loss2.mean.ls <= loss2.mean[ind2] +
std2.error[ind2]]
  ind2.ls = length(loss2.mean.ls.max)

  rho2_opt = rholist[ind2]
  rho2_ls1 = rho2.list.ls.max[ind2.ls]

  res =
list(rho_opt=rho_opt,rho_ls1=rho_ls1,rho2_opt=rho2_opt,rho2_ls1=rho2_ls1)
  return(res)
}


###########################################################
###########################################################
###########################################################
###########################################################
###########################################################

# Glasso + BIC and Glasso + EBIC directly on the code using huge.select
```

```
##########################################################
##########################################################
##########################################################
##### Nodewise Regression 'AND' Rule
##########################################################
##########################################################
##########################################################

# Estimate the GGM using Nodewise regression + AND rule

neigh.and.lambda = function(x,lambda){

x = scale(x)

p = ncol(x)

n = nrow(x)

Omega = matrix(0,p,p)

for (i in 1:p){
Omega[i,-i] = as.matrix(glmnet(x[,-i], x[,i], family='gaussian', lambda =
lambda[i])$beta)
}

for (i in 1:p){
for (j in 1:p){
Omega[i,j] = min(abs(Omega[i,j]),abs(Omega[j,i]))
Omega[j,i] = Omega[i,j]
}}

# Compute the precision matrix

Edges_I = combn(1:p,2) # Inactive set of ordered pair (i,j)

# Candidate Set of Edges

for (t in 1:ncol(Edges_I)){
i = Edges_I[1,t]
j = Edges_I[2,t]
if(Omega[i,j]==0){Edges_I[,t]=c(0,0)}
}

# Compute betas

beta = matrix(0,p,p)

for (i in 1:p){

n_i = c(Edges_I[1,which(Edges_I[2,]==i)],
Edges_I[2,which(Edges_I[1,]==i)])

if (length(n_i)>0){beta[n_i,i] = coef(lm(x[,i] ~ 0 + x[,n_i]))}
}

vareps = x - x%*%beta

# Estimate Precision Matrix

Omega.hat = matrix(0,p,p)

diag(Omega.hat) = apply(vareps,2,var)^(-1)

for (e in which(Edges_I[2,]>0)){
```

```
  i = Edges_I[1,e]
  j = Edges_I[2,e]
  Omega.hat[i,j] = cov(vareps[,i],vareps[,j])*Omega.hat[i,i]*Omega.hat[j,j]
  Omega.hat[j,i] = Omega.hat[i,j]
}

# Make Omega Positive Definite

lambda_min = eigen(Omega.hat)$values[p]
if (lambda_min < 1e-6){Omega.hat = Omega.hat+(0.1+abs(lambda_min))*diag(p)}

######

return(list(beta,Omega.hat))}


############################################################
############################################################

# Nodewise Regression + CV
# Function that performs k-fold cross validation on Nodewise regression

cv_Neigh = function(x, fold){

x = scale(x)

p = ncol(x)

lambda.fit = lapply(1:p, function(i) cv.glmnet(x[,-i], x[,i], family='gaussian',
nfolds=fold))

lambda.opt = unlist(lambda.fit)

lambda.opt = rep(0,p)
lambda.lse = rep(0,p)

for (i in 1:p){
lambda.opt[i] = lambda.fit[[i]]$lambda.min
lambda.lse[i] = lambda.fit[[i]]$lambda.1se
}

######

return(list(lambda.opt,lambda.lse))}


############################################################
############################################################

# Nodewise Regression + BIC
# Function that performs BIC on Nodewise Regresion

BIC_Neigh = function(x){

x = scale(x)

n = nrow(x)
p = ncol(x)

lambda.opt = rep(0,p)

for (i in 1:p){
fit.neigh = glmnet(x[,-i], x[,i], family='gaussian',nlambda=100)
beta.list = as.matrix(fit.neigh$beta)
```

```
lambdalist = fit.neigh$lambda
BIC = rep(0,ncol(beta.list))
for (k in 1:ncol(beta.list)){
BIC[k] = n*log(sum((x[,i] - x[,-i]%*%beta.list[,k])^2)) +
log(n)*sum(abs(beta.list[,k])>0)
}
ind = which.min(BIC)
lambda.opt[i] = lambdalist[ind]
}

return(lambda.opt)
}

############################################################
############################################################
############################################################
##### Nodewise Regression 'OR' Rule
############################################################
############################################################
############################################################

# Estimate the GGM using Nodewise regression + AND rule

neigh.or.lambda = function(x,lambda){

x = scale(x)

p = ncol(x)

n = nrow(x)

Omega = matrix(0,p,p)

for (i in 1:p){
Omega[i,-i] = as.matrix(glmnet(x[,-i], x[,i], family='gaussian', lambda =
lambda[i])$beta)
}

for (i in 1:p){
for (j in 1:p){
Omega[i,j] = max(abs(Omega[i,j]),abs(Omega[j,i]))
Omega[j,i] = Omega[i,j]
}}

# Compute the precision matrix

Edges_I = combn(1:p,2) # Inactive set of ordered pair (i,j)

# Candidate Set of Edges

for (t in 1:ncol(Edges_I)){
i = Edges_I[1,t]
j = Edges_I[2,t]
if(Omega[i,j]==0){Edges_I[,t]=c(0,0)}
}

# Compute betas

beta = matrix(0,p,p)

for (i in 1:p){

n_i = c(Edges_I[1,which(Edges_I[2,]==i)],
Edges_I[2,which(Edges_I[1,]==i)])
```

```
if (length(n_i)>0){beta[n_i,i] = coef(lm(x[,i] ~ 0 + x[,n_i]))}
}

vareps = x - x%*%beta

# Estimate Precision Matrix

Omega.hat = matrix(0,p,p)

diag(Omega.hat) = apply(vareps,2,var)^(-1)

for (e in which(Edges_I[2,]>0)){
  i = Edges_I[1,e]
  j = Edges_I[2,e]

  Omega.hat[i,j] = cov(vareps[,i],vareps[,j])*Omega.hat[i,i]*Omega.hat[j,j]
  Omega.hat[j,i] = Omega.hat[i,j]
}

# Make Omega Positive Definite

lambda_min = eigen(Omega.hat)$values[p]
if (lambda_min < 1e-6){Omega.hat = Omega.hat+(0.1+abs(lambda_min))*diag(p)}

######

return(list(beta,Omega.hat))}


###########################################################
###########################################################
###########################################################
##### Ridge Regression
###########################################################
###########################################################
###########################################################

# Ridge regression

ridge.lambda = function(x,lambda){

x = scale(x)

p = ncol(x)

n = nrow(x)

# Compute betas

beta = matrix(0,p,p)

for (i in 1:p){
beta[-i,i] = as.matrix(glmnet(x[,-i], x[,i], family='gaussian', alpha = 0,
lambda = lambda[i])$beta)
}

vareps = x - x%*%beta

# Set of edges

Edges_I = combn(1:p,2) # Inactive set of ordered pair (i,j)

# Estimate Precision Matrix
```

```
Omega.hat = matrix(0,p,p)

diag(Omega.hat) = apply(vareps,2,var)^(-1)

for (e in 1:ncol(Edges_I)){
  i = Edges_I[1,e]
  j = Edges_I[2,e]

  Omega.hat[i,j] = cov(vareps[,i],vareps[,j])*Omega.hat[i,i]*Omega.hat[j,j]
  Omega.hat[j,i] = Omega.hat[i,j]
}

# Make Omega Positive Definite

lambda_min = eigen(Omega.hat)$values[p]
if (lambda_min < 1e-6){Omega.hat = Omega.hat+(0.1+abs(lambda_min))*diag(p)}

######

return(list(beta,Omega.hat))}

###########################################################
###########################################################

# Function that performs k-fold cross validation on Ridge regression

cv_ridge = function(x, fold){

x = scale(x)

p = ncol(x)

lambda.opt = lapply(1:p, function(i) cv.glmnet(x[,-i], x[,i],
family='gaussian',alpha = 0, nfolds=fold)$lambda.min)

lambda.opt = unlist(lambda.opt)

######

return(lambda.opt)}

###########################################################
###########################################################
###########################################################
##### K-fols CV for PCS
###########################################################
###########################################################
###########################################################

# k-folds CV for PCS-SPACE

cv_PCS_space = function(x,lambda.opt,fold){

x = scale(x)

  cv.part = function(n, k) {
    ntest = floor(n/k)
    ntrain = n - ntest
    ind = sample(n)
    trainMat = matrix(NA, nrow=ntrain, ncol=k)
    testMat = matrix(NA, nrow=ntest, ncol=k)
    nn = 1:n
    for (j in 1:k) {
```

```
      sel = ((j-1)*ntest+1):(j*ntest)
      testMat[,j] = ind[sel ]
      sel2 =nn[ !(nn %in% sel) ]
      trainMat[,j] = ind[sel2]
    }
    return(list(trainMat=trainMat,testMat=testMat))
  }

 loss.cv = function(x.train,x.test,lambda.opt,taulist){
  R = Psi.space(x.train,lambda.opt)
  eps = lapply(1:length(taulist),function(i)
Psi_Screen_Beta(x.train,R,taulist[i])[[1]])
  loss.re = unlist(lapply(1:length(taulist), function(i) sum(colSums((x.test -
x.test%*%eps[[i]])^2))))
  return(loss.re)
 }

  n = nrow(x)
  p = ncol(x)
  part.list = cv.part(n, fold)

  taulist = seq(0.0001,1,length=100)

  loss.list = lapply(1:fold, function(k)
  loss.cv(x[part.list$trainMat[, k], ],x[part.list$testMat[,
k], ],lambda.opt,taulist))

  loss.re = matrix(unlist(loss.list), ncol = fold, byrow = FALSE)

  loss.mean = apply(loss.re, 1, mean)
  ind = which.min(loss.mean)
  tau_opt = taulist[ind]

  res = tau_opt
  return(res)
}

###########################################################
###########################################################
###########################################################

# k-folds CV for PCS: Nodewise regression 'AND'

cv_PCS_nei_and = function(x,lambda.opt,fold){

x = scale(x)

  cv.part = function(n, k) {
    ntest = floor(n/k)
    ntrain = n - ntest
    ind = sample(n)
    trainMat = matrix(NA, nrow=ntrain, ncol=k)
    testMat = matrix(NA, nrow=ntest, ncol=k)
    nn = 1:n
    for (j in 1:k) {
      sel = ((j-1)*ntest+1):(j*ntest)
      testMat[,j] = ind[sel ]
      sel2 =nn[ !(nn %in% sel) ]
      trainMat[,j] = ind[sel2]
    }
    return(list(trainMat=trainMat,testMat=testMat))
  }

 loss.cv = function(x.train,x.test,lambda.opt,taulist){
```

```
  list.nei.alpha = neigh.and.lambda(x.train,lambda.opt)
  R = -cov2cor(list.nei.alpha[[2]])
  diag(R) = 1
  eps = lapply(1:length(taulist),function(i)
Psi_Screen_Beta(x.train,R,taulist[i])[[1]])
  loss.re = unlist(lapply(1:length(taulist), function(i) sum(colSums((x.test -
x.test%*%eps[[i]])^2))))
  return(loss.re)
 }

  n = nrow(x)
  p = ncol(x)
  part.list = cv.part(n, fold)

  taulist = seq(0.0001,1,length=100)

  loss.list = lapply(1:fold, function(k)
  loss.cv(x[part.list$trainMat[, k], ],x[part.list$testMat[,
k], ],lambda.opt,taulist))

  loss.re = matrix(unlist(loss.list), ncol = fold, byrow = FALSE)

  loss.mean = apply(loss.re, 1, mean)
  ind = which.min(loss.mean)
  tau_opt = taulist[ind]

  res = tau_opt
  return(res)
}

###########################################################
###########################################################
###########################################################

# k-folds CV for PCS: Nodewise regression 'OR'

cv_PCS_nei_or = function(x,lambda.opt,fold){

x = scale(x)

  cv.part = function(n, k) {
    ntest = floor(n/k)
    ntrain = n - ntest
    ind = sample(n)
    trainMat = matrix(NA, nrow=ntrain, ncol=k)
    testMat = matrix(NA, nrow=ntest, ncol=k)
    nn = 1:n
    for (j in 1:k) {
      sel = ((j-1)*ntest+1):(j*ntest)
      testMat[,j] = ind[sel ]
      sel2 =nn[ !(nn %in% sel) ]
      trainMat[,j] = ind[sel2]
    }
    return(list(trainMat=trainMat,testMat=testMat))
  }

 loss.cv = function(x.train,x.test,lambda.opt,taulist){
  list.nei.alpha = neigh.or.lambda(x.train,lambda.opt)
  R = -cov2cor(list.nei.alpha[[2]])
  diag(R) = 1
  eps = lapply(1:length(taulist),function(i)
Psi_Screen_Beta(x.train,R,taulist[i])[[1]])
  loss.re = unlist(lapply(1:length(taulist), function(i) sum(colSums((x.test -
x.test%*%eps[[i]])^2))))
```

```
  return(loss.re)
 }

  n = nrow(x)
  p = ncol(x)
  part.list = cv.part(n, fold)

  taulist = seq(0.0001,1,length=100)

  loss.list = lapply(1:fold, function(k)
  loss.cv(x[part.list$trainMat[, k], ],x[part.list$testMat[,
k], ],lambda.opt,taulist))

  loss.re = matrix(unlist(loss.list), ncol = fold, byrow = FALSE)

  loss.mean = apply(loss.re, 1, mean)
  ind = which.min(loss.mean)
  tau_opt = taulist[ind]

  res = tau_opt
  return(res)
}


###########################################################
###########################################################
###########################################################

# k-folds CV for PCS: Glasso

cv_PCS_glasso = function(x,lambda.opt,fold){

  cv.part = function(n, k) {
    ntest = floor(n/k)
    ntrain = n - ntest
    ind = sample(n)
    trainMat = matrix(NA, nrow=ntrain, ncol=k)
    testMat = matrix(NA, nrow=ntest, ncol=k)
    nn = 1:n
    for (j in 1:k) {
      sel = ((j-1)*ntest+1):(j*ntest)
      testMat[,j] = ind[sel ]
      sel2 =nn[ !(nn %in% sel) ]
      trainMat[,j] = ind[sel2]
    }
    return(list(trainMat=trainMat,testMat=testMat))
  }

 loss.cv = function(x.train,x.test,lambda.opt,taulist){
  Omegalist = as.matrix(huge(x.train,lambda = lambda.opt ,scr = F, method =
"glasso")$icov[[1]])
  R = -cov2cor(Omegalist)
  diag(R) = 1
  eps = lapply(1:length(taulist),function(i)
Psi_Screen_Beta(x.train,R,taulist[i])[[1]])
  loss.re = unlist(lapply(1:length(taulist), function(i) sum(colSums((x.test -
x.test%*%eps[[i]])^2))))
  return(loss.re)
 }

  n = nrow(x)
  p = ncol(x)
  part.list = cv.part(n, fold)
```

```
  taulist = seq(0.0001,1,length=100)

  loss.list = lapply(1:fold, function(k)
  loss.cv(x[part.list$trainMat[, k], ],x[part.list$testMat[,
k], ],lambda.opt,taulist))

  loss.re = matrix(unlist(loss.list), ncol = fold, byrow = FALSE)

  loss.mean = apply(loss.re, 1, mean)
  ind = which.min(loss.mean)
  tau_opt = taulist[ind]

  res = tau_opt
  return(res)
}

###########################################################
###########################################################
###########################################################

# k-folds CV for PCS: Ridge

cv_PCS_ridge = function(x,lambda.opt,fold){

x = scale(x)

  cv.part = function(n, k) {
    ntest = floor(n/k)
    ntrain = n - ntest
    ind = sample(n)
    trainMat = matrix(NA, nrow=ntrain, ncol=k)
    testMat = matrix(NA, nrow=ntest, ncol=k)
    nn = 1:n
    for (j in 1:k) {
      sel = ((j-1)*ntest+1):(j*ntest)
      testMat[,j] = ind[sel ]
      sel2 =nn[ !(nn %in% sel) ]
      trainMat[,j] = ind[sel2]
    }
    return(list(trainMat=trainMat,testMat=testMat))
  }

 loss.cv = function(x.train,x.test,lambda.opt,taulist){
  list.ridge = ridge.lambda(x.train,lambda.opt)
  R = -cov2cor(list.ridge[[2]])
  diag(R) = 1
  eps = lapply(1:length(taulist),function(i)
Psi_Screen_Beta(x.train,R,taulist[i])[[1]])
  loss.re = unlist(lapply(1:length(taulist), function(i) sum(colSums((x.test -
x.test%*%eps[[i]])^2))))
  return(loss.re)
 }

  n = nrow(x)
  p = ncol(x)
  part.list = cv.part(n, fold)

  taulist = seq(0.0001,1,length=100)

  loss.list = lapply(1:fold, function(k)
  loss.cv(x[part.list$trainMat[, k], ],x[part.list$testMat[,
k], ],lambda.opt,taulist))

  loss.re = matrix(unlist(loss.list), ncol = fold, byrow = FALSE)
```

```
  loss.mean = apply(loss.re, 1, mean)
  ind = which.min(loss.mean)
  tau_opt = taulist[ind]

  res = tau_opt
  return(res)
}
###########################################################
###########################################################
###########################################################

##############################################################
##############################################################
##############################################################
##############################################################
##############################################################
##############################################################
##############################################################
##############################################################
##############################################################
##############################################################
##############################################################
##############################################################
##############################################################
##############################################################
##############################################################
##############################################################
##############################################################
##############################################################
##############################################################
##############################################################

###########################################################
###########################################################
###########################################################
##### GGM Estimation
###########################################################
###########################################################
###########################################################

# Glasso 10-folds CV1

  load(file = "sample_1_p_20_n_100.RData")
  rho.list = cv_glasso(x,fold=10)
  rho = unlist(rho.list$rho_opt)
  list.glasso.cv = as.matrix(huge(x,lambda = rho,scr = F,method = "glasso")
$icov[[1]])
  lambda.opt = rho
  save(lambda.opt, file = "lambda_glasso_cv_1_p_20_n_100.RData")
  save(list.glasso.cv, file = "glasso_cv_1_p_20_n_100.RData")

# Glasso 10-folds CV1-1se

  rho.ls1 = unlist(rho.list$rho_ls1)
  list.glasso.cv.ls1 = as.matrix(huge(x,lambda = rho.ls1,scr = F,method =
"glasso")$icov[[1]])
  lambda.opt = rho.ls1
  save(lambda.opt, file = "lambda_glasso_cv_ls1_1_p_20_n_100.RData")
  save(list.glasso.cv.ls1, file = "glasso_cv_ls1_1_p_20_n_100.RData")

# Glasso 10-folds CV2

  rho = unlist(rho.list$rho2_opt)
```

```
  list.glasso.cv2 = as.matrix(huge(x,lambda = rho,scr = F,method = "glasso")
$icov[[1]])
  lambda.opt = rho
  save(lambda.opt, file = "lambda_glasso_cv2_1_p_20_n_100.RData")
  save(list.glasso.cv2, file = "glasso_cv2_1_p_20_n_100.RData")

# Glasso 10-folds CV2-1se

  rho.ls1 = unlist(rho.list$rho2_ls1)
  list.glasso.cv2.ls1 = as.matrix(huge(x,lambda = rho.ls1,scr = F,method =
"glasso")$icov[[1]])
  lambda.opt = rho.ls1
  save(lambda.opt, file = "lambda_glasso_cv2_ls1_1_p_20_n_100.RData")
  save(list.glasso.cv2.ls1, file = "glasso_cv2_ls1_1_p_20_n_100.RData")

######################################################
######################################################
######################################################
######################################################

# Glasso EBIC

  load(file = "sample_1_p_20_n_100.RData")
  fit.ebic =
huge.select(huge(x,lambda=seq(0.001,max(abs(cov(x))),length=100),method =
"glasso"),
  ebic.gamma = 0.5,criterion = "ebic")
  lambda.opt = fit.ebic$opt.lambda
  list.glasso.ebic = as.matrix(fit.ebic$opt.icov)
  save(lambda.opt, file = "lambda_glasso_ebic_1_p_20_n_100.RData")
  save(list.glasso.ebic, file = "glasso_ebic_1_p_20_n_100.RData")

######################################################
######################################################
######################################################
######################################################

# Glasso BIC

  load(file = "sample_1_p_20_n_100.RData")
  fit.bic =
huge.select(huge(x,lambda=seq(0.001,max(abs(cov(x))),length=100),method =
"glasso"),
  ebic.gamma = 0,criterion = "ebic")
  lambda.opt = fit.bic$opt.lambda
  list.glasso.bic = as.matrix(fit.bic$opt.icov)
  save(lambda.opt, file = "lambda_glasso_bic_1_p_20_n_100.RData")
  save(list.glasso.bic, file = "glasso_bic_1_p_20_n_100.RData")

######################################################
######################################################
######################################################
######################################################

# SPACE with 10-folds CV

  load(file = "sample_1_p_20_n_100.RData")
  lambda.list = cv_space(x,fold=10)
  lambda.opt = lambda.list$alpha_opt
  R.opt = Psi.space(x,lambda.opt)
  list.space.cv = PCS_GGM(x,R.opt,0)
  save(lambda.opt, file = "lambda_space_cv_1_p_20_n_100.RData")
  save(list.space.cv, file = "space_cv_1_p_20_n_100.RData")
```

```
  lambda.ls1 = lambda.list$alpha_ls1
  R.opt = Psi.space(x,lambda.ls1)
  list.space.cv.ls1 = PCS_GGM(x,R.opt,0)
  lambda.opt = lambda.ls1
  save(lambda.opt, file = "lambda_space_cv_ls1_1_p_20_n_100.RData")
  save(list.space.cv.ls1, file = "space_cv_ls1_1_p_20_n_100.RData")

######################################################
######################################################
######################################################
######################################################

# SPACE with finite sample results result

  load(file = "sample_1_p_20_n_100.RData")
  lambda.opt = sqrt(nrow(x))*qnorm(1-0.05/(2*ncol(x)^2))
  R.opt = Psi.space(x,lambda.opt)
  list.space.alpha = PCS_GGM(x,R.opt,0)
  save(lambda.opt, file = "lambda_space_alpha_1_p_20_n_100.RData")
  save(list.space.alpha, file = "space_alpha_1_p_20_n_100.RData")

######################################################
######################################################
######################################################
######################################################

# SPACE with BIC

  load(file = "sample_1_p_20_n_100.RData")
  lambda.opt = BIC_space(x)
  R.opt = Psi.space(x,lambda.opt)
  list.space.bic = PCS_GGM(x,R.opt,0)
  save(lambda.opt, file = "lambda_space_bic_1_p_20_n_100.RData")
  save(list.space.bic, file = "space_bic_1_p_20_n_100.RData")

######################################################
######################################################
######################################################
######################################################

# Nodewise regression 'AND' with 10-folds CV

  load(file = "sample_1_p_20_n_100.RData")
  lambda.fit = cv_Neigh(x,10)
  lambda.opt = lambda.fit[[1]]
  list.nei.cv = neigh.and.lambda(x,lambda.opt)
  save(lambda.opt, file = "lambda_nei_and_cv_1_p_20_n_100.RData")
  save(list.nei.cv, file = "nei_and_cv_1_p_20_n_100.RData")

# Nodewise regression 'AND' with 10-folds CV-ls1

  list.nei.cv = neigh.or.lambda(x,lambda.opt)
  save(lambda.opt, file = "lambda_nei_or_cv_1_p_20_n_100.RData")
  save(list.nei.cv, file = "nei_or_cv_1_p_20_n_100.RData")

# Nodewise regression 'OR' with 10-folds CV

  lambda.ls1 = lambda.fit[[2]]
  list.nei.cv.ls1 = neigh.and.lambda(x,lambda.ls1)
  lambda.opt = lambda.ls1
  save(lambda.opt, file = "lambda_nei_and_cv_ls1_1_p_20_n_100.RData")
  save(list.nei.cv.ls1, file = "nei_and_cv_ls1_1_p_20_n_100.RData")

# Nodewise regression 'OR' with 10-folds CV-ls1
```

```
  list.nei.cv.ls1 = neigh.or.lambda(x,lambda.ls1)
  save(lambda.opt, file = "lambda_nei_or_cv_ls1_1_p_20_n_100.RData")
  save(list.nei.cv.ls1, file = "nei_or_cv_ls1_1_p_20_n_100.RData")

######################################################
######################################################
######################################################
######################################################

# Nodewise regression 'AND' with finite sample result

  load(file = "sample_1_p_20_n_100.RData")
  lambda.opt = rep((1/(sqrt(nrow(x))))*qnorm(1-0.05/(2*ncol(x)^2)),ncol(x))
  list.nei.alpha = neigh.and.lambda(x,lambda.opt)
  save(lambda.opt, file = "lambda_nei_and_alpha_1_p_20_n_100.RData")
  save(list.nei.alpha, file = "nei_and_alpha_1_p_20_n_100.RData")

# Nodewise regression 'OR' with finite sample result

  list.nei.alpha = neigh.or.lambda(x,lambda.opt)
  save(lambda.opt, file = "lambda_nei_or_alpha_1_p_20_n_100.RData")
  save(list.nei.alpha, file = "nei_or_alpha_1_p_20_n_100.RData")

######################################################
######################################################
######################################################
######################################################

# Nodewise regression 'AND' with BIC

  load(file = "sample_1_p_20_n_100.RData")
  lambda.opt = BIC_Neigh(x)
  list.nei.bic = neigh.and.lambda(x,lambda.opt)
  save(lambda.opt, file = "lambda_nei_and_bic_1_p_20_n_100.RData")
  save(list.nei.bic, file = "nei_and_bic_1_p_20_n_100.RData")

# Nodewise regression 'OR' with BIC

  list.nei.bic = neigh.or.lambda(x,lambda.opt)
  save(lambda.opt, file = "lambda_nei_or_bic_1_p_20_n_100.RData")
  save(list.nei.bic, file = "nei_or_bic_1_p_20_n_100.RData")

######################################################
######################################################
######################################################
######################################################

# Ridge regression with 10-folds CV

  load(file = "sample_1_p_20_n_100.RData")
  lambda.opt = cv_ridge(x,10)
  list.ridge.cv = ridge.lambda(x,lambda.opt)
  save(lambda.opt, file = "lambda_ridge_cv_1_p_20_n_100.RData")
  save(list.ridge.cv, file = "ridge_cv_1_p_20_n_100.RData")

######################################################
######################################################
######################################################
######################################################
######################################################
######################################################
######################################################
######################################################
```

```
############################################################
############################################################
############################################################
##### GGM Estimation with PCS
############################################################
############################################################
############################################################

# Glasso 10-folds CV1

  load(file = "sample_1_p_20_n_100.RData")
  load(file = "glasso_cv_1_p_20_n_100.RData")
  load(file = "lambda_glasso_cv_1_p_20_n_100.RData")
  R.opt = -cov2cor(list.glasso.cv)
  diag(R.opt) = 1
  tau.opt = cv_PCS_glasso(x,lambda.opt,fold=10)
  list.glasso.cv.pcs = PCS_GGM(x,R.opt,tau.opt)
  save(tau.opt, file = "tau_glasso_cv_1_p_20_n_100.RData")
  save(list.glasso.cv.pcs, file = "PCS_glasso_cv_1_p_20_n_100.RData")

# Glasso 10-folds CV1-1se

  load(file = "sample_1_p_20_n_100.RData")
  load(file = "glasso_cv_ls1_1_p_20_n_100.RData")
  load(file = "lambda_glasso_cv_ls1_1_p_20_n_100.RData")
  R.opt = -cov2cor(list.glasso.cv.ls1)
  diag(R.opt) = 1
  tau.opt = cv_PCS_glasso(x,lambda.opt,fold=10)
  list.glasso.cv.ls1.pcs = PCS_GGM(x,R.opt,tau.opt)
  save(tau.opt, file = "tau_glasso_cv_ls1_1_p_20_n_100.RData")
  save(list.glasso.cv.ls1.pcs, file = "PCS_glasso_cv_ls1_1_p_20_n_100.RData")

# Glasso 10-folds CV2

  load(file = "sample_1_p_20_n_100.RData")
  load(file = "glasso_cv2_1_p_20_n_100.RData")
  load(file = "lambda_glasso_cv2_1_p_20_n_100.RData")
  R.opt = -cov2cor(list.glasso.cv2)
  diag(R.opt) = 1
  tau.opt = cv_PCS_glasso(x,lambda.opt,fold=10)
  list.glasso.cv2.pcs = PCS_GGM(x,R.opt,tau.opt)
  save(tau.opt, file = "tau_glasso_cv2_1_p_20_n_100.RData")
  save(list.glasso.cv2.pcs, file = "PCS_glasso_cv2_1_p_20_n_100.RData")

# Glasso 10-folds CV2-1se

  load(file = "sample_1_p_20_n_100.RData")
  load(file = "glasso_cv2_ls1_1_p_20_n_100.RData")
  load(file = "lambda_glasso_cv2_ls1_1_p_20_n_100.RData")
  R.opt = -cov2cor(list.glasso.cv2.ls1)
  diag(R.opt) = 1
  tau.opt = cv_PCS_glasso(x,lambda.opt,fold=10)
  list.glasso.cv2.ls1.pcs = PCS_GGM(x,R.opt,tau.opt)
  save(tau.opt, file = "tau_glasso_cv2_ls1_1_p_20_n_100.RData")
  save(list.glasso.cv2.ls1.pcs, file = "PCS_glasso_cv2_ls1_1_p_20_n_100.RData")


#######################################################
#######################################################
#######################################################
#######################################################

# Glasso EBIC
```

```
  load(file = "sample_1_p_20_n_100.RData")
  load(file = "glasso_ebic_1_p_20_n_100.RData")
  load(file = "lambda_glasso_ebic_1_p_20_n_100.RData")
  R.opt = -cov2cor(list.glasso.ebic)
  diag(R.opt) = 1
  tau.opt = cv_PCS_glasso(x,lambda.opt,fold=10)
  list.glasso.ebic.pcs = PCS_GGM(x,R.opt,tau.opt)
  save(tau.opt, file = "tau_glasso_ebic_1_p_20_n_100.RData")
  save(list.glasso.ebic.pcs, file = "PCS_glasso_ebic_1_p_20_n_100.RData")


######################################################
######################################################
######################################################
######################################################

# Glasso BIC

  load(file = "sample_1_p_20_n_100.RData")
  load(file = "glasso_bic_1_p_20_n_100.RData")
  load(file = "lambda_glasso_bic_1_p_20_n_100.RData")
  R.opt = -cov2cor(list.glasso.bic)
  diag(R.opt) = 1
  tau.opt = cv_PCS_glasso(x,lambda.opt,fold=10)
  list.glasso.bic.pcs = PCS_GGM(x,R.opt,tau.opt)
  save(tau.opt, file = "tau_glasso_bic_1_p_20_n_100.RData")
  save(list.glasso.bic.pcs, file = "PCS_glasso_bic_1_p_20_n_100.RData")

######################################################
######################################################
######################################################
######################################################

# SPACE with 10-folds CV

  load(file = "sample_1_p_20_n_100.RData")
  load(file = "space_cv_1_p_20_n_100.RData")
  load(file = "lambda_space_cv_1_p_20_n_100.RData")
  R.opt = Psi.space(x,lambda.opt)
  #R.opt = -cov2cor(list.space.cv[[2]])
  #diag(R.opt) = 1
  tau.opt = cv_PCS_space(x,lambda.opt,10)
  list.space.cv.pcs = PCS_GGM(x,R.opt,tau.opt)
  save(tau.opt, file = "tau_space_cv_1_p_20_n_100.RData")
  save(list.space.cv.pcs, file = "PCS_space_cv_1_p_20_n_100.RData")

  load(file = "sample_1_p_20_n_100.RData")
  load(file = "space_cv_ls1_1_p_20_n_100.RData")
  load(file = "lambda_space_cv_ls1_1_p_20_n_100.RData")
  R.opt = Psi.space(x,lambda.opt)
  #R.opt = -cov2cor(list.space.cv.ls1[[2]])
  #diag(R.opt) = 1
  tau.opt = cv_PCS_space(x,lambda.opt,10)
  list.space.cv.ls1.pcs = PCS_GGM(x,R.opt,tau.opt)
  save(tau.opt, file = "tau_space_cv_ls1_1_p_20_n_100.RData")
  save(list.space.cv.ls1.pcs, file = "PCS_space_cv_ls1_1_p_20_n_100.RData")

######################################################
######################################################
######################################################
######################################################

# SPACE with finite sample results result
```

```
  load(file = "sample_1_p_20_n_100.RData")
  load(file = "space_alpha_1_p_20_n_100.RData")
  load(file = "lambda_space_alpha_1_p_20_n_100.RData")
  R.opt = Psi.space(x,lambda.opt)
  #R.opt = -cov2cor(list.space.alpha[[2]])
  #diag(R.opt) = 1
  tau.opt = cv_PCS_space(x,lambda.opt,10)
  list.space.alpha.pcs = PCS_GGM(x,R.opt,tau.opt)
  save(tau.opt, file = "tau_space_alpha_1_p_20_n_100.RData")
  save(list.space.alpha.pcs, file = "PCS_space_alpha_1_p_20_n_100.RData")

#######################################################
#######################################################
#######################################################
#######################################################

# SPACE with BIC

  load(file = "sample_1_p_20_n_100.RData")
  load(file = "space_bic_1_p_20_n_100.RData")
  load(file = "lambda_space_bic_1_p_20_n_100.RData")
  R.opt = Psi.space(x,lambda.opt)
  #R.opt = -cov2cor(list.space.bic[[2]])
  #diag(R.opt) = 1
  tau.opt = cv_PCS_space(x,lambda.opt,10)
  list.space.bic.pcs = PCS_GGM(x,R.opt,tau.opt)
  save(tau.opt, file = "tau_space_bic_1_p_20_n_100.RData")
  save(list.space.bic.pcs, file = "PCS_space_bic_1_p_20_n_100.RData")

#######################################################
#######################################################
#######################################################
#######################################################

# Nodewise regression 'AND' with 10-folds CV

  load(file = "sample_1_p_20_n_100.RData")
  load(file = "nei_and_cv_1_p_20_n_100.RData")
  load(file = "lambda_nei_and_cv_1_p_20_n_100.RData")
  R.opt = -cov2cor(list.nei.cv[[2]])
  diag(R.opt) = 1
  tau.opt = cv_PCS_nei_and(x,lambda.opt,10)
  list.nei.cv.pcs = PCS_GGM(x,R.opt,tau.opt)
  save(tau.opt, file = "tau_nei_and_cv_1_p_20_n_100.RData")
  save(list.nei.cv.pcs, file = "PCS_nei_and_cv_1_p_20_n_100.RData")

# Nodewise regression 'AND' with 10-folds CV-ls1

  load(file = "sample_1_p_20_n_100.RData")
  load(file = "nei_or_cv_1_p_20_n_100.RData")
  load(file = "lambda_nei_or_cv_1_p_20_n_100.RData")
  R.opt = -cov2cor(list.nei.cv[[2]])
  diag(R.opt) = 1
  tau.opt = cv_PCS_nei_or(x,lambda.opt,10)
  list.nei.cv.pcs = PCS_GGM(x,R.opt,tau.opt)
  save(tau.opt, file = "tau_nei_or_cv_1_p_20_n_100.RData")
  save(list.nei.cv.pcs, file = "PCS_nei_or_cv_1_p_20_n_100.RData")

# Nodewise regression 'OR' with 10-folds CV

  load(file = "sample_1_p_20_n_100.RData")
  load(file = "nei_and_cv_ls1_1_p_20_n_100.RData")
  load(file = "lambda_nei_and_cv_ls1_1_p_20_n_100.RData")
```

```
  R.opt = -cov2cor(list.nei.cv.ls1[[2]])
  diag(R.opt) = 1
  tau.opt = cv_PCS_nei_and(x,lambda.opt,10)
  list.nei.cv.ls1.pcs = PCS_GGM(x,R.opt,tau.opt)
  save(tau.opt, file = "tau_nei_and_cv_ls1_1_p_20_n_100.RData")
  save(list.nei.cv.ls1.pcs, file = "PCS_nei_and_cv_ls1_1_p_20_n_100.RData")

# Nodewise regression 'OR' with 10-folds CV-ls1

  load(file = "sample_1_p_20_n_100.RData")
  load(file = "nei_or_cv_ls1_1_p_20_n_100.RData")
  load(file = "lambda_nei_or_cv_ls1_1_p_20_n_100.RData")
  R.opt = -cov2cor(list.nei.cv.ls1[[2]])
  diag(R.opt) = 1
  tau.opt = cv_PCS_nei_or(x,lambda.opt,10)
  list.nei.cv.ls1.pcs = PCS_GGM(x,R.opt,tau.opt)
  save(tau.opt, file = "tau_nei_or_cv_ls1_1_p_20_n_100.RData")
  save(list.nei.cv.ls1.pcs, file = "PCS_nei_or_cv_ls1_1_p_20_n_100.RData")

######################################################
######################################################
######################################################
######################################################

# Nodewise regression 'AND' with finite sample result

  load(file = "sample_1_p_20_n_100.RData")
  load(file = "nei_and_alpha_1_p_20_n_100.RData")
  load(file = "lambda_nei_and_alpha_1_p_20_n_100.RData")
  R.opt = -cov2cor(list.nei.alpha[[2]])
  diag(R.opt) = 1
  tau.opt = cv_PCS_nei_and(x,lambda.opt,10)
  list.nei.alpha.pcs = PCS_GGM(x,R.opt,tau.opt)
  save(tau.opt, file = "tau_nei_and_alpha_1_p_20_n_100.RData")
  save(list.nei.alpha.pcs, file = "PCS_nei_and_alpha_1_p_20_n_100.RData")

# Nodewise regression 'OR' with finite sample result

  load(file = "sample_1_p_20_n_100.RData")
  load(file = "nei_or_alpha_1_p_20_n_100.RData")
  load(file = "lambda_nei_or_alpha_1_p_20_n_100.RData")
  R.opt = -cov2cor(list.nei.alpha[[2]])
  diag(R.opt) = 1
  tau.opt = cv_PCS_nei_or(x,lambda.opt,10)
  list.nei.alpha.pcs = PCS_GGM(x,R.opt,tau.opt)
  save(tau.opt, file = "tau_nei_or_alpha_1_p_20_n_100.RData")
  save(list.nei.alpha.pcs, file = "PCS_nei_or_alpha_1_p_20_n_100.RData")

######################################################
######################################################
######################################################
######################################################

# Nodewise regression 'AND' with BIC

  load(file = "sample_1_p_20_n_100.RData")
  load(file = "nei_and_bic_1_p_20_n_100.RData")
  load(file = "lambda_nei_and_bic_1_p_20_n_100.RData")
  R.opt = -cov2cor(list.nei.bic[[2]])
  diag(R.opt) = 1
  tau.opt = cv_PCS_nei_and(x,lambda.opt,10)
  list.nei.bic.pcs = PCS_GGM(x,R.opt,tau.opt)
  save(tau.opt, file = "tau_nei_and_bic_1_p_20_n_100.RData")
  save(list.nei.bic.pcs, file = "PCS_nei_and_bic_1_p_20_n_100.RData")
```

```
# Nodewise regression 'OR' with BIC

  load(file = "sample_1_p_20_n_100.RData")
  load(file = "nei_or_bic_1_p_20_n_100.RData")
  load(file = "lambda_nei_or_bic_1_p_20_n_100.RData")
  R.opt = -cov2cor(list.nei.bic[[2]])
  diag(R.opt) = 1
  tau.opt = cv_PCS_nei_or(x,lambda.opt,10)
  list.nei.bic.pcs = PCS_GGM(x,R.opt,tau.opt)
  save(tau.opt, file = "tau_nei_or_bic_1_p_20_n_100.RData")
  save(list.nei.bic.pcs, file = "PCS_nei_or_bic_1_p_20_n_100.RData")

######################################################
######################################################
######################################################
######################################################

# Ridge regression with 10-folds CV

  load(file = "sample_1_p_20_n_100.RData")
  load(file = "ridge_cv_1_p_20_n_100.RData")
  load(file = "lambda_ridge_cv_1_p_20_n_100.RData")
  R.opt = -cov2cor(list.ridge.cv[[2]])
  diag(R.opt) = 1
  tau.opt = cv_PCS_ridge(x,lambda.opt,10)
  list.ridge.cv.pcs = PCS_GGM(x,R.opt,tau.opt)
  save(tau.opt, file = "tau_ridge_cv_1_p_20_n_100.RData")
  save(list.ridge.cv.pcs, file = "PCS_ridge_cv_1_p_20_n_100.RData")

######################################################
######################################################
######################################################
######################################################
######################################################
######################################################
######################################################
######################################################


############################################################
############################################################
############################################################
############################################################
############################################################
############################################################
############################################################
#######     GGM Estimation p larger than 100  ##############
############################################################
############################################################
############################################################
############################################################
############################################################
############################################################

ls()

rm(list=ls())

library(huge)

library(glasso)

library(glmnet)
```

```
library(space)

set.seed(123)

############################################################
##### Functions
############################################################

###########################################################
###########################################################
###########################################################
##### GGM Estimation using PCS
###########################################################
###########################################################
###########################################################

# Function that estimates the Regression Weights and Omega using Partial
Correlation Screening (PCS)
# the input is the data matrix, the estimated partial correlation matrix and the
threshold

PCS_GGM = function(x,R,tau){

x = scale(x)

n = dim(x)[1]

p = dim(x)[2]

Edges_I = combn(1:p,2) # Inactive set of ordered pair (i,j)

# Set of Edges

# Model 2 and 5

for (i in 1:p){R[i,] = ifelse(abs(R[i,])<=tau,0,R[i,])}

for (t in 1:ncol(Edges_I)){
i = Edges_I[1,t]
j = Edges_I[2,t]
if(R[i,j]==0){Edges_I[,t]=c(0,0)}
}

####

# Compute Prediction Errors and betas

beta = matrix(0,p,p)

for (i in 1:p){

n_i = c(Edges_I[1,which(Edges_I[2,]==i)],
Edges_I[2,which(Edges_I[1,]==i)])

if (length(n_i)>0){beta[n_i,i] = coef(lm(x[,i] ~ 0 + x[,n_i]))}
}

vareps = x - x%*%beta

# Compute the precision matrix

Omega = matrix(0,p,p)
```

```
diag(Omega) = apply(vareps,2,var)^(-1)

for (e in which(Edges_I[2,]>0)){
  i = Edges_I[1,e]
  j = Edges_I[2,e]

  Omega[i,j] = cov(vareps[,i],vareps[,j])*Omega[i,i]*Omega[j,j]
  Omega[j,i] = Omega[i,j]
}

# Make Omega Positive Definite

lambda_min = eigen(Omega)$values[p]
if (lambda_min < 1e-6){Omega = Omega+(0.1+abs(lambda_min))*diag(p)}

######

return(list(beta,Omega))}

############################################################
############################################################

# Function that estimates the Regression Weights

Psi_Screen_Beta = function(x,R,tau){

x = scale(x)

n = dim(x)[1]

p = dim(x)[2]

Edges_I = combn(1:p,2) # Inactive set of ordered pair (i,j)

# Set of Edges

# Model 2 and 5

for (i in 1:p){R[i,] = ifelse(abs(R[i,])<=tau,0,R[i,])}

for (t in 1:ncol(Edges_I)){
i = Edges_I[1,t]
j = Edges_I[2,t]
if(R[i,j]==0){Edges_I[,t]=c(0,0)}
}

# Compute Neighborhood

nei_i = function(i,R){
ne_i = which(abs(R[i,])>0)
ne_i = ne_i[-which(ne_i==i)]
return(ne_i)}

Nei = lapply(1:p, function(i) nei_i(i,R))

####

# Compute Prediction Errors and betas

beta_i = function(x,i,ne_i){
b_i = rep(0,ncol(x))
if (length(ne_i)>0){b_i[ne_i] = coef(lm(x[,i] ~ 0 + x[,ne_i]))}
return(b_i)}
```

```
beta_list = lapply(1:p, function(i) beta_i(x,i,Nei[[i]]))

beta = t(matrix(unlist(beta_list), ncol = p, byrow = TRUE))

######

return(list(beta))}

############################################################
############################################################
############################################################
##### SPACE
############################################################
############################################################
############################################################

# SPACE

# Function that estimates the Partial Correlation Matrix using SPACE

Psi.space = function(x,lambda.opt){

#Initialization

x = scale(x)

n = dim(x)[1]

p = dim(x)[2]

Edges_I = combn(1:p,2) # Inactive set of ordered pair (i,j)

vareps = x  # (n x p) matrix of regression residuals

fit = space.joint(x, lam1=lambda.opt, lam2=0, weight=NULL, iter=3)

R = fit[[1]]

return(R)}

############################################################
############################################################
############################################################
############################################################

# SPACE + CV

# Function that performs k-fold cross validation on SPACE

cv_space = function(x, fold){

x = scale(x)

  cv.part = function(n, k) {
    ntest = floor(n/k)
    ntrain = n - ntest
    ind = sample(n)
    trainMat = matrix(NA, nrow=ntrain, ncol=k)
    testMat = matrix(NA, nrow=ntest, ncol=k)
    nn = 1:n
    for (j in 1:k) {
      sel = ((j-1)*ntest+1):(j*ntest)
      testMat[,j] = ind[sel ]
      sel2 =nn[ !(nn %in% sel) ]
```

```
      trainMat[,j] = ind[sel2]
    }
    return(list(trainMat=trainMat,testMat=testMat))
  }

  loss.cv = function(x.train,x.test,lamlist){
    vareps = lapply(1:length(lamlist),
    function(i) space.joint(x.train, lam1=lamlist[[i]], lam2=0, weight=NULL,
iter=3)[[1]])

    eps = lapply(1:length(lamlist),function(i)
Psi_Screen_Beta(x.train,vareps[[i]],0)[[1]])

    loss.re = unlist(lapply(1:length(lamlist), function(i)
    sum(colSums((x.test - x.test%*%eps[[i]])^2))))
  return(loss.re)
  }

  n = nrow(x)
  p = ncol(x)
  part.list = cv.part(n, fold)

  l_max = sqrt(n)*qnorm(1-0.0001/(2*p^2))
  l_min = sqrt(n)*qnorm(1-0.9/(2*p^2))

  lamlist = seq(l_min,l_max,length=100)

  loss.list = lapply(1:fold, function(k)
  loss.cv(x[part.list$trainMat[, k], ],x[part.list$testMat[, k], ],lamlist))

  loss.re = matrix(unlist(loss.list), ncol = fold, byrow = FALSE)

  loss.mean = apply(loss.re, 1, mean)
  std.error = apply(loss.re, 1, sd)/sqrt(fold)
  ind = which.min(loss.mean)

  loss.mean.ls = loss.mean[ind:length(loss.mean)]
  alpha.list.ls = lamlist[ind:length(loss.mean)]

  loss.mean.ls.max = loss.mean.ls[loss.mean.ls <= loss.mean[ind] +
std.error[ind]]
  alpha.list.ls.max = alpha.list.ls[loss.mean.ls <= loss.mean[ind] +
std.error[ind]]
  ind.ls = length(loss.mean.ls.max)

  alpha_opt = lamlist[ind]
  alpha_ls1 = alpha.list.ls.max[ind.ls]

  res = list(alpha_opt=alpha_opt,alpha_ls1=alpha_ls1)
  return(res)
}

############################################################
############################################################

# SPACE + BIC

# Function that performs BIC on SPACE

BIC_space = function(x){

x = scale(x)

n = nrow(x)
```

```
p = ncol(x)

l_max = sqrt(n)*qnorm(1-0.0001/(2*p^2))

l_min = sqrt(n)*qnorm(1-0.9/(2*p^2))

lamlist = seq(l_min,l_max,length=100)

loss.re = rep(0,length(lamlist))

vareps = lapply(1:length(lamlist),function(i)
space.joint(x, lam1=lamlist[[i]], lam2=0, weight=NULL, iter=3)[[1]])

eps = lapply(1:length(lamlist),function(i) PCS_GGM(x,vareps[[i]],0)[[1]])

for (k in 1:length(lamlist)){
beta = eps[[k]]
BIC = rep(0,p)
for (i in 1:p){
BIC[i] = n*log(sum((x[,i] - x[,-i]%*%beta[i,-i])^2)) + log(n)*sum(abs(beta[i,-
i])>0)
}
loss.re[k] = sum(BIC)
}

ind = which.min(loss.re)
lamlist_opt = lamlist[ind]

res = lamlist_opt
return(res)
}


############################################################
############################################################
############################################################
##### Glasso
############################################################
############################################################
############################################################

# Glasso + k-fold Cross-validation
# Glasso + k-fold Cross-validation using equation (5)

cv_glasso = function(x,fold){

  cv.part = function(n, k) {
    ntest = floor(n/k)
    ntrain = n - ntest
    ind = sample(n)
    trainMat = matrix(NA, nrow=ntrain, ncol=k)
    testMat = matrix(NA, nrow=ntest, ncol=k)
    nn = 1:n
    for (j in 1:k) {
      sel = ((j-1)*ntest+1):(j*ntest)
      testMat[,j] = ind[sel ]
      sel2 = nn[ !(nn %in% sel) ]
      trainMat[,j] = ind[sel2]
    }
    return(list(trainMat=trainMat,testMat=testMat))
  }

  loss_likelihood = function(Sigma, Omega){
    tmp = (sum(diag(Sigma%*%Omega)) - log(det(Omega)) - dim(Omega)[1])
```

```
    if(is.finite(tmp)) return(tmp)
    else tmp = Inf
    return(tmp)
  }

  beta_mat = function(Omega){
    p = ncol(Omega)
    beta = matrix(0,p,p)
    for (i in 1:p){beta[,i] = -Omega[,i]/Omega[i,i]}
    diag(beta) = 0
    return(beta)
  }

  loss.cv = function(x.train,x.test,rholist){
    Omegalist = huge(x.train,lambda = rholist ,scr = F, method = 
"glasso",nlambda=100)$icov
    loss.re = unlist(lapply(1:100, function(i) 
loss_likelihood(cov(x.test),Omegalist[[i]])))
    loss2.re = unlist(lapply(1:100, function(i) sum(colSums((x.test - x.test%*
%beta_mat(Omegalist[[i]]))^2))))
  return(list(loss.re,loss2.re))
  }

  n = nrow(x)
  part.list = cv.part(n, fold)

  rholist = seq(0.05,max(abs(cov(x))),length=100)

  loss.list = lapply(1:fold, function(k)
  loss.cv(x[part.list$trainMat[, k], ],x[part.list$testMat[, k], ],rholist))

  loss.re = matrix(unlist(lapply(1:fold, function(k) loss.list[[k]][[1]])),ncol 
= fold, byrow = FALSE)
  loss2.re = matrix(unlist(lapply(1:fold, function(k) loss.list[[k]][[2]])),ncol 
= fold, byrow = FALSE)

  loss.mean = apply(loss.re, 1, mean)
  std.error = apply(loss.re, 1, sd)/sqrt(fold)
  ind = which.min(loss.mean)

  loss.mean.ls = loss.mean[ind:length(loss.mean)]
  rho.list.ls = rholist[ind:length(loss.mean)]

  loss.mean.ls.max = loss.mean.ls[loss.mean.ls <= loss.mean[ind] + 
std.error[ind]]
  rho.list.ls.max = rho.list.ls[loss.mean.ls <= loss.mean[ind] + std.error[ind]]
  ind.ls = length(loss.mean.ls.max)

  rho_opt = rholist[ind]
  rho_ls1 = rho.list.ls.max[ind.ls]

  ###

  loss2.mean = apply(loss2.re, 1, mean)
  std2.error = apply(loss2.re, 1, sd)/sqrt(fold)
  ind2 = which.min(loss2.mean)

  loss2.mean.ls = loss2.mean[ind2:length(loss2.mean)]
  rho2.list.ls = rholist[ind2:length(loss2.mean)]

  loss2.mean.ls.max = loss2.mean.ls[loss2.mean.ls <= loss2.mean[ind2] + 
std2.error[ind2]]
  rho2.list.ls.max = rho2.list.ls[loss2.mean.ls <= loss2.mean[ind2] + 
std2.error[ind2]]
```

```
  ind2.ls = length(loss2.mean.ls.max)

  rho2_opt = rholist[ind2]
  rho2_ls1 = rho2.list.ls.max[ind2.ls]

  res =
list(rho_opt=rho_opt,rho_ls1=rho_ls1,rho2_opt=rho2_opt,rho2_ls1=rho2_ls1)
  return(res)
}


###########################################################
###########################################################
###########################################################
###########################################################
###########################################################

# Glasso + BIC and Glasso + EBIC directly on the code using huge.select

###########################################################
###########################################################
###########################################################
##### Nodewise Regression 'AND' Rule
###########################################################
###########################################################
###########################################################

# Estimate the GGM using Nodewise regression + AND rule

neigh.and.lambda = function(x,lambda){

x = scale(x)

p = ncol(x)

n = nrow(x)

Omega = matrix(0,p,p)

for (i in 1:p){
Omega[i,-i] = as.matrix(glmnet(x[,-i], x[,i], family='gaussian', lambda =
lambda[i],
lambda.min.ratio = 0.05)$beta)
}

for (i in 1:p){
for (j in 1:p){
Omega[i,j] = min(abs(Omega[i,j]),abs(Omega[j,i]))
Omega[j,i] = Omega[i,j]
}}

# Compute the precision matrix

Edges_I = combn(1:p,2) # Inactive set of ordered pair (i,j)

# Candidate Set of Edges

for (t in 1:ncol(Edges_I)){
i = Edges_I[1,t]
j = Edges_I[2,t]
if(Omega[i,j]==0){Edges_I[,t]=c(0,0)}
}

# Compute betas
```

```
beta = matrix(0,p,p)

for (i in 1:p){

n_i = c(Edges_I[1,which(Edges_I[2,]==i)],
Edges_I[2,which(Edges_I[1,]==i)])

if (length(n_i)>0){beta[n_i,i] = coef(lm(x[,i] ~ 0 + x[,n_i]))}
}

vareps = x - x%*%beta

# Estimate Precision Matrix

Omega.hat = matrix(0,p,p)

diag(Omega.hat) = apply(vareps,2,var)^(-1)

for (e in which(Edges_I[2,]>0)){
  i = Edges_I[1,e]
  j = Edges_I[2,e]
  Omega.hat[i,j] = cov(vareps[,i],vareps[,j])*Omega.hat[i,i]*Omega.hat[j,j]
  Omega.hat[j,i] = Omega.hat[i,j]
}

# Make Omega Positive Definite

lambda_min = eigen(Omega.hat)$values[p]
if (lambda_min < 1e-6){Omega.hat = Omega.hat+(0.1+abs(lambda_min))*diag(p)}

######

return(list(beta,Omega.hat))}


###########################################################
###########################################################

# Nodewise Regression + CV
# Function that performs k-fold cross validation on Nodewise regression

cv_Neigh = function(x, fold){

x = scale(x)

p = ncol(x)

lambda.fit = lapply(1:p, function(i) cv.glmnet(x[,-i], x[,i], family='gaussian',
nfolds=fold,
lambda.min.ratio = 0.05))

lambda.opt = unlist(lambda.fit)

lambda.opt = rep(0,p)
lambda.lse = rep(0,p)

for (i in 1:p){
lambda.opt[i] = lambda.fit[[i]]$lambda.min
lambda.lse[i] = lambda.fit[[i]]$lambda.1se
}

######
```

```
return(list(lambda.opt,lambda.lse))}


############################################################
############################################################

# Nodewise Regression + BIC
# Function that performs BIC on Nodewise Regresion

BIC_Neigh = function(x){

x = scale(x)

n = nrow(x)
p = ncol(x)

lambda.opt = rep(0,p)

for (i in 1:p){
fit.neigh = glmnet(x[,-i], x[,i], family='gaussian',nlambda=100,
lambda.min.ratio = 0.05)
beta.list = as.matrix(fit.neigh$beta)
lambdalist = fit.neigh$lambda
BIC = rep(0,ncol(beta.list))
for (k in 1:ncol(beta.list)){
BIC[k] = n*log(sum((x[,i] - x[,-i]%*%beta.list[,k])^2)) +
log(n)*sum(abs(beta.list[,k])>0)
}
ind = which.min(BIC)
lambda.opt[i] = lambdalist[ind]
}

return(lambda.opt)
}

############################################################
############################################################
############################################################
##### Nodewise Regression 'OR' Rule
############################################################
############################################################
############################################################

# Estimate the GGM using Nodewise regression + AND rule

neigh.or.lambda = function(x,lambda){

x = scale(x)

p = ncol(x)

n = nrow(x)

Omega = matrix(0,p,p)

for (i in 1:p){
Omega[i,-i] = as.matrix(glmnet(x[,-i], x[,i], family='gaussian', lambda =
lambda[i],
lambda.min.ratio = 0.05)$beta)
}

for (i in 1:p){
for (j in 1:p){
Omega[i,j] = max(abs(Omega[i,j]),abs(Omega[j,i]))
```

```
Omega[j,i] = Omega[i,j]
}}

# Compute the precision matrix

Edges_I = combn(1:p,2) # Inactive set of ordered pair (i,j)

# Candidate Set of Edges

for (t in 1:ncol(Edges_I)){
i = Edges_I[1,t]
j = Edges_I[2,t]
if(Omega[i,j]==0){Edges_I[,t]=c(0,0)}
}

# Compute betas

beta = matrix(0,p,p)

for (i in 1:p){

n_i = c(Edges_I[1,which(Edges_I[2,]==i)],
Edges_I[2,which(Edges_I[1,]==i)])

if (length(n_i)>0){beta[n_i,i] = coef(lm(x[,i] ~ 0 + x[,n_i]))}
}

vareps = x - x%*%beta

# Estimate Precision Matrix

Omega.hat = matrix(0,p,p)

diag(Omega.hat) = apply(vareps,2,var)^(-1)

for (e in which(Edges_I[2,]>0)){
  i = Edges_I[1,e]
  j = Edges_I[2,e]

  Omega.hat[i,j] = cov(vareps[,i],vareps[,j])*Omega.hat[i,i]*Omega.hat[j,j]
  Omega.hat[j,i] = Omega.hat[i,j]
}

# Make Omega Positive Definite

lambda_min = eigen(Omega.hat)$values[p]
if (lambda_min < 1e-6){Omega.hat = Omega.hat+(0.1+abs(lambda_min))*diag(p)}

######

return(list(beta,Omega.hat))}


###########################################################
###########################################################
###########################################################
##### Ridge Regression
###########################################################
###########################################################
###########################################################

# Ridge regression

ridge.lambda = function(x,lambda){
```

```
x = scale(x)

p = ncol(x)

n = nrow(x)

# Compute betas

beta = matrix(0,p,p)

for (i in 1:p){
beta[-i,i] = as.matrix(glmnet(x[,-i], x[,i], family='gaussian', alpha = 0,
lambda = lambda[i],
lambda.min.ratio = 0.05)$beta)
}

vareps = x - x%*%beta

# Set of edges

Edges_I = combn(1:p,2) # Inactive set of ordered pair (i,j)

# Estimate Precision Matrix

Omega.hat = matrix(0,p,p)

diag(Omega.hat) = apply(vareps,2,var)^(-1)

for (e in 1:ncol(Edges_I)){
  i = Edges_I[1,e]
  j = Edges_I[2,e]

  Omega.hat[i,j] = cov(vareps[,i],vareps[,j])*Omega.hat[i,i]*Omega.hat[j,j]
  Omega.hat[j,i] = Omega.hat[i,j]
}

# Make Omega Positive Definite

lambda_min = eigen(Omega.hat)$values[p]
if (lambda_min < 1e-6){Omega.hat = Omega.hat+(0.1+abs(lambda_min))*diag(p)}

######

return(list(beta,Omega.hat))}

############################################################
############################################################

# Function that performs k-fold cross validation on Ridge regression

cv_ridge = function(x, fold){

x = scale(x)

p = ncol(x)

lambda.opt = lapply(1:p, function(i) cv.glmnet(x[,-i], x[,i],
family='gaussian',alpha = 0,
nfolds=fold,lambda.min.ratio = 0.05)$lambda.min)

lambda.opt = unlist(lambda.opt)

######
```

```
return(lambda.opt)}

############################################################
############################################################
############################################################
##### K-fols CV for PCS
############################################################
############################################################
############################################################

# k-folds CV for PCS-SPACE

cv_PCS_space = function(x,lambda.opt,fold){

x = scale(x)

  cv.part = function(n, k) {
    ntest = floor(n/k)
    ntrain = n - ntest
    ind = sample(n)
    trainMat = matrix(NA, nrow=ntrain, ncol=k)
    testMat = matrix(NA, nrow=ntest, ncol=k)
    nn = 1:n
    for (j in 1:k) {
      sel = ((j-1)*ntest+1):(j*ntest)
      testMat[,j] = ind[sel ]
      sel2 =nn[ !(nn %in% sel) ]
      trainMat[,j] = ind[sel2]
    }
    return(list(trainMat=trainMat,testMat=testMat))
  }

 loss.cv = function(x.train,x.test,lambda.opt,taulist){
  R = Psi.space(x.train,lambda.opt)
  eps = lapply(1:length(taulist),function(i)
Psi_Screen_Beta(x.train,R,taulist[i])[[1]])
  loss.re = unlist(lapply(1:length(taulist), function(i) sum(colSums((x.test -
x.test%*%eps[[i]])^2))))
  return(loss.re)
 }

  n = nrow(x)
  p = ncol(x)
  part.list = cv.part(n, fold)

  taulist = seq(0.0001,1,length=100)

  loss.list = lapply(1:fold, function(k)
  loss.cv(x[part.list$trainMat[, k], ],x[part.list$testMat[,
k], ],lambda.opt,taulist))

  loss.re = matrix(unlist(loss.list), ncol = fold, byrow = FALSE)

  loss.mean = apply(loss.re, 1, mean)
  ind = which.min(loss.mean)
  tau_opt = taulist[ind]

  res = tau_opt
  return(res)
}

############################################################
############################################################
```

```
############################################################

# k-folds CV for PCS: Nodewise regression 'AND'

cv_PCS_nei_and = function(x,lambda.opt,fold){

x = scale(x)

  cv.part = function(n, k) {
    ntest = floor(n/k)
    ntrain = n - ntest
    ind = sample(n)
    trainMat = matrix(NA, nrow=ntrain, ncol=k)
    testMat = matrix(NA, nrow=ntest, ncol=k)
    nn = 1:n
    for (j in 1:k) {
      sel = ((j-1)*ntest+1):(j*ntest)
      testMat[,j] = ind[sel ]
      sel2 =nn[ !(nn %in% sel) ]
      trainMat[,j] = ind[sel2]
    }
    return(list(trainMat=trainMat,testMat=testMat))
  }

 loss.cv = function(x.train,x.test,lambda.opt,taulist){
  list.nei.alpha = neigh.and.lambda(x.train,lambda.opt)
  R = -cov2cor(list.nei.alpha[[2]])
  diag(R) = 1
  eps = lapply(1:length(taulist),function(i)
Psi_Screen_Beta(x.train,R,taulist[i])[[1]])
  loss.re = unlist(lapply(1:length(taulist), function(i) sum(colSums((x.test -
x.test%*%eps[[i]])^2))))
  return(loss.re)
 }

  n = nrow(x)
  p = ncol(x)
  part.list = cv.part(n, fold)

  taulist = seq(0.0001,1,length=100)

  loss.list = lapply(1:fold, function(k)
  loss.cv(x[part.list$trainMat[, k], ],x[part.list$testMat[,
k], ],lambda.opt,taulist))

  loss.re = matrix(unlist(loss.list), ncol = fold, byrow = FALSE)

  loss.mean = apply(loss.re, 1, mean)
  ind = which.min(loss.mean)
  tau_opt = taulist[ind]

  res = tau_opt
  return(res)
}

############################################################
############################################################
############################################################

# k-folds CV for PCS: Nodewise regression 'OR'

cv_PCS_nei_or = function(x,lambda.opt,fold){

x = scale(x)
```

```
  cv.part = function(n, k) {
    ntest = floor(n/k)
    ntrain = n - ntest
    ind = sample(n)
    trainMat = matrix(NA, nrow=ntrain, ncol=k)
    testMat = matrix(NA, nrow=ntest, ncol=k)
    nn = 1:n
    for (j in 1:k) {
      sel = ((j-1)*ntest+1):(j*ntest)
      testMat[,j] = ind[sel ]
      sel2 =nn[ !(nn %in% sel) ]
      trainMat[,j] = ind[sel2]
    }
    return(list(trainMat=trainMat,testMat=testMat))
  }

 loss.cv = function(x.train,x.test,lambda.opt,taulist){
  list.nei.alpha = neigh.or.lambda(x.train,lambda.opt)
  R = -cov2cor(list.nei.alpha[[2]])
  diag(R) = 1
  eps = lapply(1:length(taulist),function(i)
Psi_Screen_Beta(x.train,R,taulist[i])[[1]])
  loss.re = unlist(lapply(1:length(taulist), function(i) sum(colSums((x.test -
x.test%*%eps[[i]])^2))))
  return(loss.re)
 }

  n = nrow(x)
  p = ncol(x)
  part.list = cv.part(n, fold)

  taulist = seq(0.0001,1,length=100)

  loss.list = lapply(1:fold, function(k)
  loss.cv(x[part.list$trainMat[, k], ],x[part.list$testMat[,
k], ],lambda.opt,taulist))

  loss.re = matrix(unlist(loss.list), ncol = fold, byrow = FALSE)

  loss.mean = apply(loss.re, 1, mean)
  ind = which.min(loss.mean)
  tau_opt = taulist[ind]

  res = tau_opt
  return(res)
}


###########################################################
###########################################################
###########################################################

# k-folds CV for PCS: Glasso

cv_PCS_glasso = function(x,lambda.opt,fold){

  cv.part = function(n, k) {
    ntest = floor(n/k)
    ntrain = n - ntest
    ind = sample(n)
    trainMat = matrix(NA, nrow=ntrain, ncol=k)
    testMat = matrix(NA, nrow=ntest, ncol=k)
    nn = 1:n
```

```
    for (j in 1:k) {
      sel = ((j-1)*ntest+1):(j*ntest)
      testMat[,j] = ind[sel ]
      sel2 =nn[ !(nn %in% sel) ]
      trainMat[,j] = ind[sel2]
    }
    return(list(trainMat=trainMat,testMat=testMat))
  }

 loss.cv = function(x.train,x.test,lambda.opt,taulist){
  Omegalist = as.matrix(huge(x.train,lambda = lambda.opt ,scr = F, method =
"glasso")$icov[[1]])
  R = -cov2cor(Omegalist)
  diag(R) = 1
  eps = lapply(1:length(taulist),function(i)
Psi_Screen_Beta(x.train,R,taulist[i])[[1]])
  loss.re = unlist(lapply(1:length(taulist), function(i) sum(colSums((x.test -
x.test%*%eps[[i]])^2))))
  return(loss.re)
 }

  n = nrow(x)
  p = ncol(x)
  part.list = cv.part(n, fold)

  taulist = seq(0.0001,1,length=100)

  loss.list = lapply(1:fold, function(k)
  loss.cv(x[part.list$trainMat[, k], ],x[part.list$testMat[,
k], ],lambda.opt,taulist))

  loss.re = matrix(unlist(loss.list), ncol = fold, byrow = FALSE)

  loss.mean = apply(loss.re, 1, mean)
  ind = which.min(loss.mean)
  tau_opt = taulist[ind]

  res = tau_opt
  return(res)
}

############################################################
############################################################
############################################################

# k-folds CV for PCS: Ridge

cv_PCS_ridge = function(x,lambda.opt,fold){

x = scale(x)

  cv.part = function(n, k) {
    ntest = floor(n/k)
    ntrain = n - ntest
    ind = sample(n)
    trainMat = matrix(NA, nrow=ntrain, ncol=k)
    testMat = matrix(NA, nrow=ntest, ncol=k)
    nn = 1:n
    for (j in 1:k) {
      sel = ((j-1)*ntest+1):(j*ntest)
      testMat[,j] = ind[sel ]
      sel2 =nn[ !(nn %in% sel) ]
      trainMat[,j] = ind[sel2]
    }
```

```
    return(list(trainMat=trainMat,testMat=testMat))
  }

 loss.cv = function(x.train,x.test,lambda.opt,taulist){
  list.ridge = ridge.lambda(x.train,lambda.opt)
  R = -cov2cor(list.ridge[[2]])
  diag(R) = 1
  eps = lapply(1:length(taulist),function(i)
Psi_Screen_Beta(x.train,R,taulist[i])[[1]])
  loss.re = unlist(lapply(1:length(taulist), function(i) sum(colSums((x.test -
x.test%*%eps[[i]])^2))))
  return(loss.re)
 }

  n = nrow(x)
  p = ncol(x)
  part.list = cv.part(n, fold)

  taulist = seq(0.0001,1,length=100)

  loss.list = lapply(1:fold, function(k)
  loss.cv(x[part.list$trainMat[, k], ],x[part.list$testMat[,
k], ],lambda.opt,taulist))

  loss.re = matrix(unlist(loss.list), ncol = fold, byrow = FALSE)

  loss.mean = apply(loss.re, 1, mean)
  ind = which.min(loss.mean)
  tau_opt = taulist[ind]

  res = tau_opt
  return(res)
}

###########################################################
###########################################################
###########################################################

##############################################################
##############################################################
##############################################################
##############################################################
##############################################################
##############################################################
##############################################################
##############################################################
##############################################################
##############################################################
##############################################################
##############################################################
##############################################################
##############################################################
##############################################################
##############################################################
##############################################################
##############################################################
##############################################################
##############################################################


###########################################################
###########################################################
###########################################################
##### GGM Estimation
```

```
############################################################
############################################################
############################################################

# Glasso 10-folds CV1

  load(file = "sample_1_p_200_n_100.RData")
  rho.list = cv_glasso(x,fold=10)
  rho = unlist(rho.list$rho_opt)
  list.glasso.cv = as.matrix(huge(x,lambda = rho,scr = F,method = "glasso")
$icov[[1]])
  lambda.opt = rho
  save(lambda.opt, file = "lambda_glasso_cv_1_p_200_n_100.RData")
  save(list.glasso.cv, file = "glasso_cv_1_p_200_n_100.RData")

# Glasso 10-folds CV1-1se

  rho.ls1 = unlist(rho.list$rho_ls1)
  list.glasso.cv.ls1 = as.matrix(huge(x,lambda = rho.ls1,scr = F,method =
"glasso")$icov[[1]])
  lambda.opt = rho.ls1
  save(lambda.opt, file = "lambda_glasso_cv_ls1_1_p_200_n_100.RData")
  save(list.glasso.cv.ls1, file = "glasso_cv_ls1_1_p_200_n_100.RData")

# Glasso 10-folds CV2

  rho = unlist(rho.list$rho2_opt)
  list.glasso.cv2 = as.matrix(huge(x,lambda = rho,scr = F,method = "glasso")
$icov[[1]])
  lambda.opt = rho
  save(lambda.opt, file = "lambda_glasso_cv2_1_p_200_n_100.RData")
  save(list.glasso.cv2, file = "glasso_cv2_1_p_200_n_100.RData")

# Glasso 10-folds CV2-1se

  rho.ls1 = unlist(rho.list$rho2_ls1)
  list.glasso.cv2.ls1 = as.matrix(huge(x,lambda = rho.ls1,scr = F,method =
"glasso")$icov[[1]])
  lambda.opt = rho.ls1
  save(lambda.opt, file = "lambda_glasso_cv2_ls1_1_p_200_n_100.RData")
  save(list.glasso.cv2.ls1, file = "glasso_cv2_ls1_1_p_200_n_100.RData")

#######################################################
#######################################################
#######################################################
#######################################################

# Glasso EBIC

  load(file = "sample_1_p_200_n_100.RData")
  fit.ebic =
huge.select(huge(x,lambda=seq(0.001,max(abs(cov(x))),length=100),method =
"glasso"),
  ebic.gamma = 0.5,criterion = "ebic")
  lambda.opt = fit.ebic$opt.lambda
  list.glasso.ebic = as.matrix(fit.ebic$opt.icov)
  save(lambda.opt, file = "lambda_glasso_ebic_1_p_200_n_100.RData")
  save(list.glasso.ebic, file = "glasso_ebic_1_p_200_n_100.RData")

#######################################################
#######################################################
#######################################################
#######################################################
```

```
# Glasso BIC

  load(file = "sample_1_p_200_n_100.RData")
  fit.bic =
huge.select(huge(x,lambda=seq(0.001,max(abs(cov(x))),length=100),method =
"glasso"),
  ebic.gamma = 0,criterion = "ebic")
  lambda.opt = fit.bic$opt.lambda
  list.glasso.bic = as.matrix(fit.bic$opt.icov)
  save(lambda.opt, file = "lambda_glasso_bic_1_p_200_n_100.RData")
  save(list.glasso.bic, file = "glasso_bic_1_p_200_n_100.RData")

######################################################
######################################################
######################################################
######################################################

# SPACE with 10-folds CV

  load(file = "sample_1_p_200_n_100.RData")
  lambda.list = cv_space(x,fold=10)
  lambda.opt = lambda.list$alpha_opt
  R.opt = Psi.space(x,lambda.opt)
  list.space.cv = PCS_GGM(x,R.opt,0)
  save(lambda.opt, file = "lambda_space_cv_1_p_200_n_100.RData")
  save(list.space.cv, file = "space_cv_1_p_200_n_100.RData")

  lambda.ls1 = lambda.list$alpha_ls1
  R.opt = Psi.space(x,lambda.ls1)
  list.space.cv.ls1 = PCS_GGM(x,R.opt,0)
  lambda.opt = lambda.ls1
  save(lambda.opt, file = "lambda_space_cv_ls1_1_p_200_n_100.RData")
  save(list.space.cv.ls1, file = "space_cv_ls1_1_p_200_n_100.RData")

######################################################
######################################################
######################################################
######################################################

# SPACE with finite sample results result

  load(file = "sample_1_p_200_n_100.RData")
  lambda.opt = sqrt(nrow(x))*qnorm(1-0.05/(2*ncol(x)^2))
  R.opt = Psi.space(x,lambda.opt)
  list.space.alpha = PCS_GGM(x,R.opt,0)
  save(lambda.opt, file = "lambda_space_alpha_1_p_200_n_100.RData")
  save(list.space.alpha, file = "space_alpha_1_p_200_n_100.RData")

######################################################
######################################################
######################################################
######################################################

# SPACE with BIC

  load(file = "sample_1_p_200_n_100.RData")
  lambda.opt = BIC_space(x)
  R.opt = Psi.space(x,lambda.opt)
  list.space.bic = PCS_GGM(x,R.opt,0)
  save(lambda.opt, file = "lambda_space_bic_1_p_200_n_100.RData")
  save(list.space.bic, file = "space_bic_1_p_200_n_100.RData")

######################################################
######################################################
```

```
######################################################
######################################################

# Nodewise regression 'AND' with 10-folds CV

  load(file = "sample_1_p_200_n_100.RData")
  lambda.fit = cv_Neigh(x,10)
  lambda.opt = lambda.fit[[1]]
  list.nei.cv = neigh.and.lambda(x,lambda.opt)
  save(lambda.opt, file = "lambda_nei_and_cv_1_p_200_n_100.RData")
  save(list.nei.cv, file = "nei_and_cv_1_p_200_n_100.RData")

# Nodewise regression 'AND' with 10-folds CV-ls1

  list.nei.cv = neigh.or.lambda(x,lambda.opt)
  save(lambda.opt, file = "lambda_nei_or_cv_1_p_200_n_100.RData")
  save(list.nei.cv, file = "nei_or_cv_1_p_200_n_100.RData")

# Nodewise regression 'OR' with 10-folds CV

  lambda.ls1 = lambda.fit[[2]]
  list.nei.cv.ls1 = neigh.and.lambda(x,lambda.ls1)
  lambda.opt = lambda.ls1
  save(lambda.opt, file = "lambda_nei_and_cv_ls1_1_p_200_n_100.RData")
  save(list.nei.cv.ls1, file = "nei_and_cv_ls1_1_p_200_n_100.RData")

# Nodewise regression 'OR' with 10-folds CV-ls1

  list.nei.cv.ls1 = neigh.or.lambda(x,lambda.ls1)
  save(lambda.opt, file = "lambda_nei_or_cv_ls1_1_p_200_n_100.RData")
  save(list.nei.cv.ls1, file = "nei_or_cv_ls1_1_p_200_n_100.RData")

######################################################
######################################################
######################################################
######################################################

# Nodewise regression 'AND' with finite sample result

  load(file = "sample_1_p_200_n_100.RData")
  lambda.opt = rep((1/(sqrt(nrow(x))))*qnorm(1-0.05/(2*ncol(x)^2)),ncol(x))
  list.nei.alpha = neigh.and.lambda(x,lambda.opt)
  save(lambda.opt, file = "lambda_nei_and_alpha_1_p_200_n_100.RData")
  save(list.nei.alpha, file = "nei_and_alpha_1_p_200_n_100.RData")

# Nodewise regression 'OR' with finite sample result

  list.nei.alpha = neigh.or.lambda(x,lambda.opt)
  save(lambda.opt, file = "lambda_nei_or_alpha_1_p_200_n_100.RData")
  save(list.nei.alpha, file = "nei_or_alpha_1_p_200_n_100.RData")

######################################################
######################################################
######################################################
######################################################

# Nodewise regression 'AND' with BIC

  load(file = "sample_1_p_200_n_100.RData")
  lambda.opt = BIC_Neigh(x)
  list.nei.bic = neigh.and.lambda(x,lambda.opt)
  save(lambda.opt, file = "lambda_nei_and_bic_1_p_200_n_100.RData")
  save(list.nei.bic, file = "nei_and_bic_1_p_200_n_100.RData")
```

```
# Nodewise regression 'OR' with BIC

  list.nei.bic = neigh.or.lambda(x,lambda.opt)
  save(lambda.opt, file = "lambda_nei_or_bic_1_p_200_n_100.RData")
  save(list.nei.bic, file = "nei_or_bic_1_p_200_n_100.RData")

######################################################
######################################################
######################################################
######################################################

# Ridge regression with 10-folds CV

  load(file = "sample_1_p_200_n_100.RData")
  lambda.opt = cv_ridge(x,10)
  list.ridge.cv = ridge.lambda(x,lambda.opt)
  save(lambda.opt, file = "lambda_ridge_cv_1_p_200_n_100.RData")
  save(list.ridge.cv, file = "ridge_cv_1_p_200_n_100.RData")

######################################################
######################################################
######################################################
######################################################
######################################################
######################################################
######################################################
######################################################

###########################################################
###########################################################
###########################################################
##### GGM Estimation with PCS
###########################################################
###########################################################
###########################################################

# Glasso 10-folds CV1

  load(file = "sample_1_p_200_n_100.RData")
  load(file = "glasso_cv_1_p_200_n_100.RData")
  load(file = "lambda_glasso_cv_1_p_200_n_100.RData")
  R.opt = -cov2cor(list.glasso.cv)
  diag(R.opt) = 1
  tau.opt = cv_PCS_glasso(x,lambda.opt,fold=10)
  list.glasso.cv.pcs = PCS_GGM(x,R.opt,tau.opt)
  save(tau.opt, file = "tau_glasso_cv_1_p_200_n_100.RData")
  save(list.glasso.cv.pcs, file = "PCS_glasso_cv_1_p_200_n_100.RData")

# Glasso 10-folds CV1-1se

  load(file = "sample_1_p_200_n_100.RData")
  load(file = "glasso_cv_ls1_1_p_200_n_100.RData")
  load(file = "lambda_glasso_cv_ls1_1_p_200_n_100.RData")
  R.opt = -cov2cor(list.glasso.cv.ls1)
  diag(R.opt) = 1
  tau.opt = cv_PCS_glasso(x,lambda.opt,fold=10)
  list.glasso.cv.ls1.pcs = PCS_GGM(x,R.opt,tau.opt)
  save(tau.opt, file = "tau_glasso_cv_ls1_1_p_200_n_100.RData")
  save(list.glasso.cv.ls1.pcs, file = "PCS_glasso_cv_ls1_1_p_200_n_100.RData")

# Glasso 10-folds CV2

  load(file = "sample_1_p_200_n_100.RData")
  load(file = "glasso_cv2_1_p_200_n_100.RData")
```

```
  load(file = "lambda_glasso_cv2_1_p_200_n_100.RData")
  R.opt = -cov2cor(list.glasso.cv2)
  diag(R.opt) = 1
  tau.opt = cv_PCS_glasso(x,lambda.opt,fold=10)
  list.glasso.cv2.pcs = PCS_GGM(x,R.opt,tau.opt)
  save(tau.opt, file = "tau_glasso_cv2_1_p_200_n_100.RData")
  save(list.glasso.cv2.pcs, file = "PCS_glasso_cv2_1_p_200_n_100.RData")

# Glasso 10-folds CV2-1se

  load(file = "sample_1_p_200_n_100.RData")
  load(file = "glasso_cv2_ls1_1_p_200_n_100.RData")
  load(file = "lambda_glasso_cv2_ls1_1_p_200_n_100.RData")
  R.opt = -cov2cor(list.glasso.cv2.ls1)
  diag(R.opt) = 1
  tau.opt = cv_PCS_glasso(x,lambda.opt,fold=10)
  list.glasso.cv2.ls1.pcs = PCS_GGM(x,R.opt,tau.opt)
  save(tau.opt, file = "tau_glasso_cv2_ls1_1_p_200_n_100.RData")
  save(list.glasso.cv2.ls1.pcs, file = "PCS_glasso_cv2_ls1_1_p_200_n_100.RData")


######################################################
######################################################
######################################################
######################################################

# Glasso EBIC

  load(file = "sample_1_p_200_n_100.RData")
  load(file = "glasso_ebic_1_p_200_n_100.RData")
  load(file = "lambda_glasso_ebic_1_p_200_n_100.RData")
  R.opt = -cov2cor(list.glasso.ebic)
  diag(R.opt) = 1
  tau.opt = cv_PCS_glasso(x,lambda.opt,fold=10)
  list.glasso.ebic.pcs = PCS_GGM(x,R.opt,tau.opt)
  save(tau.opt, file = "tau_glasso_ebic_1_p_200_n_100.RData")
  save(list.glasso.ebic.pcs, file = "PCS_glasso_ebic_1_p_200_n_100.RData")


######################################################
######################################################
######################################################
######################################################

# Glasso BIC

  load(file = "sample_1_p_200_n_100.RData")
  load(file = "glasso_bic_1_p_200_n_100.RData")
  load(file = "lambda_glasso_bic_1_p_200_n_100.RData")
  R.opt = -cov2cor(list.glasso.bic)
  diag(R.opt) = 1
  tau.opt = cv_PCS_glasso(x,lambda.opt,fold=10)
  list.glasso.bic.pcs = PCS_GGM(x,R.opt,tau.opt)
  save(tau.opt, file = "tau_glasso_bic_1_p_200_n_100.RData")
  save(list.glasso.bic.pcs, file = "PCS_glasso_bic_1_p_200_n_100.RData")

######################################################
######################################################
######################################################
######################################################

# SPACE with 10-folds CV

  load(file = "sample_1_p_200_n_100.RData")
```

```
  load(file = "space_cv_1_p_200_n_100.RData")
  load(file = "lambda_space_cv_1_p_200_n_100.RData")
  R.opt = Psi.space(x,lambda.opt)
  #R.opt = -cov2cor(list.space.cv[[2]])
  #diag(R.opt) = 1
  tau.opt = cv_PCS_space(x,lambda.opt,10)
  list.space.cv.pcs = PCS_GGM(x,R.opt,tau.opt)
  save(tau.opt, file = "tau_space_cv_1_p_200_n_100.RData")
  save(list.space.cv.pcs, file = "PCS_space_cv_1_p_200_n_100.RData")

  load(file = "sample_1_p_200_n_100.RData")
  load(file = "space_cv_ls1_1_p_200_n_100.RData")
  load(file = "lambda_space_cv_ls1_1_p_200_n_100.RData")
  R.opt = Psi.space(x,lambda.opt)
  #R.opt = -cov2cor(list.space.cv.ls1[[2]])
  #diag(R.opt) = 1
  tau.opt = cv_PCS_space(x,lambda.opt,10)
  list.space.cv.ls1.pcs = PCS_GGM(x,R.opt,tau.opt)
  save(tau.opt, file = "tau_space_cv_ls1_1_p_200_n_100.RData")
  save(list.space.cv.ls1.pcs, file = "PCS_space_cv_ls1_1_p_200_n_100.RData")

######################################################
######################################################
######################################################
######################################################

# SPACE with finite sample results result

  load(file = "sample_1_p_200_n_100.RData")
  load(file = "space_alpha_1_p_200_n_100.RData")
  load(file = "lambda_space_alpha_1_p_200_n_100.RData")
  R.opt = Psi.space(x,lambda.opt)
  #R.opt = -cov2cor(list.space.alpha[[2]])
  #diag(R.opt) = 1
  tau.opt = cv_PCS_space(x,lambda.opt,10)
  list.space.alpha.pcs = PCS_GGM(x,R.opt,tau.opt)
  save(tau.opt, file = "tau_space_alpha_1_p_200_n_100.RData")
  save(list.space.alpha.pcs, file = "PCS_space_alpha_1_p_200_n_100.RData")

######################################################
######################################################
######################################################
######################################################

# SPACE with BIC

  load(file = "sample_1_p_200_n_100.RData")
  load(file = "space_bic_1_p_200_n_100.RData")
  load(file = "lambda_space_bic_1_p_200_n_100.RData")
  R.opt = Psi.space(x,lambda.opt)
  #R.opt = -cov2cor(list.space.bic[[2]])
  #diag(R.opt) = 1
  tau.opt = cv_PCS_space(x,lambda.opt,10)
  list.space.bic.pcs = PCS_GGM(x,R.opt,tau.opt)
  save(tau.opt, file = "tau_space_bic_1_p_200_n_100.RData")
  save(list.space.bic.pcs, file = "PCS_space_bic_1_p_200_n_100.RData")

######################################################
######################################################
######################################################
######################################################

# Nodewise regression 'AND' with 10-folds CV
```

```
  load(file = "sample_1_p_200_n_100.RData")
  load(file = "nei_and_cv_1_p_200_n_100.RData")
  load(file = "lambda_nei_and_cv_1_p_200_n_100.RData")
  R.opt = -cov2cor(list.nei.cv[[2]])
  diag(R.opt) = 1
  tau.opt = cv_PCS_nei_and(x,lambda.opt,10)
  list.nei.cv.pcs = PCS_GGM(x,R.opt,tau.opt)
  save(tau.opt, file = "tau_nei_and_cv_1_p_200_n_100.RData")
  save(list.nei.cv.pcs, file = "PCS_nei_and_cv_1_p_200_n_100.RData")

# Nodewise regression 'AND' with 10-folds CV-ls1

  load(file = "sample_1_p_200_n_100.RData")
  load(file = "nei_or_cv_1_p_200_n_100.RData")
  load(file = "lambda_nei_or_cv_1_p_200_n_100.RData")
  R.opt = -cov2cor(list.nei.cv[[2]])
  diag(R.opt) = 1
  tau.opt = cv_PCS_nei_or(x,lambda.opt,10)
  list.nei.cv.pcs = PCS_GGM(x,R.opt,tau.opt)
  save(tau.opt, file = "tau_nei_or_cv_1_p_200_n_100.RData")
  save(list.nei.cv.pcs, file = "PCS_nei_or_cv_1_p_200_n_100.RData")

# Nodewise regression 'OR' with 10-folds CV

  load(file = "sample_1_p_200_n_100.RData")
  load(file = "nei_and_cv_ls1_1_p_200_n_100.RData")
  load(file = "lambda_nei_and_cv_ls1_1_p_200_n_100.RData")
  R.opt = -cov2cor(list.nei.cv.ls1[[2]])
  diag(R.opt) = 1
  tau.opt = cv_PCS_nei_and(x,lambda.opt,10)
  list.nei.cv.ls1.pcs = PCS_GGM(x,R.opt,tau.opt)
  save(tau.opt, file = "tau_nei_and_cv_ls1_1_p_200_n_100.RData")
  save(list.nei.cv.ls1.pcs, file = "PCS_nei_and_cv_ls1_1_p_200_n_100.RData")

# Nodewise regression 'OR' with 10-folds CV-ls1

  load(file = "sample_1_p_200_n_100.RData")
  load(file = "nei_or_cv_ls1_1_p_200_n_100.RData")
  load(file = "lambda_nei_or_cv_ls1_1_p_200_n_100.RData")
  R.opt = -cov2cor(list.nei.cv.ls1[[2]])
  diag(R.opt) = 1
  tau.opt = cv_PCS_nei_or(x,lambda.opt,10)
  list.nei.cv.ls1.pcs = PCS_GGM(x,R.opt,tau.opt)
  save(tau.opt, file = "tau_nei_or_cv_ls1_1_p_200_n_100.RData")
  save(list.nei.cv.ls1.pcs, file = "PCS_nei_or_cv_ls1_1_p_200_n_100.RData")

######################################################
######################################################
######################################################
######################################################

# Nodewise regression 'AND' with finite sample result

  load(file = "sample_1_p_200_n_100.RData")
  load(file = "nei_and_alpha_1_p_200_n_100.RData")
  load(file = "lambda_nei_and_alpha_1_p_200_n_100.RData")
  R.opt = -cov2cor(list.nei.alpha[[2]])
  diag(R.opt) = 1
  tau.opt = cv_PCS_nei_and(x,lambda.opt,10)
  list.nei.alpha.pcs = PCS_GGM(x,R.opt,tau.opt)
  save(tau.opt, file = "tau_nei_and_alpha_1_p_200_n_100.RData")
  save(list.nei.alpha.pcs, file = "PCS_nei_and_alpha_1_p_200_n_100.RData")

# Nodewise regression 'OR' with finite sample result
```

```
  load(file = "sample_1_p_200_n_100.RData")
  load(file = "nei_or_alpha_1_p_200_n_100.RData")
  load(file = "lambda_nei_or_alpha_1_p_200_n_100.RData")
  R.opt = -cov2cor(list.nei.alpha[[2]])
  diag(R.opt) = 1
  tau.opt = cv_PCS_nei_or(x,lambda.opt,10)
  list.nei.alpha.pcs = PCS_GGM(x,R.opt,tau.opt)
  save(tau.opt, file = "tau_nei_or_alpha_1_p_200_n_100.RData")
  save(list.nei.alpha.pcs, file = "PCS_nei_or_alpha_1_p_200_n_100.RData")

######################################################
######################################################
######################################################
######################################################

# Nodewise regression 'AND' with BIC

  load(file = "sample_1_p_200_n_100.RData")
  load(file = "nei_and_bic_1_p_200_n_100.RData")
  load(file = "lambda_nei_and_bic_1_p_200_n_100.RData")
  R.opt = -cov2cor(list.nei.bic[[2]])
  diag(R.opt) = 1
  tau.opt = cv_PCS_nei_and(x,lambda.opt,10)
  list.nei.bic.pcs = PCS_GGM(x,R.opt,tau.opt)
  save(tau.opt, file = "tau_nei_and_bic_1_p_200_n_100.RData")
  save(list.nei.bic.pcs, file = "PCS_nei_and_bic_1_p_200_n_100.RData")

# Nodewise regression 'OR' with BIC

  load(file = "sample_1_p_200_n_100.RData")
  load(file = "nei_or_bic_1_p_200_n_100.RData")
  load(file = "lambda_nei_or_bic_1_p_200_n_100.RData")
  R.opt = -cov2cor(list.nei.bic[[2]])
  diag(R.opt) = 1
  tau.opt = cv_PCS_nei_or(x,lambda.opt,10)
  list.nei.bic.pcs = PCS_GGM(x,R.opt,tau.opt)
  save(tau.opt, file = "tau_nei_or_bic_1_p_200_n_100.RData")
  save(list.nei.bic.pcs, file = "PCS_nei_or_bic_1_p_200_n_100.RData")

######################################################
######################################################
######################################################
######################################################

# Ridge regression with 10-folds CV

  load(file = "sample_1_p_200_n_100.RData")
  load(file = "ridge_cv_1_p_200_n_100.RData")
  load(file = "lambda_ridge_cv_1_p_200_n_100.RData")
  R.opt = -cov2cor(list.ridge.cv[[2]])
  diag(R.opt) = 1
  tau.opt = cv_PCS_ridge(x,lambda.opt,10)
  list.ridge.cv.pcs = PCS_GGM(x,R.opt,tau.opt)
  save(tau.opt, file = "tau_ridge_cv_1_p_200_n_100.RData")
  save(list.ridge.cv.pcs, file = "PCS_ridge_cv_1_p_200_n_100.RData")

######################################################
######################################################
######################################################
######################################################
######################################################
######################################################
######################################################
```

```
######################################################

############################################################
############################################################
############################################################
############################################################
############################################################
############################################################
##########         Performance Measures      ###############
############################################################
############################################################
############################################################
############################################################
############################################################
############################################################

ls()

rm(list=ls())

library(psych)

library(corrplot)

library(qgraph)

############################################################
############################################################
######          Classification Performance           #######
############################################################
############################################################

## Performance is a function that computes
# sensitivity and specificity of one matrix

performance = function(x,omega){

p = ncol(omega)

A.true = diag(p)

A.x = diag(p)

for (i in 1:p){A.true[i,] = ifelse(abs(omega[i,])<=0,0,1)}

for (i in 1:p){A.x[i,] = ifelse(abs(x[i,])<=0,0,1)}

Edges_I = combn(1:p,2) # Inactive set of ordered pair (i,j)
Edges_x = combn(1:p,2) # Inactive set of ordered pair (i,j)

# Set of Edges

for (t in 1:ncol(Edges_I)){
i = Edges_I[1,t]
j = Edges_I[2,t]
if(A.true[i,j]==0){Edges_I[,t]=c(0,0)}
}

for (t in 1:ncol(Edges_x)){
i = Edges_x[1,t]
j = Edges_x[2,t]
if(A.x[i,j]==0){Edges_x[,t]=c(0,0)}
}
```

```
P = which(Edges_I[1,]>0)
N = which(Edges_I[1,]==0)

P.x = which(Edges_x[1,]>0)
N.x = which(Edges_x[1,]==0)

TP = length(which(P.x %in% P))
TN = length(which(N.x %in% N))

FP = length(P.x) - TP

FN = length(N.x) - TN

TPR = TP/length(P)
FPR = 1 - TN/length(N)

MCC = ((TP*TN)-(FP*FN))/((TP+FP)^0.5*(TP+FN)^0.5*(TN+FP)^0.5*(TN+FN)^0.5)

if (is.nan(MCC)==TRUE){MCC=0}

return(list(TPR=TPR,FPR=FPR,TP=TP,FP=FP))
}

##############################################################
##############################################################
##############################################################

############################################################
##### Compute Adjacency Matrix
############################################################

Adj_mat = function(Omega){

p = dim(Omega)[1]

Adj_mat = matrix(0,p,p)

 for (i in 1:p){
    for (j in 1:p){
      if (abs(Omega[i,j])<=0){Adj_mat[i,j]=0}
      else (Adj_mat[i,j]=1)
    }
 }
return(Adj_mat)}


############################################################
##### Compute Partial Correlation Matrix
############################################################

# Function that estimates the Regression Weights and Omega

Omega.GGM = function(x,Adj.mat){

x = scale(x)

n = dim(x)[1]

p = dim(x)[2]

Edges_I = combn(1:p,2) # Inactive set of ordered pair (i,j)

# Set of Edges
```

```
for (t in 1:ncol(Edges_I)){
i = Edges_I[1,t]
j = Edges_I[2,t]
if(Adj.mat[i,j]==0){Edges_I[,t]=c(0,0)}
}

####

# Compute Prediction Errors and betas

beta = matrix(0,p,p)

for (i in 1:p){

n_i = c(Edges_I[1,which(Edges_I[2,]==i)],
Edges_I[2,which(Edges_I[1,]==i)])

if (length(n_i)>0){beta[n_i,i] = coef(lm(x[,i] ~ 0 + x[,n_i]))}
}

vareps = x - x%*%beta

# Compute the precision matrix

Omega = matrix(0,p,p)

diag(Omega) = apply(vareps,2,var)^(-1)

for (e in which(Edges_I[2,]>0)){
  i = Edges_I[1,e]
  j = Edges_I[2,e]

  Omega[i,j] = cov(vareps[,i],vareps[,j])*Omega[i,i]*Omega[j,j]
  Omega[j,i] = Omega[i,j]
}

# Make Omega Positive Definite

lambda_min = eigen(Omega)$values[p]
if (lambda_min < 1e-6){Omega = Omega+(0.1+abs(lambda_min))*diag(p)}

######

R = -cov2cor(Omega)
diag(R) = 1

# Make R Positive Definite

lambda_min = eigen(R)$values[p]
if (lambda_min < 1e-6){R = R+(0.1+abs(lambda_min))*diag(p)}

R = cov2cor(R)

return(R)}

##############################################################
##############################################################
##############################################################
##############################################################
##############################################################
##############################################################
##############################################################
##############################################################
##############################################################
```

```
##############################################################

############################################################
##### True Positives and False Positives
############################################################

# Load data

load(file="sample_1_p_20_n_100.RData")

# Load population partial correlation matrix

load(file="Pcor_1_p_20.RData")

######################################################
######################################################

# Glasso-CV1

load(file = "glasso_cv_1_p_20_n_100.RData")
performance(list.glasso.cv,R)

# PCS-Glasso-CV1

load(file = "pcs_glasso_cv_1_p_20_n_100.RData")
performance(list.glasso.cv.pcs[[2]],R)

######################################################
######################################################

# Glasso-CV1-1se

load(file = "glasso_cv_ls1_1_p_20_n_100.RData")
performance(list.glasso.cv.ls1,R)

# PCS-Glasso-CV1-1se

load(file = "pcs_glasso_cv_ls1_1_p_20_n_100.RData")
performance(list.glasso.cv.ls1.pcs[[2]],R)

######################################################
######################################################

# Glasso-CV2

load(file = "glasso_cv2_1_p_20_n_100.RData")
performance(list.glasso.cv2,R)

# PCS-Glasso-CV2

load(file = "pcs_glasso_cv2_1_p_20_n_100.RData")
performance(list.glasso.cv2.pcs[[2]],R)

######################################################
######################################################

# Glasso-CV2-1se

load(file = "glasso_cv2_ls1_1_p_20_n_100.RData")
performance(list.glasso.cv2.ls1,R)

# PCS-Glasso-CV2-1se

load(file = "pcs_glasso_cv2_ls1_1_p_20_n_100.RData")
```

```
performance(list.glasso.cv2.ls1.pcs[[2]],R)

######################################################
######################################################

# Glasso-EBIC

load(file = "glasso_ebic_1_p_20_n_100.RData")
performance(list.glasso.ebic,R)

# PCS-Glasso-EBIC

load(file = "pcs_glasso_ebic_1_p_20_n_100.RData")
performance(list.glasso.ebic.pcs[[2]],R)

######################################################
######################################################

# Glasso-BIC

load(file = "glasso_bic_1_p_20_n_100.RData")
performance(list.glasso.bic,R)

# PCS-Glasso-BIC

load(file = "pcs_glasso_bic_1_p_20_n_100.RData")
performance(list.glasso.bic.pcs[[2]],R)

######################################################
######################################################

# SPACE-CV

load(file = "space_cv_1_p_20_n_100.RData")
performance(list.space.cv[[2]],R)

# PCS-SPACE-CV

load(file = "pcs_space_cv_1_p_20_n_100.RData")
performance(list.space.cv.pcs[[2]],R)

######################################################
######################################################

# SPACE-CV-1se

load(file = "space_cv_ls1_1_p_20_n_100.RData")
performance(list.space.cv.ls1[[2]],R)

# PCS-SPACE-CV-1se

load(file = "pcs_space_cv_ls1_1_p_20_n_100.RData")
performance(list.space.cv.ls1.pcs[[2]],R)

######################################################
######################################################

# SPACE-FSR

load(file = "space_alpha_1_p_20_n_100.RData")
performance(list.space.alpha[[2]],R)

# PCS-SPACE-FSR
```

```
load(file = "pcs_space_alpha_1_p_20_n_100.RData")
performance(list.space.alpha.pcs[[2]],R)

######################################################
######################################################

# SPACE-BIC

load(file = "space_bic_1_p_20_n_100.RData")
performance(list.space.bic[[2]],R)

# PCS-SPACE-BIC

load(file = "pcs_space_bic_1_p_20_n_100.RData")
performance(list.space.bic.pcs[[2]],R)

######################################################
######################################################

# NR-AND-CV

load(file = "nei_and_cv_1_p_20_n_100.RData")
performance(list.nei.cv[[2]],R)

# PCS-NR-AND-CV

load(file = "pcs_nei_and_cv_1_p_20_n_100.RData")
performance(list.nei.cv.pcs[[2]],R)

######################################################
######################################################

# NR-AND-CV-1se

load(file = "nei_and_cv_ls1_1_p_20_n_100.RData")
performance(list.nei.cv.ls1[[2]],R)

# PCS-NR-AND-CV-1se

load(file = "pcs_nei_and_cv_ls1_1_p_20_n_100.RData")
performance(list.nei.cv.ls1.pcs[[2]],R)

######################################################
######################################################

# NR-AND-FSR

load(file = "nei_and_alpha_1_p_20_n_100.RData")
performance(list.nei.alpha[[2]],R)

# PCS-NR-AND-FSR

load(file = "pcs_nei_and_alpha_1_p_20_n_100.RData")
performance(list.nei.alpha.pcs[[2]],R)

######################################################
######################################################

# NR-AND-BIC

load(file = "nei_and_bic_1_p_20_n_100.RData")
performance(list.nei.bic[[2]],R)

# PCS-NR-AND-BIC
```

```
load(file = "pcs_nei_and_bic_1_p_20_n_100.RData")
performance(list.nei.bic.pcs[[2]],R)

######################################################
######################################################

# NR-OR-CV

load(file = "nei_or_cv_1_p_20_n_100.RData")
performance(list.nei.cv[[2]],R)

# PCS-NR-OR-CV

load(file = "pcs_nei_or_cv_1_p_20_n_100.RData")
performance(list.nei.cv.pcs[[2]],R)

######################################################
######################################################

# NR-OR-CV-1se

load(file = "nei_or_cv_ls1_1_p_20_n_100.RData")
performance(list.nei.cv.ls1[[2]],R)

# PCS-NR-OR-CV-1se

load(file = "pcs_nei_or_cv_ls1_1_p_20_n_100.RData")
performance(list.nei.cv.ls1.pcs[[2]],R)

######################################################
######################################################

# NR-OR-FSR

load(file = "nei_or_alpha_1_p_20_n_100.RData")
performance(list.nei.alpha[[2]],R)

# PCS-NR-OR-FSR

load(file = "pcs_nei_or_alpha_1_p_20_n_100.RData")
performance(list.nei.alpha.pcs[[2]],R)

######################################################
######################################################

# NR-OR-BIC

load(file = "nei_or_bic_1_p_20_n_100.RData")
performance(list.nei.bic[[2]],R)

# PCS-NR-OR-BIC

load(file = "pcs_nei_or_bic_1_p_20_n_100.RData")
performance(list.nei.bic.pcs[[2]],R)

######################################################
######################################################

# Ridge-CV

load(file = "ridge_cv_1_p_20_n_100.RData")
performance(list.ridge.cv[[2]],R)
```

```
# PCS-Ridge-CV

load(file = "pcs_ridge_cv_1_p_20_n_100.RData")
performance(list.ridge.cv.pcs[[2]],R)

######################################################
######################################################
######################################################
######################################################
######################################################
######################################################
######################################################
######################################################
######################################################
######################################################
######################################################
######################################################
######################################################
######################################################
######################################################

############################################################
##### HeatMaps
############################################################

corrplot(Adj_mat(R),method='color',is.corr = FALSE,
cl.lim=c(0,1), col=colorRampPalette(c("blue","white","black"))(200))

######################################################
######################################################

# Glasso-CV1

load(file = "glasso_cv_1_p_20_n_100.RData")
corrplot(Adj_mat(list.glasso.cv),method='color',is.corr = FALSE,
cl.lim=c(0,1), col=colorRampPalette(c("blue","white","black"))(200))

# PCS-Glasso-CV1

load(file = "pcs_glasso_cv_1_p_20_n_100.RData")
corrplot(Adj_mat(list.glasso.cv.pcs[[2]]),method='color',is.corr = FALSE,
cl.lim=c(0,1), col=colorRampPalette(c("blue","white","black"))(200))

######################################################
######################################################

# Glasso-CV1-1se

load(file = "glasso_cv_ls1_1_p_20_n_100.RData")
corrplot(Adj_mat(list.glasso.cv.ls1),method='color',is.corr = FALSE,
cl.lim=c(0,1), col=colorRampPalette(c("blue","white","black"))(200))

# PCS-Glasso-CV1-1se

load(file = "pcs_glasso_cv_ls1_1_p_20_n_100.RData")
corrplot(Adj_mat(list.glasso.cv.ls1.pcs[[2]]),method='color',is.corr = FALSE,
cl.lim=c(0,1), col=colorRampPalette(c("blue","white","black"))(200))

######################################################
######################################################

# Glasso-CV2

load(file = "glasso_cv2_1_p_20_n_100.RData")
```

```
corrplot(Adj_mat(list.glasso.cv2),method='color',is.corr = FALSE,
cl.lim=c(0,1), col=colorRampPalette(c("blue","white","black"))(200))

# PCS-Glasso-CV2

load(file = "pcs_glasso_cv2_1_p_20_n_100.RData")
corrplot(Adj_mat(list.glasso.cv2.pcs[[2]]),method='color',is.corr = FALSE,
cl.lim=c(0,1), col=colorRampPalette(c("blue","white","black"))(200))

######################################################
######################################################

# Glasso-CV2-1se

load(file = "glasso_cv2_ls1_1_p_20_n_100.RData")
corrplot(Adj_mat(list.glasso.cv2.ls1),method='color',is.corr = FALSE,
cl.lim=c(0,1), col=colorRampPalette(c("blue","white","black"))(200))

# PCS-Glasso-CV2-1se

load(file = "pcs_glasso_cv2_ls1_1_p_20_n_100.RData")
corrplot(Adj_mat(list.glasso.cv2.ls1.pcs[[2]]),method='color',is.corr = FALSE,
cl.lim=c(0,1), col=colorRampPalette(c("blue","white","black"))(200))

######################################################
######################################################

# Glasso-EBIC

load(file = "glasso_ebic_1_p_20_n_100.RData")
corrplot(Adj_mat(list.glasso.ebic),method='color',is.corr = FALSE,
cl.lim=c(0,1), col=colorRampPalette(c("blue","white","black"))(200))

# PCS-Glasso-EBIC

load(file = "pcs_glasso_ebic_1_p_20_n_100.RData")
corrplot(Adj_mat(list.glasso.ebic.pcs[[2]]),method='color',is.corr = FALSE,
cl.lim=c(0,1), col=colorRampPalette(c("blue","white","black"))(200))

######################################################
######################################################

# Glasso-BIC

load(file = "glasso_bic_1_p_20_n_100.RData")
corrplot(Adj_mat(list.glasso.bic),method='color',is.corr = FALSE,
cl.lim=c(0,1), col=colorRampPalette(c("blue","white","black"))(200))

# PCS-Glasso-BIC

load(file = "pcs_glasso_bic_1_p_20_n_100.RData")
corrplot(Adj_mat(list.glasso.bic.pcs[[2]]),method='color',is.corr = FALSE,
cl.lim=c(0,1), col=colorRampPalette(c("blue","white","black"))(200))

######################################################
######################################################

# SPACE-CV

load(file = "space_cv_1_p_20_n_100.RData")
corrplot(Adj_mat(list.space.cv[[2]]),method='color',is.corr = FALSE,
cl.lim=c(0,1), col=colorRampPalette(c("blue","white","black"))(200))

# PCS-SPACE-CV
```

```
load(file = "pcs_space_cv_1_p_20_n_100.RData")
corrplot(Adj_mat(list.space.cv.pcs[[2]]),method='color',is.corr = FALSE,
cl.lim=c(0,1), col=colorRampPalette(c("blue","white","black"))(200))

######################################################
######################################################

# SPACE-CV-1se

load(file = "space_cv_ls1_1_p_20_n_100.RData")
corrplot(Adj_mat(list.space.cv.ls1[[2]]),method='color',is.corr = FALSE,
cl.lim=c(0,1), col=colorRampPalette(c("blue","white","black"))(200))

# PCS-SPACE-CV-1se

load(file = "pcs_space_cv_ls1_1_p_20_n_100.RData")
corrplot(Adj_mat(list.space.cv.ls1.pcs[[2]]),method='color',is.corr = FALSE,
cl.lim=c(0,1), col=colorRampPalette(c("blue","white","black"))(200))

######################################################
######################################################

# SPACE-FSR

load(file = "space_alpha_1_p_20_n_100.RData")
corrplot(Adj_mat(list.space.alpha[[2]]),method='color',is.corr = FALSE,
cl.lim=c(0,1), col=colorRampPalette(c("blue","white","black"))(200))

# PCS-SPACE-FSR

load(file = "pcs_space_alpha_1_p_20_n_100.RData")
corrplot(Adj_mat(list.space.alpha.pcs[[2]]),method='color',is.corr = FALSE,
cl.lim=c(0,1), col=colorRampPalette(c("blue","white","black"))(200))

######################################################
######################################################

# SPACE-BIC

load(file = "space_bic_1_p_20_n_100.RData")
corrplot(Adj_mat(list.space.bic[[2]]),method='color',is.corr = FALSE,
cl.lim=c(0,1), col=colorRampPalette(c("blue","white","black"))(200))

# PCS-SPACE-BIC

load(file = "pcs_space_bic_1_p_20_n_100.RData")
corrplot(Adj_mat(list.space.bic.pcs[[2]]),method='color',is.corr = FALSE,
cl.lim=c(0,1), col=colorRampPalette(c("blue","white","black"))(200))

######################################################
######################################################

# NR-AND-CV

load(file = "nei_and_cv_1_p_20_n_100.RData")
corrplot(Adj_mat(list.nei.cv[[2]]),method='color',is.corr = FALSE,
cl.lim=c(0,1), col=colorRampPalette(c("blue","white","black"))(200))

# PCS-NR-AND-CV

load(file = "pcs_nei_and_cv_1_p_20_n_100.RData")
corrplot(Adj_mat(list.nei.cv.pcs[[2]]),method='color',is.corr = FALSE,
cl.lim=c(0,1), col=colorRampPalette(c("blue","white","black"))(200))
```

```
########################################################
########################################################

# NR-AND-CV-1se

load(file = "nei_and_cv_ls1_1_p_20_n_100.RData")
corrplot(Adj_mat(list.nei.cv.ls1[[2]]),method='color',is.corr = FALSE,
cl.lim=c(0,1), col=colorRampPalette(c("blue","white","black"))(200))

# PCS-NR-AND-CV-1se

load(file = "pcs_nei_and_cv_ls1_1_p_20_n_100.RData")
corrplot(Adj_mat(list.nei.cv.ls1.pcs[[2]]),method='color',is.corr = FALSE,
cl.lim=c(0,1), col=colorRampPalette(c("blue","white","black"))(200))

########################################################
########################################################

# NR-AND-FSR

load(file = "nei_and_alpha_1_p_20_n_100.RData")
corrplot(Adj_mat(list.nei.alpha[[2]]),method='color',is.corr = FALSE,
cl.lim=c(0,1), col=colorRampPalette(c("blue","white","black"))(200))

# PCS-NR-AND-FSR

load(file = "pcs_nei_and_alpha_1_p_20_n_100.RData")
corrplot(Adj_mat(list.nei.alpha.pcs[[2]]),method='color',is.corr = FALSE,
cl.lim=c(0,1), col=colorRampPalette(c("blue","white","black"))(200))

########################################################
########################################################

# NR-AND-BIC

load(file = "nei_and_bic_1_p_20_n_100.RData")
corrplot(Adj_mat(list.nei.bic[[2]]),method='color',is.corr = FALSE,
cl.lim=c(0,1), col=colorRampPalette(c("blue","white","black"))(200))

# PCS-NR-AND-BIC

load(file = "pcs_nei_and_bic_1_p_20_n_100.RData")
corrplot(Adj_mat(list.nei.bic.pcs[[2]]),method='color',is.corr = FALSE,
cl.lim=c(0,1), col=colorRampPalette(c("blue","white","black"))(200))

########################################################
########################################################

# NR-OR-CV

load(file = "nei_or_cv_1_p_20_n_100.RData")
corrplot(Adj_mat(list.nei.cv[[2]]),method='color',is.corr = FALSE,
cl.lim=c(0,1), col=colorRampPalette(c("blue","white","black"))(200))

# PCS-NR-OR-CV

load(file = "pcs_nei_or_cv_1_p_20_n_100.RData")
corrplot(Adj_mat(list.nei.cv.pcs[[2]]),method='color',is.corr = FALSE,
cl.lim=c(0,1), col=colorRampPalette(c("blue","white","black"))(200))

########################################################
########################################################
```

```
# NR-OR-CV-1se

load(file = "nei_or_cv_ls1_1_p_20_n_100.RData")
corrplot(Adj_mat(list.nei.cv.ls1[[2]]),method='color',is.corr = FALSE,
cl.lim=c(0,1), col=colorRampPalette(c("blue","white","black"))(200))

# PCS-NR-OR-CV-1se

load(file = "pcs_nei_or_cv_ls1_1_p_20_n_100.RData")
corrplot(Adj_mat(list.nei.cv.ls1.pcs[[2]]),method='color',is.corr = FALSE,
cl.lim=c(0,1), col=colorRampPalette(c("blue","white","black"))(200))

######################################################
######################################################

# NR-OR-FSR

load(file = "nei_or_alpha_1_p_20_n_100.RData")
corrplot(Adj_mat(list.nei.alpha[[2]]),method='color',is.corr = FALSE,
cl.lim=c(0,1), col=colorRampPalette(c("blue","white","black"))(200))

# PCS-NR-OR-FSR

load(file = "pcs_nei_or_alpha_1_p_20_n_100.RData")
corrplot(Adj_mat(list.nei.alpha.pcs[[2]]),method='color',is.corr = FALSE,
cl.lim=c(0,1), col=colorRampPalette(c("blue","white","black"))(200))

######################################################
######################################################

# NR-OR-BIC

load(file = "nei_or_bic_1_p_20_n_100.RData")
corrplot(Adj_mat(list.nei.bic[[2]]),method='color',is.corr = FALSE,
cl.lim=c(0,1), col=colorRampPalette(c("blue","white","black"))(200))

# PCS-NR-OR-BIC

load(file = "pcs_nei_or_bic_1_p_20_n_100.RData")
corrplot(Adj_mat(list.nei.bic.pcs[[2]]),method='color',is.corr = FALSE,
cl.lim=c(0,1), col=colorRampPalette(c("blue","white","black"))(200))

######################################################
######################################################

# Ridge-CV

load(file = "ridge_cv_1_p_20_n_100.RData")
corrplot(Adj_mat(list.ridge.cv[[2]]),method='color',is.corr = FALSE,
cl.lim=c(0,1), col=colorRampPalette(c("blue","white","black"))(200))

# PCS-Ridge-CV

load(file = "pcs_ridge_cv_1_p_20_n_100.RData")
corrplot(Adj_mat(list.ridge.cv.pcs[[2]]),method='color',is.corr = FALSE,
cl.lim=c(0,1), col=colorRampPalette(c("blue","white","black"))(200))

######################################################
######################################################
######################################################
######################################################
######################################################
######################################################
######################################################
```

```
######################################################
######################################################
######################################################
######################################################
######################################################
######################################################
######################################################
######################################################

############################################################
##### Networks
############################################################

qgraph(Omega.GGM(x,Adj_mat(R)), graph = "cor", fade=FALSE)

######################################################
######################################################

# Glasso-CV1

load(file = "glasso_cv_1_p_20_n_100.RData")
qgraph(Omega.GGM(x,Adj_mat(list.glasso.cv)), graph = "cor", fade=FALSE)

# PCS-Glasso-CV1

load(file = "pcs_glasso_cv_1_p_20_n_100.RData")
qgraph(Omega.GGM(x,Adj_mat(list.glasso.cv.pcs[[2]])), graph = "cor", fade=FALSE)

######################################################
######################################################

# Glasso-CV1-1se

load(file = "glasso_cv_ls1_1_p_20_n_100.RData")
qgraph(Omega.GGM(x,Adj_mat(list.glasso.cv.ls1)), graph = "cor", fade=FALSE)

# PCS-Glasso-CV1-1se

load(file = "pcs_glasso_cv_ls1_1_p_20_n_100.RData")
qgraph(Omega.GGM(x,Adj_mat(list.glasso.cv.ls1.pcs[[2]])), graph = "cor",
fade=FALSE)

######################################################
######################################################

# Glasso-CV2

load(file = "glasso_cv2_1_p_20_n_100.RData")
qgraph(Omega.GGM(x,Adj_mat(list.glasso.cv2)), graph = "cor", fade=FALSE)

# PCS-Glasso-CV2

load(file = "pcs_glasso_cv2_1_p_20_n_100.RData")
qgraph(Omega.GGM(x,Adj_mat(list.glasso.cv2.pcs[[2]])), graph = "cor",
fade=FALSE)

######################################################
######################################################

# Glasso-CV2-1se

load(file = "glasso_cv2_ls1_1_p_20_n_100.RData")
qgraph(Omega.GGM(x,Adj_mat(list.glasso.cv2.ls1)), graph = "cor", fade=FALSE)
```

```
# PCS-Glasso-CV2-1se

load(file = "pcs_glasso_cv2_ls1_1_p_20_n_100.RData")
qgraph(Omega.GGM(x,Adj_mat(list.glasso.cv2.ls1.pcs[[2]])), graph = "cor",
fade=FALSE)

######################################################
######################################################

# Glasso-EBIC

load(file = "glasso_ebic_1_p_20_n_100.RData")
qgraph(Omega.GGM(x,Adj_mat(list.glasso.ebic)), graph = "cor", fade=FALSE)

# PCS-Glasso-EBIC

load(file = "pcs_glasso_ebic_1_p_20_n_100.RData")
qgraph(Omega.GGM(x,Adj_mat(list.glasso.ebic.pcs[[2]])), graph = "cor",
fade=FALSE)

######################################################
######################################################

# Glasso-BIC

load(file = "glasso_bic_1_p_20_n_100.RData")
qgraph(Omega.GGM(x,Adj_mat(list.glasso.bic)), graph = "cor", fade=FALSE)

# PCS-Glasso-BIC

load(file = "pcs_glasso_bic_1_p_20_n_100.RData")
qgraph(Omega.GGM(x,Adj_mat(list.glasso.bic.pcs[[2]])), graph = "cor",
fade=FALSE)

######################################################
######################################################

# SPACE-CV

load(file = "space_cv_1_p_20_n_100.RData")
qgraph(Omega.GGM(x,Adj_mat(list.space.cv[[2]])), graph = "cor", fade=FALSE)

# PCS-SPACE-CV

load(file = "pcs_space_cv_1_p_20_n_100.RData")
qgraph(Omega.GGM(x,Adj_mat(list.space.cv.pcs[[2]])), graph = "cor", fade=FALSE)

######################################################
######################################################

# SPACE-CV-1se

load(file = "space_cv_ls1_1_p_20_n_100.RData")
qgraph(Omega.GGM(x,Adj_mat(list.space.cv.ls1[[2]])), graph = "cor", fade=FALSE)

# PCS-SPACE-CV-1se

load(file = "pcs_space_cv_ls1_1_p_20_n_100.RData")
qgraph(Omega.GGM(x,Adj_mat(list.space.cv.ls1.pcs[[2]])), graph = "cor",
fade=FALSE)

######################################################
######################################################
```

```
# SPACE-FSR

load(file = "space_alpha_1_p_20_n_100.RData")
qgraph(Omega.GGM(x,Adj_mat(list.space.alpha[[2]])), graph = "cor", fade=FALSE)

# PCS-SPACE-FSR

load(file = "pcs_space_alpha_1_p_20_n_100.RData")
qgraph(Omega.GGM(x,Adj_mat(list.space.alpha.pcs[[2]])), graph = "cor",
fade=FALSE)

######################################################
######################################################

# SPACE-BIC

load(file = "space_bic_1_p_20_n_100.RData")
qgraph(Omega.GGM(x,Adj_mat(list.space.bic[[2]])), graph = "cor", fade=FALSE)

# PCS-SPACE-BIC

load(file = "pcs_space_bic_1_p_20_n_100.RData")
qgraph(Omega.GGM(x,Adj_mat(list.space.bic.pcs[[2]])), graph = "cor", fade=FALSE)

######################################################
######################################################

# NR-AND-CV

load(file = "nei_and_cv_1_p_20_n_100.RData")
qgraph(Omega.GGM(x,Adj_mat(list.nei.cv[[2]])), graph = "cor", fade=FALSE)

# PCS-NR-AND-CV

load(file = "pcs_nei_and_cv_1_p_20_n_100.RData")
qgraph(Omega.GGM(x,Adj_mat(list.nei.cv.pcs[[2]])), graph = "cor", fade=FALSE)

######################################################
######################################################

# NR-AND-CV-1se

load(file = "nei_and_cv_ls1_1_p_20_n_100.RData")
qgraph(Omega.GGM(x,Adj_mat(list.nei.cv.ls1[[2]])), graph = "cor", fade=FALSE)

# PCS-NR-AND-CV-1se

load(file = "pcs_nei_and_cv_ls1_1_p_20_n_100.RData")
qgraph(Omega.GGM(x,Adj_mat(list.nei.cv.ls1.pcs[[2]])), graph = "cor",
fade=FALSE)

######################################################
######################################################

# NR-AND-FSR

load(file = "nei_and_alpha_1_p_20_n_100.RData")
qgraph(Omega.GGM(x,Adj_mat(list.nei.alpha[[2]])), graph = "cor", fade=FALSE)

# PCS-NR-AND-FSR

load(file = "pcs_nei_and_alpha_1_p_20_n_100.RData")
qgraph(Omega.GGM(x,Adj_mat(list.nei.alpha.pcs[[2]])), graph = "cor", fade=FALSE)
```

```
########################################################
########################################################

# NR-AND-BIC

load(file = "nei_and_bic_1_p_20_n_100.RData")
qgraph(Omega.GGM(x,Adj_mat(list.nei.bic[[2]])), graph = "cor", fade=FALSE)

# PCS-NR-AND-BIC

load(file = "pcs_nei_and_bic_1_p_20_n_100.RData")
qgraph(Omega.GGM(x,Adj_mat(list.nei.bic.pcs[[2]])), graph = "cor", fade=FALSE)

########################################################
########################################################

# NR-OR-CV

load(file = "nei_or_cv_1_p_20_n_100.RData")
qgraph(Omega.GGM(x,Adj_mat(list.nei.cv[[2]])), graph = "cor", fade=FALSE)

# PCS-NR-OR-CV

load(file = "pcs_nei_or_cv_1_p_20_n_100.RData")
qgraph(Omega.GGM(x,Adj_mat(list.nei.cv.pcs[[2]])), graph = "cor", fade=FALSE)

########################################################
########################################################

# NR-OR-CV-1se

load(file = "nei_or_cv_ls1_1_p_20_n_100.RData")
qgraph(Omega.GGM(x,Adj_mat(list.nei.cv.ls1[[2]])), graph = "cor", fade=FALSE)

# PCS-NR-OR-CV-1se

load(file = "pcs_nei_or_cv_ls1_1_p_20_n_100.RData")
qgraph(Omega.GGM(x,Adj_mat(list.nei.cv.ls1.pcs[[2]])), graph = "cor",
fade=FALSE)

########################################################
########################################################

# NR-OR-FSR

load(file = "nei_or_alpha_1_p_20_n_100.RData")
qgraph(Omega.GGM(x,Adj_mat(list.nei.alpha[[2]])), graph = "cor", fade=FALSE)

# PCS-NR-OR-FSR

load(file = "pcs_nei_or_alpha_1_p_20_n_100.RData")
qgraph(Omega.GGM(x,Adj_mat(list.nei.alpha.pcs[[2]])), graph = "cor", fade=FALSE)

########################################################
########################################################

# NR-OR-BIC

load(file = "nei_or_bic_1_p_20_n_100.RData")
qgraph(Omega.GGM(x,Adj_mat(list.nei.bic[[2]])), graph = "cor", fade=FALSE)

# PCS-NR-OR-BIC

load(file = "pcs_nei_or_bic_1_p_20_n_100.RData")
```

```
qgraph(Omega.GGM(x,Adj_mat(list.nei.bic.pcs[[2]])), graph = "cor", fade=FALSE)

######################################################
######################################################

# Ridge-CV

load(file = "ridge_cv_1_p_20_n_100.RData")
qgraph(solve(list.ridge.cv[[2]]), graph = "pcor", fade=FALSE)

# PCS-Ridge-CV

load(file = "pcs_ridge_cv_1_p_20_n_100.RData")
qgraph(Omega.GGM(x,Adj_mat(list.ridge.cv.pcs[[2]])), graph = "cor", fade=FALSE)


##############################################################
##############################################################
##############################################################
##############################################################
##############################################################
##############################################################
##############################################################
##############################################################
##############################################################
##############################################################

############################################################
##### PCS-Networks Combination
############################################################

# Create a matrix that combines the 19 PCS estimates

p = ncol(x)

Adj_mat_average = matrix(0,p,p)

#############################################################

load(file = "PCS_glasso_cv_1_p_20_n_100.RData")
Adj_mat_average = Adj_mat(list.glasso.cv.pcs[[2]])

#############################################################

load(file = "PCS_glasso_cv_ls1_1_p_20_n_100.RData")
Adj_mat_average = Adj_mat_average + Adj_mat(list.glasso.cv.ls1.pcs[[2]])

#############################################################

load(file = "PCS_glasso_cv2_1_p_20_n_100.RData")
Adj_mat_average = Adj_mat(list.glasso.cv.pcs[[2]])

#############################################################

load(file = "PCS_glasso_cv2_ls1_1_p_20_n_100.RData")
Adj_mat_average = Adj_mat_average + Adj_mat(list.glasso.cv2.ls1.pcs[[2]])

#############################################################

load(file = "PCS_glasso_bic_1_p_20_n_100.RData")
Adj_mat_average = Adj_mat_average + Adj_mat(list.glasso.bic.pcs[[2]])

#############################################################
```

```
load(file = "PCS_glasso_ebic_1_p_20_n_100.RData")
Adj_mat_average = Adj_mat_average + Adj_mat(list.glasso.ebic.pcs[[2]])

#############################################################

load(file = "PCS_space_cv_1_p_20_n_100.RData")
Adj_mat_average = Adj_mat_average + Adj_mat(list.space.cv.pcs[[2]])

#############################################################

load(file = "PCS_space_cv_ls1_1_p_20_n_100.RData")
Adj_mat_average = Adj_mat_average + Adj_mat(list.space.cv.ls1.pcs[[2]])

#############################################################

load(file = "PCS_space_alpha_1_p_20_n_100.RData")
Adj_mat_average = Adj_mat_average + Adj_mat(list.space.alpha.pcs[[2]])

#############################################################

load(file = "PCS_space_bic_1_p_20_n_100.RData")
Adj_mat_average = Adj_mat_average + Adj_mat(list.space.bic.pcs[[2]])

#############################################################

load(file = "PCS_nei_and_cv_1_p_20_n_100.RData")
Adj_mat_average = Adj_mat_average + Adj_mat(list.nei.cv.pcs[[2]])

#############################################################

load(file = "PCS_nei_and_cv_ls1_1_p_20_n_100.RData")
Adj_mat_average = Adj_mat_average + Adj_mat(list.nei.cv.ls1.pcs[[2]])

#############################################################

load(file = "PCS_nei_and_alpha_1_p_20_n_100.RData")
Adj_mat_average = Adj_mat_average + Adj_mat(list.nei.alpha.pcs[[2]])

#############################################################

load(file = "PCS_nei_and_bic_1_p_20_n_100.RData")
Adj_mat_average = Adj_mat_average + Adj_mat(list.nei.bic.pcs[[2]])

#############################################################

load(file = "PCS_nei_or_cv_1_p_20_n_100.RData")
Adj_mat_average = Adj_mat_average + Adj_mat(list.nei.cv.pcs[[2]])

#############################################################

load(file = "PCS_nei_or_cv_ls1_1_p_20_n_100.RData")
Adj_mat_average = Adj_mat_average + Adj_mat(list.nei.cv.ls1.pcs[[2]])

#############################################################

load(file = "PCS_nei_or_alpha_1_p_20_n_100.RData")
Adj_mat_average = Adj_mat_average + Adj_mat(list.nei.alpha.pcs[[2]])

#############################################################

load(file = "PCS_nei_or_bic_1_p_20_n_100.RData")
Adj_mat_average = Adj_mat_average + Adj_mat(list.nei.bic.pcs[[2]])

#############################################################
```

```
load(file = "PCS_ridge_cv_1_p_20_n_100.RData")
Adj_mat_average = Adj_mat_average + Adj_mat(list.ridge.cv.pcs[[2]])

##############################################################
##############################################################
##############################################################
##############################################################

# Generate a matrix that contains the edges with a frequency of appearance of at
least in two of the estimation procedures

Adj_mat_average = Adj_mat_average

for (i in 1:p){
for (j in 1:p){
if (abs(Adj_mat_average[i,j])<=1){Adj_mat_average[i,j]=0}
else (Adj_mat_average[i,j]=1)
}}

# Compute Partial Correlation MAtrix with the combination of the models

R.combination = Omega.GGM(x,Adj_mat_average)

# Network plot

qgraph(R.combination, graph = "cor", layout = "circular", fade=FALSE)

# Heatmap

corrplot(Adj_mat(R.combination),method='color',is.corr = FALSE,
cl.lim=c(0,1), col=colorRampPalette(c("blue","white","black"))(200))
```
